# Genome-culture coevolution promotes rapid divergence of killer whale ecotypes

**DOI:** 10.1038/ncomms11693

**Published:** 2016-05-31

**Authors:** Andrew D. Foote, Nagarjun Vijay, María C. Ávila-Arcos, Robin W. Baird, John W. Durban, Matteo Fumagalli, Richard A. Gibbs, M. Bradley Hanson, Thorfinn S. Korneliussen, Michael D. Martin, Kelly M. Robertson, Vitor C. Sousa, Filipe G. Vieira, Tomáš Vinař, Paul Wade, Kim C. Worley, Laurent Excoffier, Phillip A. Morin, M. Thomas P. Gilbert, Jochen B.W. Wolf

**Affiliations:** 1 Department of Evolutionary Biology, Evolutionary Biology Centre, Uppsala University, Norbyvägen 18D, Uppsala SE-752 36, Sweden; 2 Centre for GeoGenetics, Natural History Museum of Denmark, University of Copenhagen, Øster Volgade 5-7, Copenhagen K 1350, Denmark; 3 Computational and Molecular Population Genetics Laboratory, Institute of Ecology and Evolution, University of Bern, Baltzerstrasse 6, Bern 3012, Switzerland; 4 Department of Genetics, Stanford University, Stanford, California 94305, USA; 5 Cascadia Research, 4th Avenue, Olympia, Washington 98501, USA; 6 Marine Mammal and Turtle Division, Southwest Fisheries Science Center, National Marine Fisheries Service, National Oceanographic and Atmospheric Administration, 8901 La Jolla Shores Drive, La Jolla, California 92037, USA; 7 Department of Genetics, Evolution, and Environment, UCL Genetics Institute, University College London, London WC1E 6BT, UK; 8 Department of Molecular and Human Genetics, Human Genome Sequencing Center, Baylor College of Medicine, One Baylor Plaza, Houston, Texas 77030, USA; 9 Northwest Fisheries Science Center, National Marine Fisheries Service, National Oceanic and Atmospheric Administration, 2725 Montlake Boulevard East, Seattle, Washington 98112, USA; 10 Faculty of Mathematics, Physics and Informatics, Comenius University, Mlynska Dolina, Bratislava 84248, Slovakia; 11 National Marine Mammal Laboratory, Alaska Fisheries Science Center, National Marine Fisheries Service, National Oceanic and Atmospheric Administration, 7600 Sand Point Way NE, Seattle, Washington 98115, USA; 12 Department of Environment and Agriculture, Trace and Environmental DNA Laboratory, Curtin University, Perth, Western Australia 6102, Australia; 13 Department of Evolutionary Biology, Science for Life Laboratory, Evolutionary Biology Centre, Uppsala University, Uppsala 75236, Sweden; 14 Section of Evolutionary Biology, Department of Biology II, Ludwig Maximilian University of Munich, Großhaderner Strasse 2, Planegg-Martinsried 82152, Germany

## Abstract

Analysing population genomic data from killer whale ecotypes, which we estimate have globally radiated within less than 250,000 years, we show that genetic structuring including the segregation of potentially functional alleles is associated with socially inherited ecological niche. Reconstruction of ancestral demographic history revealed bottlenecks during founder events, likely promoting ecological divergence and genetic drift resulting in a wide range of genome-wide differentiation between pairs of allopatric and sympatric ecotypes. Functional enrichment analyses provided evidence for regional genomic divergence associated with habitat, dietary preferences and post-zygotic reproductive isolation. Our findings are consistent with expansion of small founder groups into novel niches by an initial plastic behavioural response, perpetuated by social learning imposing an altered natural selection regime. The study constitutes an important step towards an understanding of the complex interaction between demographic history, culture, ecological adaptation and evolution at the genomic level.

## Abstract (web summary)

Killer whales have evolved into specialized ecotypes based on hunting strategies and ecological niches. Here, Andrew Foote and colleagues sequenced the whole genome of individual killer whales representing 5 different ecotypes from North Pacific and Antarctic, and show expansion of small founder groups to adapt to specific ecological niches.

## Graphical abstract

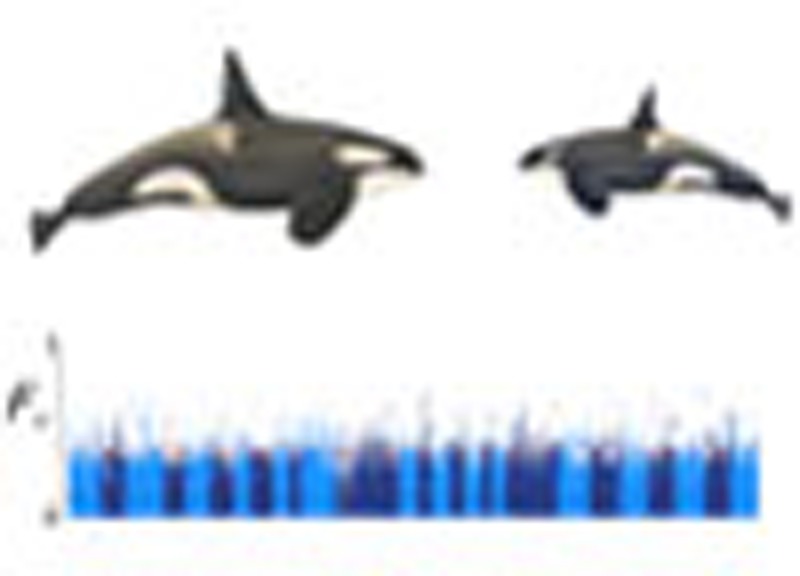

The interplay between ecology, culture and evolution at the level of the genome remains poorly understood^1^. The ability to adapt to novel ecological conditions through behavioural plasticity is thought to be able to buffer natural selection pressures and promote rapid colonization of novel niches^1^. However, by perpetuating exposure to a novel environment, stable cultural transmission of behaviour can also provide an opportunity for natural selection to act on adaptive genomic variation. Examples of genomic adaptation in humans during the period of recent ecological and cultural diversification and consequent demographic expansion are well illustrated^1^,^2^,^3^. For example, the Inuit of Greenland descend from a small founder population that split from an East Asian source population and successfully colonized the extreme climatic conditions of the Arctic environment through culturally transmitted methods of hunting marine mammals and genetic adaptation to a cold climate and hypoglycaemic lipid-rich diet^4^. However, our understanding of the complex interaction between ecology, culture, adaptation and reproductive isolation at a genome-wide level has long suffered from deficiency of genome-wide data, and, conceptually, from the almost-exclusive focus on these processes in humans and thus a lack of comparative data from other species^5^.

Killer whales (*Orcinus orca*) are the largest species in the dolphin family (Delphinidae) and, together with humans, are one of the most cosmopolitan mammals, being found in all ocean basins and distributed from the Antarctic to the Arctic^6^. This top marine predator consumes a diverse range of prey species, including birds, fish, mammals and reptiles^6^. However, in several locations killer whales have evolved into specialized ecotypes, with hunting strategies adapted to exploit narrow ecological niches^6^,^7^,^8^,^9^,^10^ (see Supplementary Notes for more detailed information on the natural history of killer whale ecotypes). For example, in the North Pacific, two sympatric ecotypes coexist in coastal waters: the mammal-eating (so-called ‘*transient*') ecotype and fish-eating (so-called ‘*resident*') ecotype^7^,^8^. This ecotypic variation is stable among multiple subpopulations of the *transient* and *resident* ecotypes across the North Pacific that diverged from common ancestral matrilines ∼68 and 35 KYA, respectively^11^. A highly stable matrilineal group structure and a long post-menopausal lifespan in killer whales is thought to facilitate the transfer of ecological and social knowledge from matriarchs to their kin^12^, and thereby perpetuate the stability of ecotypic variation in killer whales^13^. In the absence of a definitional consensus and for the purposes of investigating how cultural phenomena interact with genes, culture has been broadly defined as ‘*information that is capable of affecting individuals' behaviour, which they acquire from other individuals through teaching, imitation and other forms of social learning*'^1^. Several studies have argued that behavioural differences among killer whale ecotypes are examples of culture in this broader sense of the term^13^,^14^. However, this behavioural variation among ecotypes likely results from ecological, genetic and cultural variation and the interaction between them, rather than a single process explaining all behavioural variance^15^. Killer whales, therefore, offer a prime example of how behavioural innovation perpetuated by cultural transmission may have enabled access to novel ecological conditions with altered selection regimes, and thus provide an excellent study system for understanding the interaction between ecological and behavioural variation, and genome-level evolution.

## Results and Discussion

### Whole-genome sequencing

**Figure 1:**
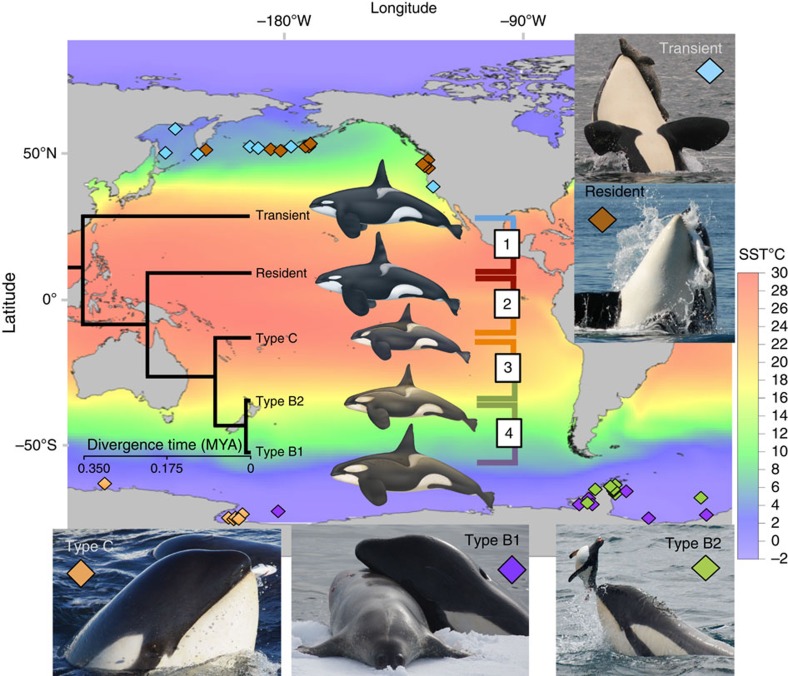
Map of sampling locations of the five killer whale types included in this study. Sampling locations and inset photographs illustrating favoured prey species are colour-coded by ecotype: ‘*transient*' (blue) and *type B1* (purple) are predominantly mammal-eating; ‘*resident*' (brown) and *type C* (orange) are predominantly fish-eating; *type B2* (green) is known to feed on penguins. The map is superimposed on a colour grid of sea-surface temperature (SST). The Antarctic ecotypes primarily inhabit waters 8–16 °C colder than the North Pacific ecotypes. The relationship among these types and their estimated divergence times based on mitochondrial genomes are shown in the superimposed chronogram. Boxes 1–4 indicate pairwise comparisons spanning points along the ‘speciation continuum' used to investigate the build up of genomic differentiation. (Photo credits: Dave Ellifrit, Center for Whale Research; Holly Fearnbach and Robert Pitman, SWFSC; SST measurements from NOAA Optimum Interpolation SST V2 long-term mean 1981–2010, www.esrl.noaa.gov/psd/repository courtesy of Paul Fiedler; killer whale illustrations courtesy of Uko Gorter.)

We generated whole-genome re-sequencing data of 48 individuals at low coverage and accessed high-coverage sequencing data from two more individuals^16^,^17^ to investigate patterns of genomic variation among killer whale ecotypes. The samples represent five distinct ecotypes that, based on phylogenetic analysis of mitochondrial genomes^11^, include some of the oldest and youngest divergences within the species (Fig. 1). The dataset incorporated 10 individuals each of the *transient* and *resident* ecotypes that occur in sympatry in the North Pacific; and from Antarctic waters, 7 ndividuals of a large mammal-eating form (*type B1*), 11 individuals of a partially sympatric, smaller form which feeds on penguins (*type B2*), and 10 individuals of the smallest form of killer whale, which feeds on fish (type C) (Fig. 1). A total of 2,577 million reads uniquely mapped to the 2.4-Gbp killer whale reference genome^16^ (Supplementary Fig. 1) for which a chromosomal assembly was generated for this study, so that approximately 50% of the autosomal regions of each individual were sequenced at ≥2 × coverage (Supplementary Table 1). Subsequent data analyses used methods that account for uncertainty in the assignments of genotypes, enabling accurate inferences to be drawn from low-pass next-generation sequencing data^18^. Comparisons of estimated population genomic metrics such as genome-wide and per-site *F*_ST_ indicated that estimates from our low-coverage data were highly consistent with published high-coverage restriction site associated DNA sequencing (RAD-seq)^19^ and single-nucleotide polymorphism (SNP)-typing^11^ data (Supplementary Fig. 2 and Supplementary Table 2) and thus confirmed the robustness of our estimates.

### Time to most recent common ancestor

We estimated a time to most recent common ancestor (TMRCA) of ∼126–227 KYA from the accumulation of derived mutations at third-codon positions for the most divergent killer whale lineages compared here (Supplementary Table 3) and based on the 95% highest posterior density interval of the mutation rate estimate^20^. This equates to ∼4,900–8,800 generations and indicates a rapid diversification over a timescale comparable to the diversification of modern humans^21^. We caution that for these age estimates we rely on mutation rate estimates derived from interspecific comparisons among odontocetes, and that, therefore, these estimates of TMRCA are at best approximate. Further, the demographic history and any gene flow between ecotypes will have an influence on the sharing of derived mutations and hence this estimate. However, we do expect that the estimate will be within the correct order of magnitude. Our estimated TMCRA overlaps with a recent RAD marker study^22^, which estimated a TMRCA of 189 KYA (only a point estimate was reported by these authors) scaling by a mutation rate 1.21 times higher than we have used here. The estimated TMRCA of a global data set of killer whales based on non-recombining mitochondrial genomes has been estimated at 220–530 KYA (ref. 11), older than our estimate based on nuclear genomes. Male-mediated gene flow has therefore continued after matrilineal lineages have diverged.

### Genetic differentiation and divergence

**Figure 2:**
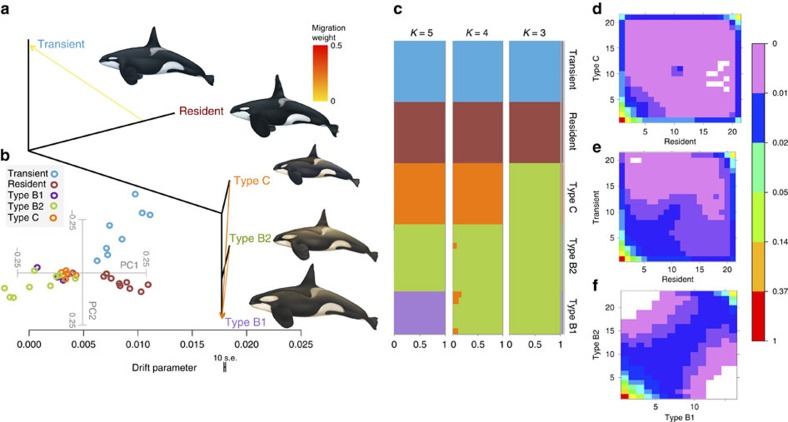
Evolutionary relationships among killer whale ecotypes. (**a**) TreeMix maximum likelihood graph from whole-genome sequencing data, rooted with the ‘*transient*' ecotype. Horizontal branch lengths are proportional to the amount of genetic drift that has occurred along that branch. The scale bar shows 10 times the average s.e. of the entries in the sample covariance matrix. Migration edges inferred using TreeMix and supported by the *f*3 statistic test are depicted as arrows coloured by migration weight. (**b**) PCA, both the first and second principal components were statistically significant (*P*-value <0.001). (**c**) Ancestry proportions for each of the 48 individuals conditional on the number of genetic clusters (*K*=3–5). (**d**–**f**) Joint site frequency spectra for pairwise comparisons illustrating the speciation continuum. Each entry in the matrix (*x*,*y*) corresponds to the probability of observing a SNP with frequency of derived allele *x* in population 1 and *y* in population 2. The colours represent the probability for each cell of the SFS, white cells correspond to a probability of zero. The analysis illustrates that the amount of genetic differentiation is greater between (**d**) Pacific and Antarctic populations and (**e**) the pair of Pacific types than (**f**) among the evolutionarily younger Antarctic types, which have highly correlated site frequency spectra.

Despite this recent shared ancestry, substantial genome-wide differentiation and divergence had accrued between all pairs of ecotypes included in this study (Fig. 2 and Supplementary Table 2). At *K*=5 populations, a maximum-likelihood-based clustering algorithm^23^ unambiguously assigned all individuals to populations corresponding to ecotype (Fig. 2c), indicating that all ecotypes have been assortatively mating long enough to allow allele frequencies to drift apart. Pairwise genetic distances between individuals visualized as a tree indicate that segregating alleles are largely shared within an ecotype (Supplementary Fig. 3). Similarly, pairwise relatedness due to identity-by-descent, that is, genetic identity because of a recent common ancestor, was high within each ecotype. While the three Antarctic ecotypes still showed signs of recent relatedness, no shared recent identity-by-descent ancestry was detected between Antarctic and Pacific types or between the sympatric *resident* and *transient* ecotypes (Supplementary Fig. 4). The greatest differentiation (Supplementary Table 2) as visualized in the maximum likelihood graph (Fig. 2a) and PCA plot (Fig. 2b) was between the allopatric Pacific and the Antarctic ecotypes, while differentiation among Antarctic ecotypes was much lower than between the two Pacific ecotypes. Thus, our sampled populations allowed us to investigate the accrual of genomic differentiation along points of a continuum, acting as a proxy of sampling at different stages of the speciation process. The accrual of genome-wide differentiation (*F*_ST_=0.09) between even the most recently diverged and partially sympatric ecotypes (Antarctic types *B1* and *B2*) indicates that reproductive isolation quickly becomes established after the formation of new ecotypes. Thus, whole-genome resolution confirms that, even in sympatry, contemporary gene flow occurs almost exclusively among individuals of the same ecotype, allowing genomic differentiation to build up between ecotypes so that within an ocean basin ecological variation better predicted genetic structuring than geography.

### Ancient admixture

To better understand and visualize the complexity of the ancestry of killer whale ecotypes, we reconstructed the genetic relationships among ecotypes in the form of a maximum likelihood graph (Fig. 2a), representing the degree of genetic drift and modelling both population splits and gene flow using the unified statistical framework implemented in TreeMix (ref. 24). The inferred migration edges were supported by the three-population (*f*3) and D-statistic (ABBA-BABA)^25^ tests, which can provide clear evidence of admixture, even if the gene flow events occurred hundreds of generations ago^26^. These population genomic methods test for asymmetry in the covariance of allele frequencies that indicate that the relationships among populations are not fully described by a simple bifurcating tree model.

The three approaches were consistent in inferring migration from source populations that share ancestry with the North Pacific *resident* and North Atlantic ecotype into the *transient* ecotype (Fig. 2a, Supplementary Figs 5 and 6, Supplementary Tables 4 and 5 and Supplementary Data 1). The genomes of *transients* are therefore partly derived from at least one population related to the Atlantic and *resident* ecotypes, (but not necessarily these populations, that is, the source could be an unsampled ‘ghost' population). The asymmetrical two-dimensional (2D)-site frequency spectrum (SFS) also implies directional gene flow from a population ancestrally related to the *residents* into the *transient* ecotype^27^ (Fig. 2e). Given the extent of the inferred demographic bottlenecks during founder events (see section below), and the expected consequential shift in the SFS^28^, it seems likely that this admixture would have occurred during or after the founder bottleneck in the *transient* ecotype, otherwise it would be expected to be less correlated with the *resident* SFS. The sequencing of more populations is expected to shed further light on this episode of ancient admixture.

TreeMix, the three-population and D-statistic tests inferred that *type B1* is admixed and derives from at least two populations related to both types *B2* and *C* (Fig. 2a, Supplementary Tables 4 and 5 and Supplementary Data 1). Conducting D-statistic tests on proposed tree-like histories comprising combinations of 16 genome sequences that included the North Atlantic sequence, we found that Antarctic types *B1* and *B2* shared an excess of alleles with the three Northern Hemisphere ecotypes (Supplementary Figs 7 and 8). This shared ancestry component between Northern and Southern hemisphere ecotypes was not detected in *type C*, suggesting a relatively recent admixture event after *type C* split from the shared ancestor of types *B1* and *B2*. Other signals of ancient admixture among populations were also detected and are reported in the tables in the Supplementary Material.

### Demographic history

**Figure 3:**
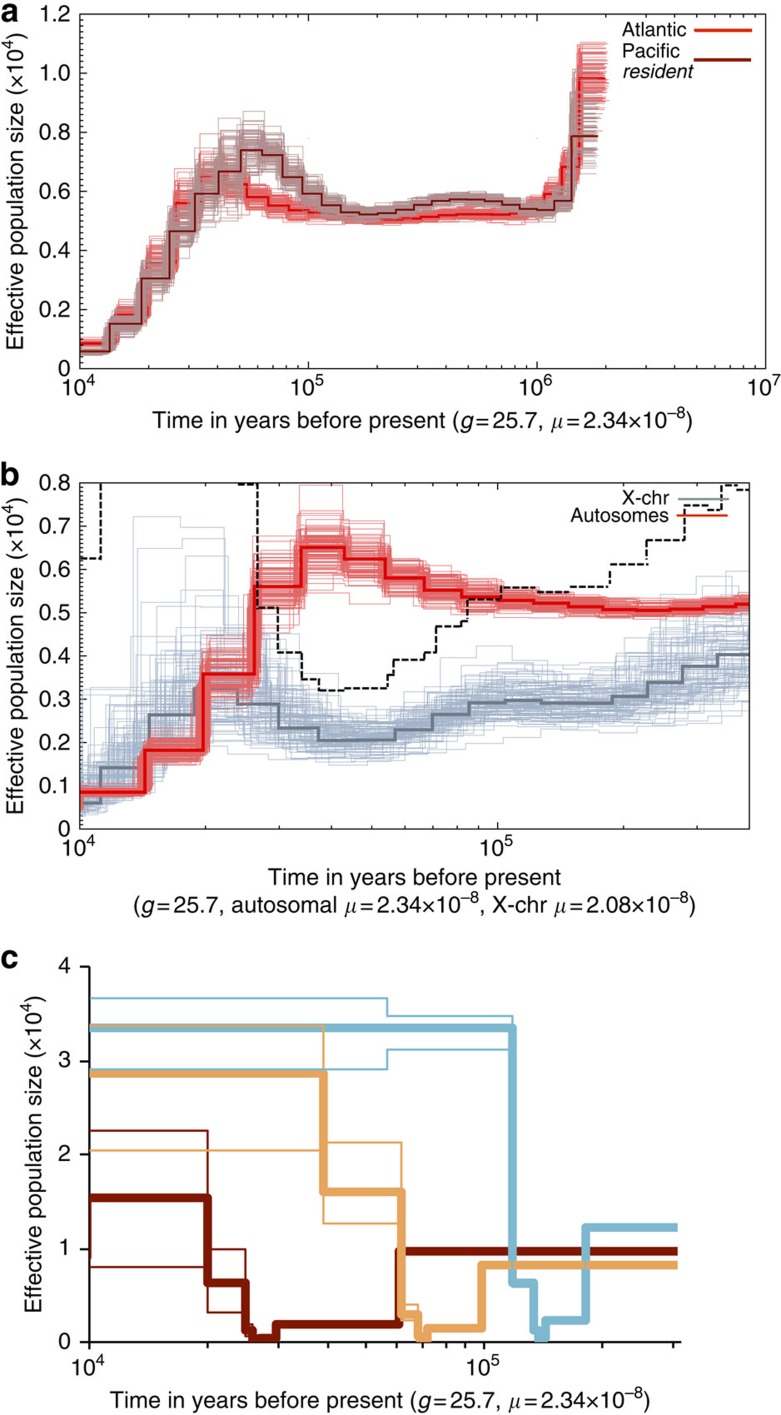
Reconstructing the demographic history of killer whale ecotypes. (**a**) PSMC estimates of changes in effective population size (*N*_e_) over time inferred from the autosomes of a North Atlantic killer whale (red) and from the autosomes of a North Pacific *resident* killer whale (brown). Thick lines represent the median and thin light lines of the same colour correspond to 100 rounds of bootstrapping. (**b**) PSMC estimates of changes in *N*_e_ over time inferred from the autosomes (*N*_eA_, red) and the X-chromosome (*N*_eX_, grey) of the high-coverage genome sequence of a North Atlantic female killer whale. Thick lines represent the median and thin light lines of the same colour correspond to 100 rounds of bootstrapping. The dashed black line indicates the ratio of *N*_eX_/*N*_eA_. (**c**) Changes in effective population size (*N*_e_) over time in the *transients* (blue), *residents* (brown) and *type C* (orange) inferred using the SFS of each ecotype. Thick lines represent the median and thin light lines the 2.5 and 97.5 percentiles of the SFS analysis.

As our results suggest that killer whale ecotypes have diversified rapidly from a recent ancestor, and given the importance of the relationship between effective population size (*N*_e_) and the rate of evolution^29^, we conducted analyses to reconstruct their demographic history. Applying the pairwise sequential Markovian coalescent (PSMC) approach^21^ to two high-coverage (≥20 × ) autosomal assemblies, a North Atlantic female and a North Pacific *resident* male, refining the methodological approach of a previous analysis of these genomes (Supplementary Figs 9 and 10), we recovered a similar demographic trajectory to that previously reported^17^ (Fig. 3a). The inference of the timing of these demographic declines is dependent on the assumed mutation rate (*μ*); however, across a range of sensible estimates of *μ*, the declines broadly fall within the Late Pleistocene^17^. This was previously interpreted as evidence for demographically independent population declines in each ocean, driven by environmental change during the Weichselian glacial period^17^. However, this inference assumes that each PSMC plot tracks the demographic history of a single unstructured panmictic population^30^. The *y* axis of the PSMC plot is an estimation of *N*_e_ derived from the rate of coalescence between the two chromosomes of a diploid genome. However, in the presence of population structuring, regions of the two chromosomes will coalesce less frequently as their ancestry may derive from different demes or subpopulations; the rate of coalescence can thus be similar to a single population with large *N*_e_. PSMC estimates of *N*_e_ during population splits can therefore be greater than the sum of the effective sizes of the subpopulations, dependent on the number of subpopulations and the degree of connectivity (that is, cross-coalescence) between them^21^,^30^. In fact, the results presented here and previously published^17^,^19^,^22^ indicate that throughout the Weichselian glacial period there were multiple population splits, both between and within ecotypes, including the splitting of the two lineages included in the PSMC analyses just at the point of the change in inferred *N*_e_ (Fig. 3a and Supplementary Fig 11). Therefore, the PSMC plots will be strongly influenced by these changes in structure, and the changes in inferred *N*_e_ are not necessarily associated with population declines (see Supplementary Fig. 12).

To investigate the influence of connectivity on our PSMC plots further, we inferred ancestral *N*_e_ of the diploid X-chromosome (*N*_eX_) of the Atlantic female and directly compared with the autosomal fraction (*N*_eA_) of the genome presented in Fig. 3a. From ∼300,000 to 130,000 years BP during the Saalian glacial period, the inferred *N*_eX_ ranged from 0.58 to 0.79 of the *N*_eA_ (Fig. 3b), which lies within expectation for demographically stable mammalian species^31^. During the first part of the Weichselian glacial period, *N*_eX_ markedly declined, reaching a minimum at ∼30,000–50,000 years BP. The timing of this bottleneck in *N*_eX_ overlaps with the stem age of the mitochondrial clade for this Atlantic population^11^, that is, consistent with almost all mitochondrial diversity being lost in this lineage during this period. Conversely, this is concurrent with the peak estimate of autosomal *N*_eA_ inferred by PSMC, and ratio of *N*_eX_/*N*_eA_ falls to ∼0.3 at this *N*_eX_ minimum and then recovers within 1,000 generations to >0.75 (Fig. 3b). Simulated demographic bottlenecks of several hundredfold reduction result in a disproportionate loss of *N*_eX_, attributed to the difference in the inheritance mode of each marker, and the ratio of *N*_eX_/*N*_eA_ can reach less than 0.3 (ref. 31). Following the bottleneck, *N*_eX_ recovers more rapidly than *N*_eA_ and the ratio of *N*_eX_/*N*_eA_ can exceed 0.75 during this recovery phase^31^. The concordance of the timing of the bottleneck in the X-chromosome and the stem age of the mitochondrial genome suggest an underlying demographic process, rather than a strong selection on the X-chromosome or some other factors driving the mutation rate^31^. A sex-biased process such as primarily male-mediated gene flow between demes could further influence the ratio of *N*_eX_/*N*_eA_ (ref. 32).

To estimate the demographic histories from our population genomic data, we produced ‘stairway' plots using composite likelihood estimations of theta (*θ*) for different SNP frequency spectra associated with different epochs, which are then scaled by the mutation rate to estimate *N*_e_ for each epoch^33^. Using this method we reconstructed a demographic history from our population genomic data for the *resident* ecotype that was comparable to the PSMC plot from a single *resident* high-coverage genome, both methods identifying a decline in *N*_e_ starting at ∼60 KYA (Fig. 3a,c). The stairway plots for the *transient* ecotype and *type C* showed the same pattern as the resident of a decline to a bottlenecked population with an *N*_e_ of <1,000 and a subsequent expansion (Fig. 3c). The bottlenecks did not occur simultaneously, as might be expected in response to a global environmental stressor during a glacial cycle, but instead were sequential (Fig. 3c). In each case, the timing of the demographic bottleneck overlapped with the previously estimated timing of the stem age of the mitochondrial genome clades containing each ecotype^11^. Thus, within the *transient*, *resident* and combined Antarctic ecotypes, both mitochondrial and nuclear lineages coalesce back to these respective bottleneck events, consistent with genetic isolation of small matrifocal founder groups from an ancestral source population, followed by the subsequent expansion and substructuring of a newly established ecotype.

Overall, the population genetic analyses of the whole-genome sequences above shed light on the ancestry of killer whale ecotypes in unprecedented detail, highlighting a complex tapestry of periods of isolation interspersed with episodic admixture events and strong demographic bottlenecks associated with the founder events that gave rise to the resident, transient and ancestral Antarctic ecotypes.

### Genome-wide landscape of genetic diversity and differentiation

**Figure 4:**
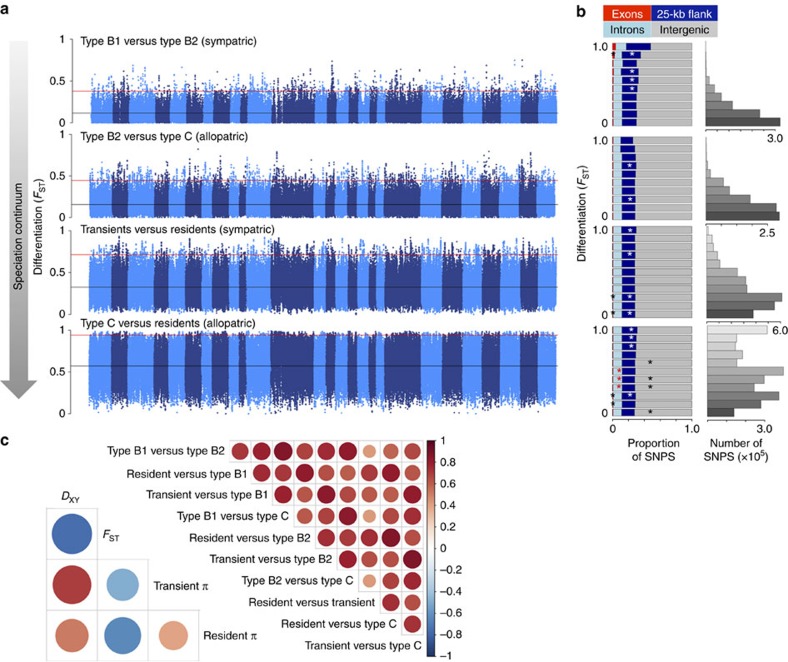
Genome-wide distribution of differentiation. (**a**) Pairwise genetic differentiation (*F*_ST_) in 50-kb sliding windows across the genome between killer whale ecotypes. Pairwise comparisons are arranged from the youngest divergence between sympatric ecotypes to older divergences of now allopatric ecotypes. Alternating shading denotes the different chromosomes; the horizontal black lines mark the mean *F*_ST_ and the horizontal red lines mark the 99^th^ percentile *F*_ST_. (**b**) Stacked bar plots show the proportion of SNPs in different genomic regions: exons, introns, 25-kb flanking and intergenic regions, in different *F*_ST_ bins, corresponding to the *y* axis of the Manhattan plots for each pairwise comparisons. Asterisks signify an over-representation of SNPs in a given region at each *F*_ST_ bin. Bar plots indicate the total number of SNPs in each *F*_ST_ bin for each pairwise comparison. (**c**) Correlations of 50-kb window-based estimates of differentiation (*F*_ST_) between all possible pairwise comparisons between ecotypes (above diagonal); correlations between 50-kb window-based estimates of genome-wide divergence (*D*_XY_), differentiation (*F*_ST_) and nucleotide diversity (*π*) for the pairwise comparison between the resident and transient ecotypes (below the diagonal); other comparisons are shown in Supplementary Fig. 13. Red indicates a positive relationship, blue a negative one; colour intensity and circle size are proportional to Spearman's correlation coefficient. Regions of high differentiation, but low diversity and divergence that are shared across pairwise comparisons, are likely to have been regions of selection on the ancestral form, which would remove linked neutral diversity and result in increased lineage-sorting of allele frequencies in these genomic regions in the derived forms.

Demographic bottlenecks during population splits and founding events, followed by subsequent demographic and geographic expansion, can produce rapid shifts in allele frequencies between populations^34^. The high levels of genome-wide differentiation (*F*_ST_) between killer whale ecotypes across all genomic regions (Fig. 4a,b) are consistent with strong genetic drift following demographic expansion from small founding groups. Considering the low efficiency of selection in populations as small as the estimates presented here^35^, in which founder populations have an estimated *N*e ranging from a few tens to hundreds, a genome-wide contribution of ecologically mediated divergent selection is neither necessary nor particularly likely to explain the observed shifts in allele frequencies in such a large number of loci. Consistent with this prediction, we find that differentiation is highest along the branches inferred by TreeMix to have experienced the most substantial genetic drift (Fig. 2a), that is, the branch to the ancestor of the Antarctic types and the branch to the resident ecotype (Supplementary Fig. 13). We therefore expect that only those beneficial alleles that have a strong favourable effect (that is, strength of selection (*s*)>1/2*Ne*) would have an increased fixation probability because of selection within these founder populations.

Much of the heterogeneity in the differentiation landscape was shared among pairwise comparisons (Fig. 4c). Since diversity (*π*) was not associated with mutation rate *μ*, as inferred from neutral substitution rate *d*S, (*r*=−0.1 to −0.24), the observed covariation in differentiation (*F*_ST_), diversity (*π*) and absolute divergence (*D*_xy_) between population pairs (Fig. 4c and Supplementary Fig. 14) is best explained by the shared local reduction of diversity by linked selection in the ancestral population^36^,^37^. This leads us to conclude that overall the landscape of genome-wide differentiation is a result of global genetic drift, regionally elevated by ancestral linked selection (for example, background selection or repeated selective sweeps shared among populations) independent of the evolutionary dynamics of the recently derived present-day ecotypes.

### Genomic signatures of climate and diet adaptation

However, against this background of shared differentiation, there was evidence for genic divergence of putative functional relevance. The first targets of selection following ecotype diversification and the exploitation of new ecological niches are expected to be those that facilitate ecological specialization^36^. Once a certain level of reproductive isolation is reached, differences can accumulate in other (fast-evolving) genes involved in reproductive isolation effectively reducing hybrid mating^38^. Following this rationale, both individual gene associations and gene ontology (GO) enrichment analyses yielded several biological processes and candidate genes with putative functional roles in ecological specialization, local adaptation and reproductive isolation (Supplementary Tables 6–8). For example, in comparisons between ecotypes inhabiting the extreme cold of the Antarctic pack ice with ecotypes from the more temperate North Pacific (Fig. 1), we found the most significant enrichment in genes involved in adipose tissue development (GO:0060612, Fisher's exact test: *P*=0.0015). Genes associated with adipose tissue development have previously been found to be evolving under positive selection in the polar bear^39^, suggesting a role for this process in rapid adaptation to a cold climate and lipid-rich diet.

Using the population branch statistic (PBS), which has strong power to detect recent natural selection^40^ and has allowed us to investigate allele changes along specific branches, we identified another candidate example where cold adaptation may play a role. The *FAM83H* gene showed a signature of selection (top 99.9% PBS values) and was found to contain four fixed non-synonymous substitutions derived in the Antarctic lineages based on the inferred ancestral state, which resulted in physicochemical changes including a hydrophobic side chain being replaced by a positively charged side chain. The keratin-associated protein encoded by the *FAM83H* gene is thought to be important for skin development and regulation through regulation of the filamentous state of keratin within cytoskeletal networks in epithelial cells, determining processes such as cell migration and polarization^41^. Skin regeneration is thought to be constrained in killer whales while inhabiting the cold waters around Antarctica because of the high cost of heat loss, and is thought to underlie rapid round-trip movements to warmer subtropical waters by Antarctic ecotypes^42^. The balance between skin regeneration and thermal regulation in Antarctic waters could be a major selective force requiring both behavioural^42^ and genomic adaptation (Supplementary Fig. 15).

Genes encoding proteins associated with dietary variation also showed a signature of selection (top 99.9% PBS values). For example, the *carboxylesterase 2* (*CES2*) gene encodes the major intestinal enzyme and has a role in fatty acyl and cholesterol ester metabolism in humans and other mammals^43^. Two exons of the *CES2* gene had among the top 99.9 percentile PBS values because of changes in allele frequencies (including two fixed non-synonymous amino-acid changes) along the branch to the Antarctic types (Supplementary Fig. 16). Similarly, genes in the top 99.9 percentile PBS values were enriched for carboxylic ester hydrolase activity (GO:0052689, Fisher's exact test: *P*<0.0001) in the fish-eating *resident* ecotype. Biological processes enriched in the resident killer whale included digestive tract morphogenesis (GO:0048546, Fisher's exact test: *P*=0.0022), and gastrulation with mouth forming second (GO:0001702, Fisher's exact test: *P*=0.0024): associated with the formation of the three primary germ layers of the digestive system during embryonic development. These results overlapped with enriched GO terms identified by a previously published RAD-seq study, despite the relatively sparse sampling of 3,281 SNPs in that study^19^. Enrichment of these GO terms was largely driven by differentiation in a single exon in the *GATA4* gene, which included a fixed non-synonymous substitution, sequenced in both studies.

**Figure 5:**
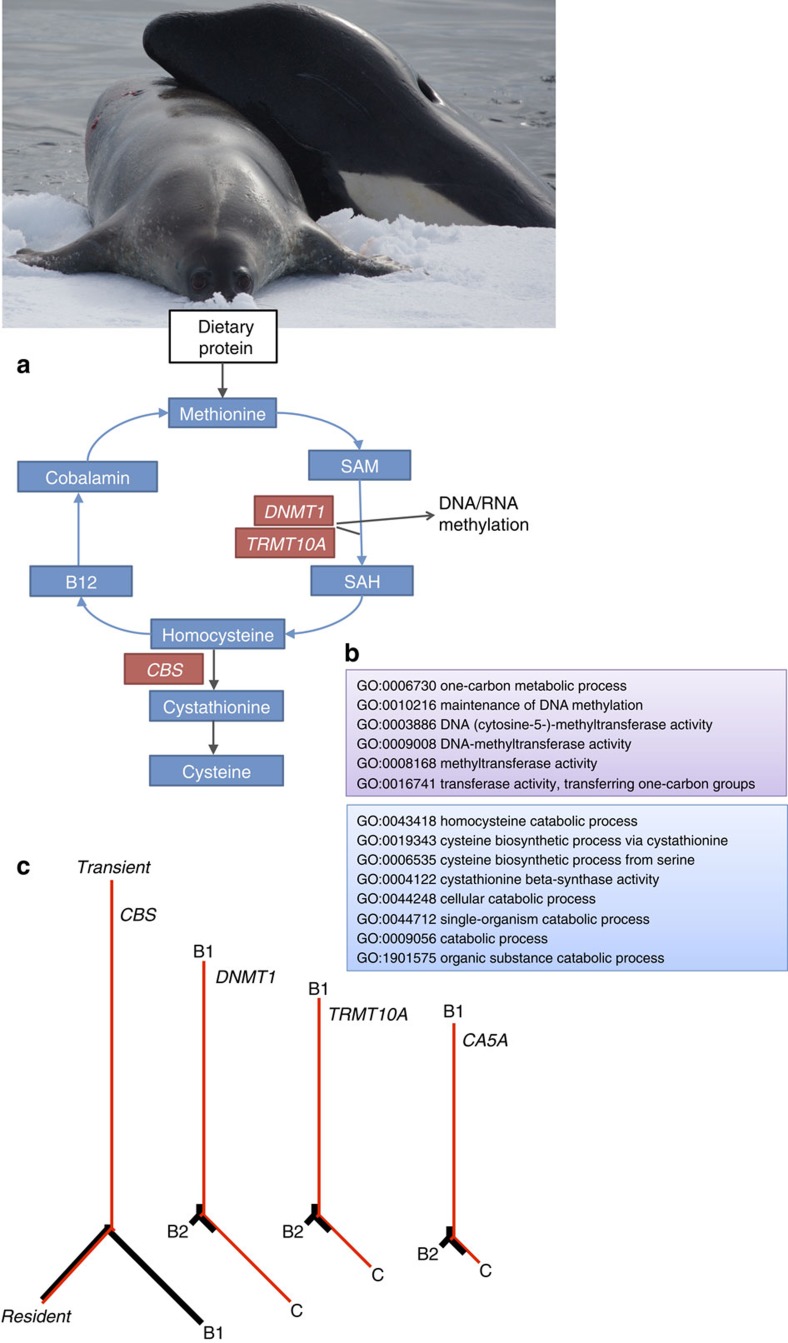
Signatures of selection in mammal-eating ecotypes in genes that play a key role in the methionine cycle. (**a**) Methionine is an essential amino acid that is obtained through dietary protein. The methionine cycle feeds into the folate cycle, and both are part of the complex and interacting network of pathways that encompass one-carbon metabolism^44^. Methionine adenyltransferase (MAT) then generates S-adenosylmethionine (SAM), which is then demethylated by the methyltransferases *DNMT1* (ref. 68) and *TRMT10A* (ref. 69) to methylate DNA and RNA, respectively, and to form S-adenosylhomocysteine (SAH)^68^. SAH is converted to homocysteine, which is then catalysed by cystathionine β-synthase, an enzyme encoded by the *CBS* gene to cystathionine, which is then converted to cysteine^68^. Any excess homocysteine is re-methylated to methionine. Adapted and simplified from KEGG pathway map 00270. Genes that play a role in this pathway and with a signature of selection (top 99.9% PBS values) in either of the predominantly mammal-eating ecotypes (the *transient* or *type B1*) are in red boxes. The gene encoding the carbonic anhydrase *CA5A*, which plays a role in the larger one-carbon pathway, also had a signature of selection (top 99.99 PBS value) in *type B1.* (**b**) Boxes indicate GO terms associated with the biological processes within the methionine cycle that were enriched in genes with the top 99.9 (*transients*; blue) and 99.99 (*type B1*; purple) percentile PBS values. (**c**) Evolutionary trees underlying the signal of selection. Population-specific allele frequency changes are indicated by the *F*_ST_-based (PBS values) branch lengths in these genes (red), which are overlaid on the genome-wide average branch lengths (black). The high (top 99.9–99.99%) PBS values indicate substantial changes in allele frequencies along the branches to the mammal-eating ecotypes. Differentiation in genes *DNMT1*, *TRMT10A* and *CA5A* was greatest between the mammal-eating *type B1* and the fish-eating *type C* among the Antarctic types. (Photo credit of ‘dietary protein': Robert Pitman, SWFSC.)

Signatures of selection along branches leading to the two predominantly mammal-eating ecotypes included in this study, the North Pacific *transient* and Antarctic *type B1*, were found in genes that play a key role in the methionine cycle (Fig. 5). Methionine is an essential amino acid that has to be obtained through dietary intake, and is converted through *trans*-sulfurcation to cysteine via intermediate steps of catalysis to homocysteine^44^. Any excess homocysteine is re-methylated to methionine^44^. Diets with different protein contents, such as between killer whale ecotypes, will differ in their content of methionine, and the enzymatic cofactors involved in the metabolism of methionine and homocysteine, which include folate, vitamins B6 and B12 (ref. 44; hence why vegetarians often take vitamin B12 supplements). While different genes and different biological processes showed a signature of selection in each of these two mammal-eating ecotypes (Fig. 5), in both cases the candidate genes and processes were associated with the regulation of methionine metabolism, which results in the generation of cysteine. Successful hunting of mammal prey by killer whales would provide a sudden and rich source of dietary methionine. This fluctuating intake of protein may place more of a selective pressure on the regulation of the metabolism of methionine than does the consumption of fish by piscivorous ecotypes.

### Rapid evolution of reproductive proteins

High (top 99.9 percentile) PBS values, largely driven by fixed non-synonymous amino-acid substitutions, were further estimated for several genes that encode proteins associated with reproductive function, including testis development, regulation of spermatogenesis, spermatocyte development and survival, and initiating the acrosome reaction of the sperm (for example, *PKDREJ*, *RXFP2*, *C9orf24*, *SPEF1*, *TSSK4, DHH* and *MMEL1*). Reproductive proteins such as *PKDREJ*, in which we found two fixed non-synonymous substitutions derived in the ‘resident' ecotype, are known to diverge rapidly across taxa, and because of their functional role in fertilization are emerging as candidates for the post-zygotic component of the speciation process^38^.

## Conclusions

Overall, our results indicate that the processes underlying genomic divergence among killer whale ecotypes reflect those described in humans in several respects. Behavioural adaptation has facilitated the colonization of novel habitats and ecological niches. Founder effects and rapid formation of reproductive isolation, followed by population expansion, have promoted genome-wide shifts in the frequency of alternative alleles in different ecotypes due to genetic drift. Demographic changes during founder events and subsequent expansions can also influence cultural diversity^45^,^46^, and may have had a role in reducing within-ecotype cultural diversity and promoting cultural differentiation between ecotypes. As with studies on modern humans, it is difficult to demonstrate a causal association between cultural differences and selection on specific genes^1^; however, our findings of divergence in genes with putative functional association with diet, climate and reproductive isolation broadly imply an interaction between genetically and culturally heritable evolutionary changes in killer whale ecotypes. Given these findings, the almost-exclusive focus on humans by studies of the interaction of culture and genes^5^ should be expanded, and exploration of culture–genome coevolution models in suitable non-human animal systems encouraged.

## Methods

### Sample collection

Skin biopsies from free-ranging killer whales were collected using projected biopsy darts, concurrent with the collection of photographic, photogrammetry and behavioural data, allowing sampled individuals to be binned to ecotype/morphotype *a priori* to genetic analyses. Most samples were selected from separate collection dates and identified groups (when known) to minimize chances of collecting close relatives or replicate individuals. The sample set included 10 ‘*resident*', 10 ‘*transient*', 7 *type B1*, 11 *type B2* and 10 *type C* killer whales.

### Genomic DNA library building and sequencing

DNA was extracted using a variety of common extraction methods as per ref. 11. Genomic DNA was then sheared to an average size of ∼150–200 bp using a Diagenode Bioruptor NGS. Illumina sequencing libraries were built on the sheared DNA extracts using NEBNext (Ipswich, MA, USA) DNA Sample Prep Master Mix Set 1 following Meyer and Kircher^47^. Libraries were subsequently index-amplified for 15 cycles using Phusion High-Fidelity Master Mix (Finnzymes) in 50-μl reactions following the manufacturer's guidelines. The libraries were then purified using the MinElute PCR purification kit (Qiagen, Hilden, Germany). The DNA concentration of the libraries was measured using a 2100 Bioanalyzer (Agilent Technologies, CA, USA); these were then equimolarly pooled by ecotype and each ecotype pool was sequenced across five lanes of an Illumina HiSeq 2000 platform using single-read 100-bp chemistry (that is, a total of 25 lanes).

### Read trimming and mapping

A high-quality, 2,249-Mb reference killer whale genome assembly (Oorca1.1, GenBank: ANOL00000000.2, contig N50 size of 70.3 kb, scaffold N50 size of 12.7 Mb)^16^ was used as a mapping reference. For the purpose of this study, the genome was masked for repetitive elements using RepeatMasker^48^ and the Cetartiodactyl repeat library from Repbase^49^. Repetitive elements constitute 41.32% of the killer whale reference genome (929,443,262 sites). A further 80,599 sites were identified as mitochondrial DNA transposed to the nuclear genome (numts) and were masked accordingly. The final assembly was then indexed using BWA v. 0.5.9 (ref. 50) to serve as the reference for read-mapping.

Illumina HiSeq 2000 reads from each individual were processed with AdapterRemoval^51^ to trim residual adapter sequence contamination and to remove adapter dimer sequences as well as low-quality stretches at 3′ ends. Filtered reads >30 bp were then mapped using Burrows-wheeler aligner (BWA), requiring a mapping quality greater than 30. Clonal reads were collapsed using the rmdup function of the SAMtools (v. 0.1.18) suite^52^. Ambiguously mapped reads were also filtered out using SAMtools. Consensus sequences were then reconstructed in Binary sequence/Alignment Map file format. To ensure that all repeat regions, which have the potential to bias population genetic inference, were removed, the per-site coverage was calculated across all 48 individuals. Inspection of the data suggested that sites with a total depth (including data from all 48 individuals) of >200 × reads were likely to be unmasked repeats. We therefore further masked these regions, which constituted an additional 0.14% of the genome (3,241,923 sites), resulting in a total masking of 932,685,185 sites (41.46% of the genome).

### Ancestral state reconstruction

The ancestral state for each site was inferred by mapping whole-genomic Illumina sequencing reads of the bottlenose dolphin (*Tursiops truncatus*, Short Read Archive accession code SRX200685)^16^ against the killer whale reference genome using BWA read mapper as above. The consensus sequence was called using SAMtools, and ambiguous bases were masked with N's. The ancestral state could be inferred for 2,206,055,540 (98.1%) of 2,249,565,739 bases.

### Inferring time to most recent common ancestor

Nine of the highest coverage killer whales were selected from our data set, which included two individuals each of the *resident*, *transient*, *type B2* and *type C* ecotypes, and a *type B1* individual. Estimates of the TMRCA were based on the number of derived transitions and number of derived transversions at third-codon positions. Exclusively considering mutations at third-codon sites was expected to minimize the impact of incomplete purifying selection, which can lead to overestimation of the substitution rate on short timescales. However, some mutations at third codons are non-synonymous, notably more so for transversions than for transitions, and putatively ephemeral transversions may therefore result in the overestimation of TMRCA. The lower rate of transversions, compared with transitions, is expected to minimize the impact of recurrent mutations at the same site, which could result in an underestimation of TMRCA based on transitions. Therefore, the expectation is that our estimate of TMRCA based on transversions may be upwardly biased, and our estimate based on transitions may be downwardly biased.

From a total of 4,781,830 third-codon positions (reduced to 3,127,876 when sites with missing data in any of the nine individuals were masked), 7,547 were inferred to be transversions and 12,784 were inferred to be transitions (with a minor allele frequency (MAF) cutoff of 0.1 so as to exclude potential sequencing errors) from the ancestral state (inferred from comparison with the dolphin genome). Of these, 7,120 transversions and 11,176 transitions were fixed in the killer whale, and therefore were inferred to have occurred along the branch from the killer whale/bottlenose dolphin ancestor and the MRCA of the killer whales (or because of incorrect inference of the ancestral state); moreover, 421 transversions and 1,608 transitions occurred within one or more of the killer whale lineages. A further six sites were inferred to have undergone a transversion from the ancestral state in at least one of the killer whale lineages, but had derived transitions in at least one other killer whale lineage. The Ts/Tv ratio of derived mutations at third-codon positions was therefore estimated to be 3.8 within the killer whale clade.

The proportion of derived mutations at third-codon positions found in one individual and shared in another individual is expected to decrease in comparisons between individuals from populations that diverged longer ago, as the probability that the mutation occurred within just one population following the split increases. The proportion of derived transversions and transitions at third-codon positions inferred within the *type B1* individual that were shared with each of the other eight individuals was measured. The two *resident* individuals shared the least number of derived transversions with the *type B1* individual (Supplementary Table 3). The results were highly consistent between individuals of the same ecotype (Supplementary Table 3). The mean rate of nucleotide evolution estimated for odontocetes of 9.10 × 10^−10^ substitutions per site per year (95% highest posterior density interval: 6.68 × 10^−10^, 1.18 × 10^−9^)^20^ was then scaled by our estimate of the Ti/Tv ratio of 3.8 at third-codon positions within our killer whale data set and was used to predict the time taken to accumulate 124 derived transversions and 465 transitions at third-codon positions that we inferred had been derived in *type B1* since splitting from a shared ancestor with the *resident* ecotype.

### Admixture analysis

An individual-based assignment test was performed to determine whether the ecotypes to which each individual had been assigned *a priori*, based on observed behaviour and/or morphological characteristics at the time of sampling, represented discrete gene pools in Hardy–Weinberg equilibrium. Since the 48 genomes generated for this study had an average sequencing depth of 2 × , genotypes can only be called with very high uncertainty. Therefore, NGSadmix^23^, a maximum likelihood method that bases its inference on genotype likelihoods (GLs) and in doing so takes into account the uncertainty in the called genotypes that is inherently present in low-depth sequencing data, was employed. The method has been demonstrated, using simulations and publicly available sequencing data, to have great accuracy even for very low-depth data of less than twofold mean depth^23^. GLs were estimated using the SAMtools method^52^ implemented in the software package ANGSD^53^. NGSadmix was run with the number of ancestral populations *K* set to 3–5. For each of these *K* values, NGSadmix was re-run multiple times with different seeds in order to ensure convergence. Sites were further filtered to include only autosomal regions covered in at least 40 individuals, and removing sites with a minor allele frequency below 5% estimated from the GLs, resulting in the analyses being based on 603,519 variant sites. The highest likelihood solutions can be seen in Fig. 2c.

### Principal Component Analysis

Assignment of individuals to ecotype, and structuring among ecotypes, was further investigated using Principal Component Analysis (PCA), implemented in the ngsTools suite^54^ taking genotype uncertainty into account^55^. Briefly, the covariance matrix between individuals, computed as proposed in ref. 56, is approximated by weighting each genotype for its posterior probability, the latter computed using ANGSD as described in ref. 18. The eigenvectors from the covariance matrix were generated with the *R* function ‘eigen', and significance was determined with a Tracy–Widom test to evaluate the statistical significance of each principal component identified by the PCA. The PCA was plotted using an in-house *R* script (available at https://github.com/mfumagalli/ ngsPopGen/ tree/master/scripts).

### Tests for ancestral admixture

The genetic relationships among ecotypes were further reconstructed in the form of a maximum likelihood graph representing the degree of genetic drift generated using TreeMix^24^. TreeMix estimates a bifurcating maximum likelihood tree using population allele frequency data and estimates genetic drift among populations using a Gaussian approximation. The branches of this tree represent the relationship between populations based on the majority of alleles. Migration edges are then fitted between populations that are a poor fit to the tree model, and in which the exchange of alleles is inferred. The addition of migration edges between branches is undertaken in stepwise iterations to maximize the likelihood, until no further increase in statistical significance is achieved. The directionality of gene flow along migration edges is inferred from asymmetries in a covariance matrix of allele frequencies relative to an ancestral population as implied from the maximum likelihood tree. We ran TreeMix fixing the *transient* ecotype as the root, with blocks of 1,000 SNPs (corresponding to approximately several hundred kilobases) to account for linkage disequilibrium among sites. The graphs are presented in Fig. 2a and Supplementary Fig. 5. This method does require genotype calls as an input, and is therefore susceptible to errors associated with genotype calling from low-coverage sequencing data. However, since TreeMix works on population allele frequencies, not genotypes, it was possible to determine the frequencies of the most common alleles with high confidence. The topology is comparable to output from other approaches applied here that do account for genotype uncertainty, providing confidence in the result.

### Estimating population genomic metrics

Measures of genetic differentiation, divergence and diversity were estimated using methods specifically designed for low-coverage sequencing data. Using a Maximum-Likelihood-based approach previously proposed^18^ and using the bottlenose dolphin genome to determine the ancestral state of each site, the unfolded SFS was estimated jointly for all individuals within a population for sites sequenced in five or more individuals in each population. Using this ML estimate of the SFS as a prior in an Empirical Bayes approach, the posterior probability of all possible allele frequencies at each site was computed^18^. For these quantities, the expectations of the number of variable sites and fixed differences between lineages were estimated as the sum across sites of the probability of each site to be variable as previously proposed^57^. Finally, the posterior expectation of the sample allele frequencies was calculated as the basis for further analysis of genetic variation within and between lineages.

*F*_ST_ was estimated with a method-of-moments estimator^58^ based on both the maximum likelihood estimate of the 2D-SFS^55^ and the sample allele frequency posterior probabilities of the 2D-SFS^55^. The two estimates were highly correlated (Pearson's correlation coefficient: *r*^2^>0.96) for all pairwise comparisons. However, from inspection of the data, the *F*_ST_ estimates generated from sample allele frequency posterior probabilities provided a more accurate estimation of fixed differences between populations. The likelihood-based method tends to flatten the *F*_ST_ peaks compared with the posterior probability method. This can result in masking of *F*_ST_ peaks with increasing genome-wide *F*_ST_ when using the likelihood-based method. Therefore, the posterior likelihood estimates are presented in the Manhattan plots of 50-kb sliding windows (Fig. 4a), with further filtering to only include windows for which >10 kb was covered by at least five individuals per population. We estimated the probability of a site being variable (Pvar). We tried different Pvar cutoffs and counted the number of variable sites at each Pvar. We then decided on Pvar=1, as the number of variable sites matched our expectations from estimates of diversity (*π*) from the two high-coverage genomes. We further checked by comparing *F*_ST_ estimates from a recently published whole-genome data set of carrion crows (*Corvis corone*) and hooded crows (*C. cornix*)^59^ downsampled to low coverage. By only considering sites with a Pvar of 1, we obtained *F*_ST_ estimates comparable to the values obtained from the 7–28 × sequences using GATK. Population-specific allele frequencies were estimated with the ancestral state fixed and the derived allele weighted by the probabilities of the three possible states. The allele frequencies were estimated based on GLs. To assess the robustness of our per-site *F*_ST_ value estimates, we evaluated how well the values correlated with those estimated from RAD-seq data generated in a previous study^19^. We accessed the SNP data in a VCF file format generated by the RAD-seq study from Dryad (doi:10.5061/dryad.qk22t) and used VCFtools to calculate per-site *F*_ST_, using sites called at >20 × coverage, between 43 individuals of the resident ecotype and 37 individuals of the transient ecotype (Supplementary Fig. 2a). We then performed 1,000 replicates, randomly subsampling with replacement of 10 individuals from each ecotype. The correlation between *F*_ST_ estimates from these random subsamples ranged from 0.6861 to 0.9372. We found the correlations between estimates of *F*_ST_ from the RAD-seq data and those from our whole genome sequencing (WGS) data ranged from 0.5475 to 0.7140 (Supplementary Fig. 2b). The significant correlation in estimates of *F*_ST_ between two different methods using different individuals suggests that these estimates are reliable.

The average number of nucleotide substitutions *D*xy was then calculated as the mean of p1*q2+p2*q1, where p1 is the allele frequency of allele 1 in population 1 and p2 in population 2, q1 is the allele frequency of allele 2 in population 1 and q2 in population 2. Sites that were not variable in any of the populations were assigned allele frequencies of zero.

Applying the probabilistic model implemented in ANGSD, we estimated the unfolded SFS in steps of 50-, 100- and 200-kb windows using default parameters and GLs based on the SAMtools error model. From the SFS we derived nucleotide diversity *π* (Supplementary Table 9). Estimates of nucleotide diversity can be influenced by differences in sequencing coverage and sequencing error. However, it has been shown that, using an empirical Bayes approach, implemented in ANGSD, the uncertainties in low-depth data can be overcome to obtain estimates that are similar to those obtained from data sets in which the genotypes are known with certainty^60^. Multiple checks were performed to ensure that estimates of *π* were not an artefact of the data-filtering. Comparable estimates of *π* were obtained using the method implemented in ANGSD for a single 20 × coverage ‘resident' genome (*π*=0.0009) when it was randomly downsampled to 2 × coverage (*π*=0.0008). Nucleotide diversity was estimated for sites covered by at least five individuals in each population in windows of size 50, 100 and 200 kb (Supplementary Table 9).

### Demographic reconstruction

PSMC analysis^21^ was performed on the high-coverage diploid autosomal genome sequences of two individuals to investigate changes in effective population (*N*_e_) size. The PSMC model estimates the TMRCA of segmental blocks of the genome and uses information from the rates of the coalescent events to infer *N*_e_ at a given time, thereby providing a direct estimate of the past demographic changes of a population. The method has been validated by its successful reconstructions of demographic histories using simulated data and genome sequences from modern human populations^21^.

A more detailed account of PSMC analyses, including our reanalyses and reinterpretation of a previously published analysis of low-medium coverage genomes^17^, is presented in the Supplementary Figures and Notes. A PSMC plot of the Atlantic genome subsampled to 20 × and a North Pacific *resident* genome (20 × ) was estimated, scaled to the autosomal mutation rate of 2.34 × 10^−8^ substitutions per nucleotide per generation^20^ and is presented in Fig. 3a. A PSMC plot of the autosomal regions of the North Atlantic female killer whale at a coverage of 50 × was scaled to the autosomal mutation rate (*μ*_A_) of 2.34 × 10^−8^ substitutions per nucleotide per generation^20^, as used in our estimation of TMRCA (see above) and was compared with a plot of the diploid X-chromosome, scaled to real time as per ref. 21 in which the neutral mutation rate of the X-chromosome was derived as *μ*X=*μ*A[2(2+*α*)]/[3(1+*α*)], assuming a ratio of male-to-female mutation rate of *α*=2 (ref. 61). This gave us an estimated *μ*X=2.08 × 10^−8^ substitutions per nucleotide per generation. The plot is presented in Fig. 3b. We also re-estimated the PSMC plot for the X-chromosome using different mutation rates to investigate which rate would produce PSMC plots with inferred concurrent declines in *N*_e_ in autosomes and X-chromosome. We found that *μ*X=1.00 × 10^−8^ substitutions per nucleotide per generation would be needed to synchronize the inferred demographic changes in these two markers (Supplementary Fig. 17). This would require the male-to-female mutation rate (*α*) to be orders of magnitude higher, making it seemingly biologically unrealistic.

To reconstruct ancestral demography in the ecotypes for which we did not have a high-coverage genome, we applied a method that uses the SFS from population genomic data to infer ancestral population size changes^33^. The Stairway Plot method first uses SFS from population genomic sequence data to estimate a series of population mutation rates (that is, *θ*=4*N*_e_*μ*, where *μ* is the mutation rate per generation and *N*_e_ is the effective population size), assuming a flexible multi-epoch demographic model. Changes in effective population size through time are then estimated based on the estimations of *θ*. As input data, we transformed the probability estimates of our site frequency spectra into SNP counts. We first ran the method on the resident ecotype to compare with the demographic reconstruction suggested by the PSMC analysis on the high-coverage North Pacific individual (see above). Population structure is a notable confounding factor for inferring demographic history^30^,^62^. Consistent with this, we estimate broad confidence intervals of our estimates of *N*_e_ subsequent to the estimated bottlenecks in each ecotype, during a period overlapping with previous estimates of within-ecotype lineage splits^22^. As we have sampled individuals from multiple subpopulations of the *resident* and *transient* (and possibly the Antarctic types, although less in known about population structuring in Antarctic waters), we potentially skew the SFS towards low-frequency polymorphism, thereby mimicking the pattern generated by population expansion^63^. We therefore opted not to include SNP counts for singletons and doubletons, which are expected to have arisen recently within each ecotype and had no time to be shared throughout the population, as these may be biased by our sampling protocol and low-coverage sequencing data, and as our interest was in demographic change during population splits, which were estimated to have occurred over 10,000 years ago. Our focus is on the timing and extent of the bottlenecks within each ecotype, which all individuals within an ecotype coalesce back to and therefore pre-date within-ecotype population splits and substructuring and the emergence of derived singleton and doubletons.

The inference of demographic history from population genomic data by the Stairway Plot method provided a somewhat comparable result to reconstruction from a single 20 × genome by PSMC with respect to the time of onset and magnitude of a demographic decline inferred by both methods. The Stairway Plot method inferred a sudden drop in *N*e, whereas a more gradual decline was inferred by PSMC, consistent with simulations showing that PSMC can infer abrupt changes in *N*e as gradual changes^21^. The sudden change in estimated effective population size in the stairway plot is because of the method being based on a multi-epoch method, in which epochs coincide with coalescent events^33^. Therefore, the plot is not continuous, but rather it depicts discrete blocks of time (epochs). The number of epochs is determined by the number of individuals within each sample and the number of SNP bins used, that is, the number of possible coalescent events. The Stairway Plot method inferred a subsequent and rapid expansion, whereas PSMC did not infer an expansion within our cutoff point of 10,000 years ago, but did infer a more gradual expansion during the Holocene. It should be kept in mind that the PSMC plot is based on data from a single individual and so will track declines in *N*e because of further founder effects as the resident ecotype continues to split into multiple discrete populations. In contrast, the Stairway Plot is based on population genomic data and will track the change in Ne across all the sampled resident populations after they have split.

The method was then applied to the site frequency spectra of the transient ecotype and *type C*. The results are shown in Fig. 3c, and in each case the Stairway Plot infers a sudden and dramatic demographic decline, consistent with previously inferred population split times followed by a demographic expansion. The timing of the decline of the Antarctic types overlapped because of the recent shared ancestry, and therefore only the plot for *type C* is shown in Fig. 3c for clarity.

### Inferring putatively functional allele shifts because of selection

Shifts in allele frequencies can occur because of selection, but differences in allele frequencies can also accumulate between populations because of drift^34^,^35^. To infer shifts in allele frequencies potentially due to selection, we considered the 1% of SNPs with the highest *F*_ST_ values from each pairwise comparison between killer whale ecotypes. However, as *F*_ST_ is dependent on the underlying diversity of the locus, even extreme outlier loci can be due to genetic drift alone. We, therefore, additionally looked for over-representation of the top 1% outliers in different categories, for example, exons, 25-kb flanking regions (potential regulatory regions), introns and intergenic regions, at different *F*_ST_ bins using a *χ*^2^-test. Residuals are expected to be normally distributed and indicate statistical significance of over- and under-representation of specific categories. The significance threshold was subjected to Bonferroni correction. The top 100 outliers in exons from each pairwise comparison were then used for GO over-representation analysis^64^ to identify enrichment due to diet (in mammal- versus fish-eating ecotypes) and climate (in Antarctic versus Pacific).

To more robustly infer whether genetic changes in exons were associated with ecotype divergence due to selection, we applied the PBS^40^. The PBS has strong power to detect (even incomplete) selective sweeps over short divergence times^5^,^40^,^65^, making it relevant for the scenario we are investigating in this study. We therefore estimated the PBS for 50-kb sliding windows shifting in 10-kb increments (approximating to a window size of 10 SNPs) to detect regions of high differentiation potentially due to selective sweeps. We followed the approach of Yi *et al*.^40^, and used the classical transformation by Cavalli-Sforza^66^, *T*=−log(1−*F*_ST_) to obtain estimates of the population divergence time *T* in units scaled by the population size. For each 50-kb window, we calculated this value between each pair of ecotypes. The length of the branch leading to the one ecotype since the divergence from a recent ancestor (for example, in the equation given the length of the branch to *type B1* since diverging from types B2 and C) is then obtained as


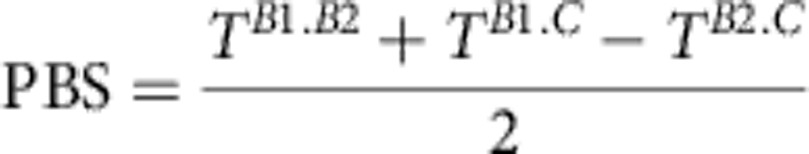


These window-based PBS values represent the amount of allele frequency change at a given 50-kb genomic region in the history of this ecotype (since its divergence from the other two populations)^40^. To further narrow down the target of selection, the PBS was estimated for exons as per ref. 5 and compared with genome-wide values to identify whether they were in the top 99.9 percentile (for the branches to the *resident*, *transient* and ancestral *Antarctic* ecotypes) and the top 99.99 percentile for the shorter branches leading to each Antarctic ecotype from their most recent common shared ancestor. We further filtered outliers to just include exons that encoded non-synonymous amino-acid substitutions and searched the database GeneCards^67^ for functions of the encoded proteins to identify potential targets of natural selection due to ecological differences. Supplementary Table 10 contains a list of outlier loci and the corresponding PBS value for each branch. Supplementary Data 2 contain details of fixed non-synonymous changes in the exons of protein-coding genes, including the per-individual and per-population read counts for each allele.

## Additional information

**Accession codes:** All sequencing data are available in European Nucleotide Archive (ENA) under accession numbers ERS554424– ERS554471 (see Supplementary Table 1 for a full list of accession numbers and associated sample ID).

**How to cite this article:** Foote, A. D. *et al*. Genome-culture coevolution promotes rapid divergence of killer whale ecotypes. *Nat. Commun.* 7:11693 doi: 10.1038/ncomms11693 (2016).

## Supplementary Material

Supplementary Information Supplementary Figures 1-17, Supplementary Tables 1-10, Supplementary Note 1 and Supplementary References

Supplementary Data 1 D-statistic (ABBA-BABA test) values and standard error estimates for the inference of ancient admixture.

Supplementary Data 2 Details of fixed non-synonymous changes in the exons of protein coding genes, including the per-individual and per-population read count for each allele.

Supplementary Data 3 Pairwise genetic distance estimates and 100 bootstrap replicates.

Supplementary Data 4 Pairwise relatedness estimates
