# Supplementary material for: Genome-culture coevolution promotes rapid divergence of killer whale ecotypes
Source: Nat Commun. 2016 May 31;7:11693. doi: 10.1038/ncomms11693 (PMC4895049; doi:10.1038/ncomms11693)
Supplement: Supplementary Information — Supplementary Figures 1-17, Supplementary Tables 1-10, Supplementary Note 1 and Supplementary References [file ncomms11693-s1.pdf]

Supplementary Figures

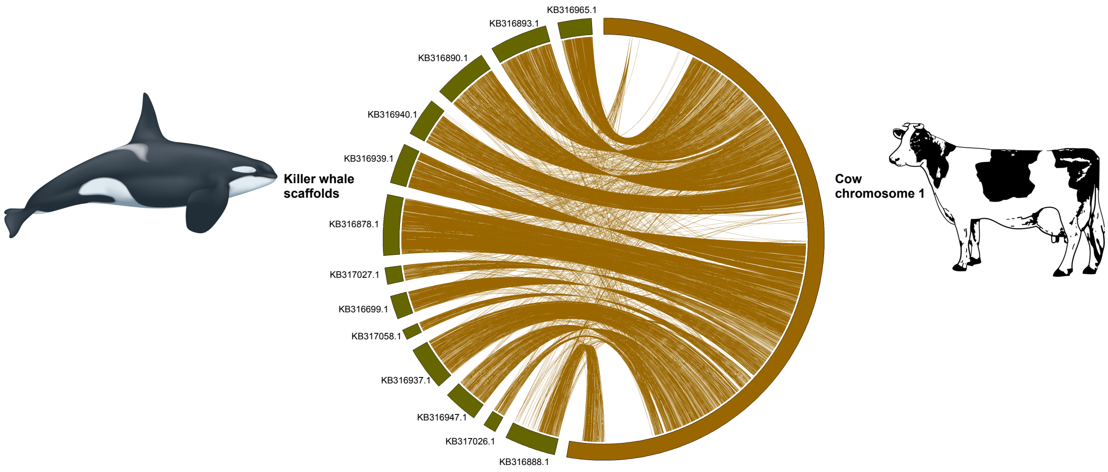

Supplementary Figure 1. Circos plot of chromosome 1 of the cow genome (brown) and corresponding scaffolds in the killer whale genome (dark green). For illustrative purposes (e.g. Manhattan plots) a synteny based chromosomal assembly of the reference genome was produced by aligning the killer whale scaffolds to a chromosomal assembly of the cow *Bos taurus* (Btau_4.6.1) genome using the *Satsuma* aligner[70] with default settings. Synteny was conserved and showed no large-scale inter- nor intra-chromosomal rearrangements in any scaffolds. Inferences based on outlier peaks were not influenced by this super- scaffolding process.

a

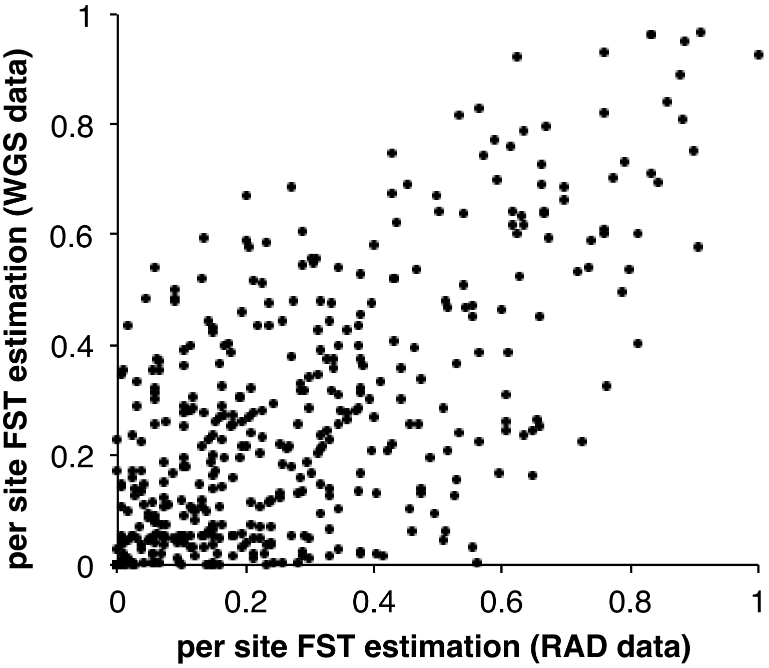

b

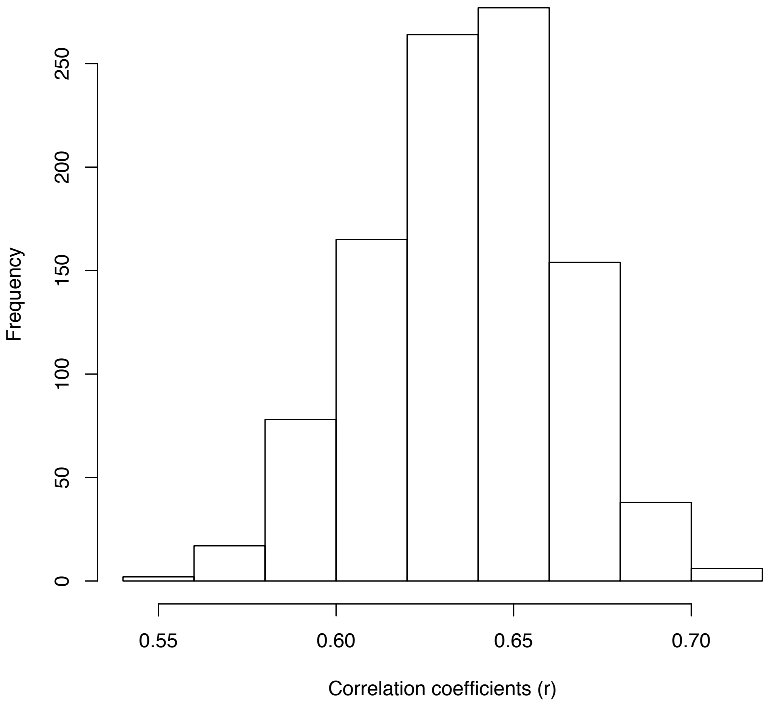

Supplementary Figure 2. Correlation of per-site F_{ST} between low-coverage whole genome sequencing (WGS) data generated for this study and high coverage (>20×) published RAD-seq data. **a,** Per-site F_{ST} estimates from a pairwise comparison of WGS data of 10 *residents* and 10 *transients* plotted against F_{ST} estimates of the same 547 polymorphic sites from a pairwise comparison from RAD data of 52 *residents* and 37 *transients*[19]. **b,** Distribution of the correlation coefficients (*r*) of the per-site F_{ST} estimates from a pairwise comparison of WGS data of 10 *residents* and 10 *transients* with 1,000 random re-samplings with replacement of 10 *residents* and 10 *transients* from the RAD-seq dataset[19].

a

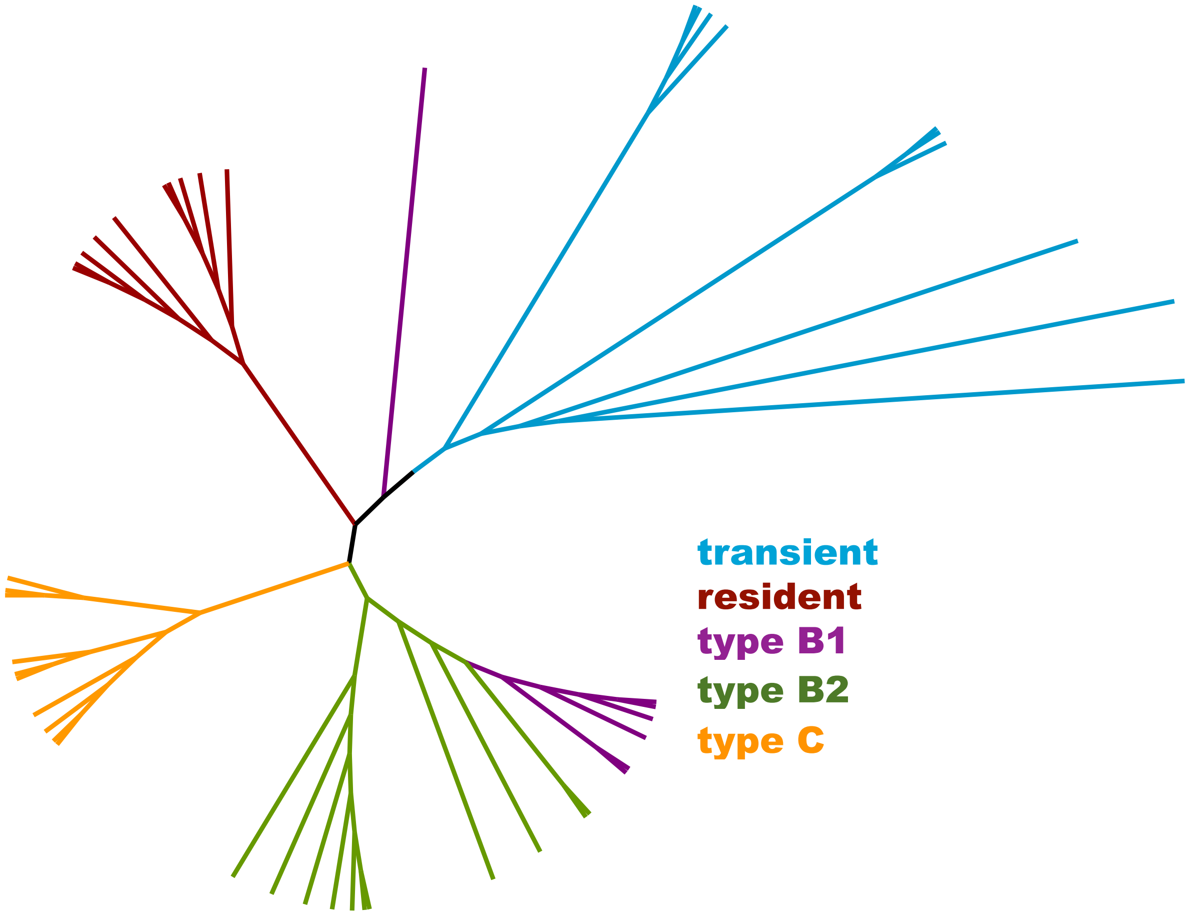

Supplementary Figure 3a, Maximum-likelihood phylogenetic tree reconstructed from mitochondrial genome sequences generated as per reference 72. Filtered reads were further mapped to a reference mitochondrial genome (GU187176.1) and compared with previously published mitogenome sequences from these individuals[11]. The assembled mitogenome sequences were a 100% match with those previously generated for these individuals using targeted sequencing approaches[11]. As previously reported, the mitogenomes of each ecotype clustered in strongly supported mitochondrial DNA clades, with the exception that one *type B1* individual sampled at a different geographic location to the other *type B1* individuals, had a highly divergent mitogenome haplotype[11].

b

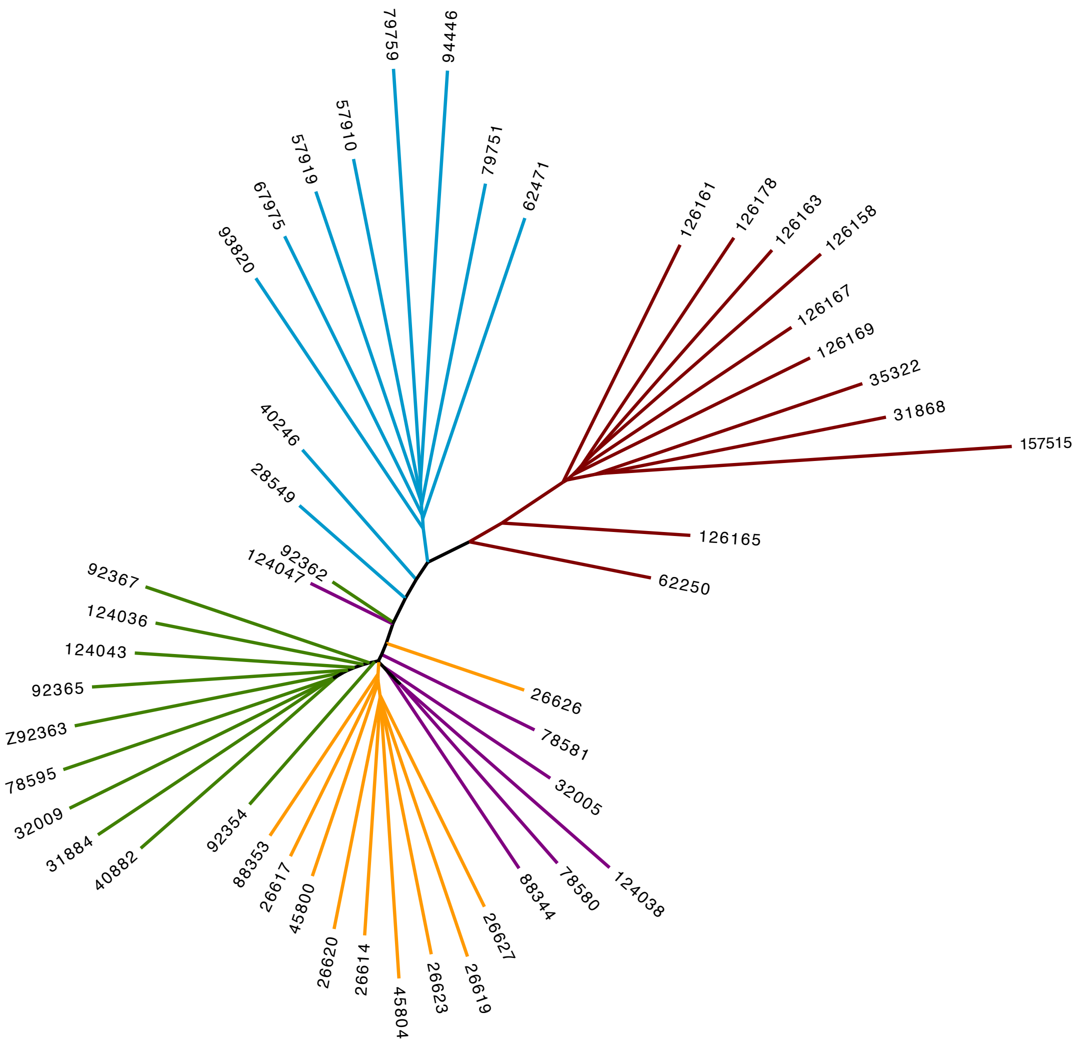

Supplementary Figure 3b, Distance-based tree generated from the 48 low coverage and one high (~20×) coverage nuclear genome sequences. We generated 100 matrices of pairwise genetic distances, in which pairwise genetics distances were calculated using ngsDist[72], which takes genotype uncertainty into account by avoiding genotype calling and instead using genotype posterior probabilities estimated by ANGSD. A block-bootstrapping procedure was used to generate 100 distance matrices, obtained by repetitively sampling blocks of the original data set (Supplementary Data 3). Pairwise genetic distances were visualised as a phylogenetic tree using the distance-based phylogeny inference program FastME 2.0[73]. Individuals largely clustered by ecotype indicating that segregating alleles are shared among individuals within each ecotype. The short branches that did not cluster as closely to ecotype were the individuals with the least sites covered at ≥2× (see Supplementary Table 1).

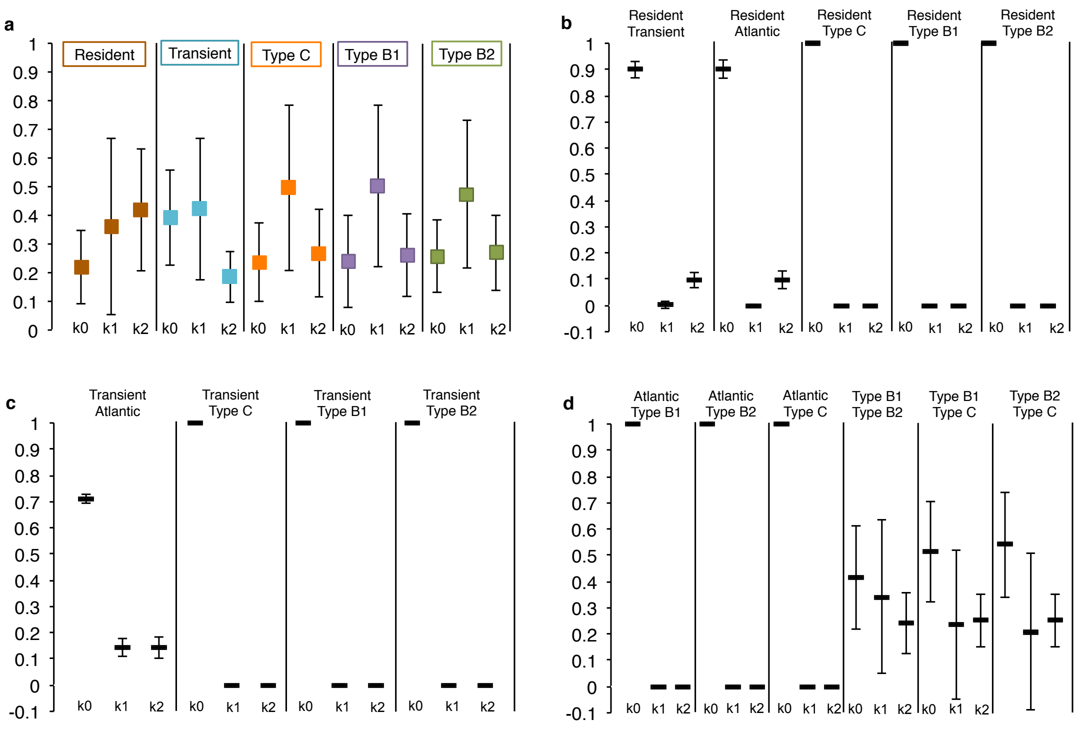

Supplementary Figure 4. Maximum likelihood estimates of pairwise relatedness based on genotype likelihoods. We estimated pairwise relatedness due to identity- by-descent (IBD), i.e. genetic identity due to a recent common ancestor, of every possible combination of two individuals using NgsRelate[74]. NgsRelate provides ML estimates of R, where R = ($k0$,$k1$,$k2$) and km is the fraction of genome in which the two individuals share m alleles IBD. NgsRelate provides maximum likelihood estimates of R by finding the value of R that maximizes this likelihood function with an Expectation Maximization algorithm using genotype likelihoods instead of genotypes to account for the inherent uncertainty of the genotypes. NgsRelate has been shown using simulations and real data to provide robust estimates for low-depth NGS data (as low as 1-2×), which are markedly better than genotype-based methods[74]. Each plot shows the proportion of the genome for k_m in which two individuals share m alleles IBD in pairwise comparisons **a,** among individuals from the same ecotype, **b,** between an individual from the *resident* ecotype and an individual from another ecotype, **c,** between an individual from the *transient* ecotype and an individual from another ecotype**, d,** among individuals from the different Antarctic ecotypes, and a North Atlantic individual. The full results are reported in Supplementary Data 4.

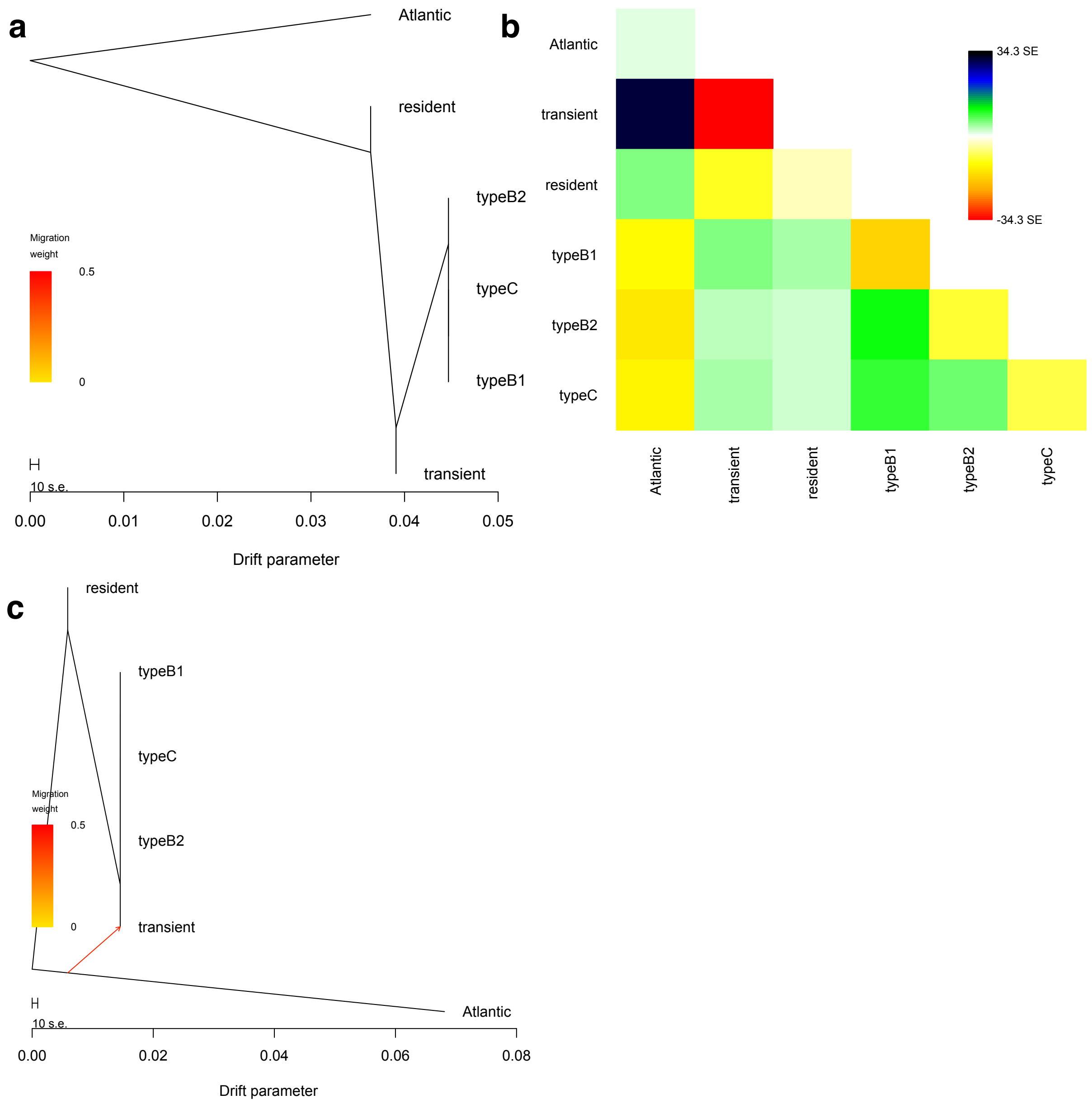

Supplementary Figure 5. a, Maximum-likelihood graph from TreeMix. The scale bar shows ten times the average standard error of the entries in the sample covariance matrix. **b,** Residual fit of the observed versus predicted squared allele frequency difference, expressed as the number of standard errors of the deviation. Colours are in the palette on the right. Residuals above zero represent populations that are more closely related to each other in the data than in the best-fit tree, and are candidates for admixture. **c,** Maximum-likelihood graph allowing a migration event to improve the fit of the tree to the data.

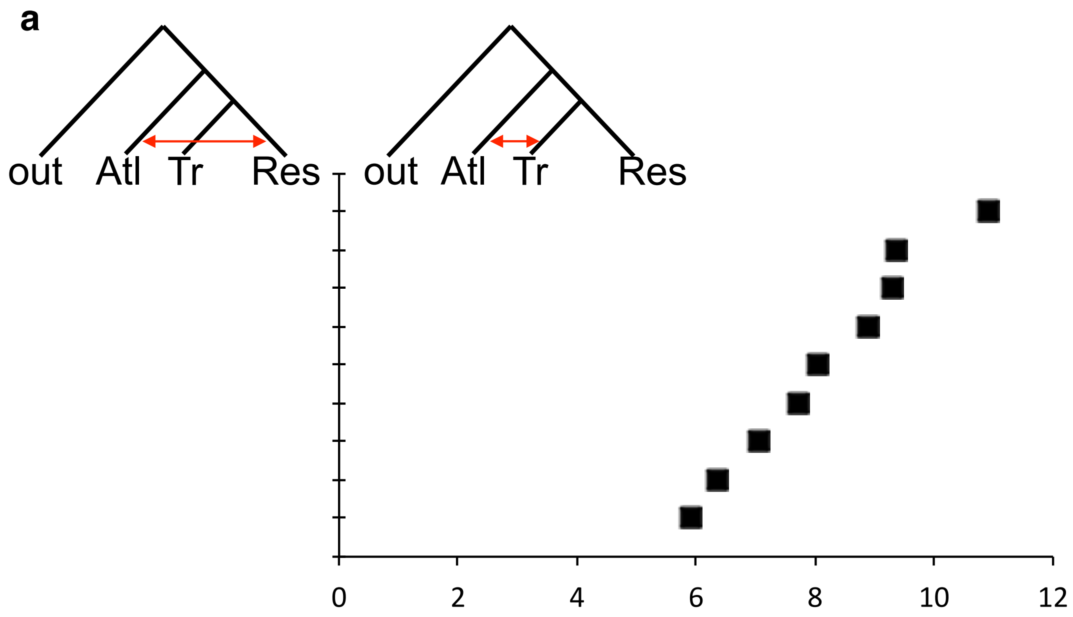

Supplementary Figure 6a. Plots of the Z-scores of D-statistic (ABBA-BABA) tests. D-statistic tests were performed on 9 comparisons of combinations of 3 *transients*, 3 *residents* and the Atlantic sample, with the bottlenose dolphin as the outgroup. This statistic identifies an excess of shared derived alleles between taxa, which could result from introgression or ancestral population structure. The statistic can thus be used to identify departures from 'treeness' of a given topology. For example, if H1, H2 and H3 are taken to denote 3 ecotypes, the test can be used to evaluate if the data are inconsistent with the null hypothesis that the tree (((H1, H2), H3), dolphin) is correct and that there has been no gene flow between H3 and either H1 or H2 or any populations related to them. The definition used here is from reference 75:

$$D = (n\text{ABBA}-n\text{BABA}) / (n\text{ABBA}+n\text{BABA})$$

where *n*ABBA is the number of sites where H1 shares the ancestral allele with the dolphin, and H2 and H3 share a derived allele (ABBA sites); and, *n*BABA is the number of sites where H2 shares the ancestral allele with the dolphin, and H1 and H3 share a derived allele (BABA sites). Under the null hypothesis that the given topology is the true topology, we expect an equal proportion of ABBA and BABA sites and thus D = 0. Hence a test statistic that differs significantly from 0 provides evidence either of gene flow or the tree being incorrect due to ancestral population structuring. The significance of the deviation from 0 was assessed using a *Z*-score based on jackknife estimates of the standard deviation of the D-statistics. This *Z*-score is based on the assumption that the D-statistic (under the null hypothesis) is normally distributed with mean 0 and a standard deviation equal to a standard deviation estimate achieved using the "delete-m Jackknife for unequal m" procedure. The tests were implemented in ANGSD and performed by sampling a single base at each position of the genome to remove bias caused by differences in sequencing depth.

The positive values over the critical value of 3 indicate an excess of ABBA patterns over BABA patterns in terms of the number of shared derived alleles. This indicates that the relationship among these taxa is not fully described by a bifurcating tree model, but that instead ancient admixture occurred between the ancestral populations of the North Pacific *transient* samples and the North Atlantic samples included here.

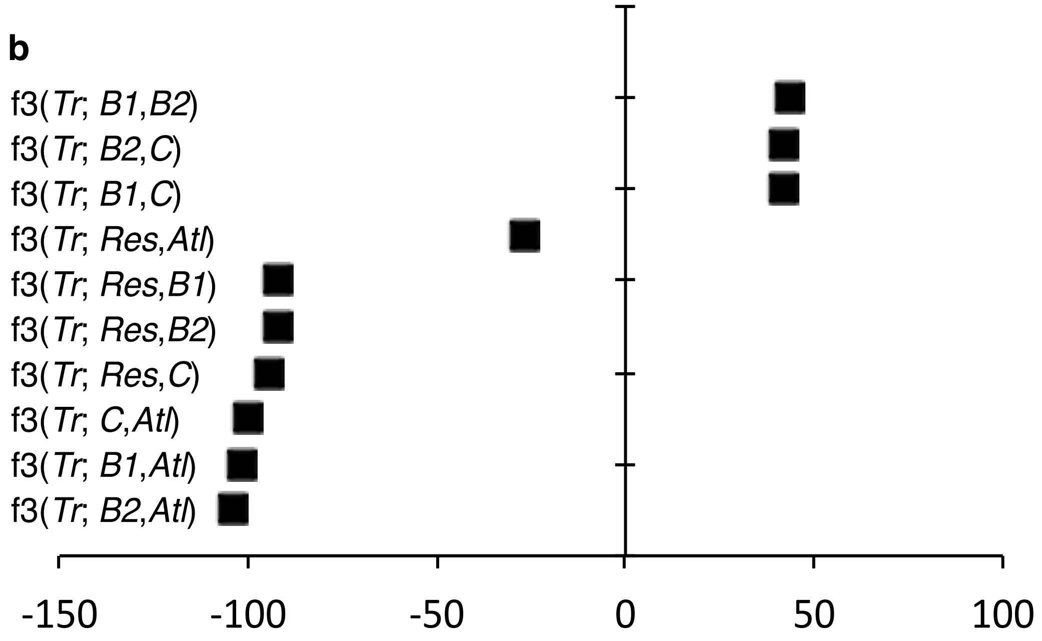

Supplementary Figure 6b. Plots of the Z-scores of three-population tests.
The three-population (*f*3) test, which can provide evidence of admixture, even if gene flow events occurred hundreds of generations ago[26], was implemented in TreeMix to test for 'treeness', i.e. how well relationships can be represented by bifurcations. These tests are of the form *f*3(A;B,C), where a significantly negative value of the *f*3 statistic implies that population A is admixed[26]. *f*3-statistics were computed using the estimators described in reference 26, obtaining standard errors using a block jackknife procedure over blocks of 1,000 SNPs. We find strongly negative Z-scores in the three-population test in comparisons where the *transient* ecotype is the target population and the Atlantic and/or *residents* are the source population, but not the other way around. This implies directional ancient introgression from the populations related to the *resident* and the Atlantic populations into the *transient* ecotype.

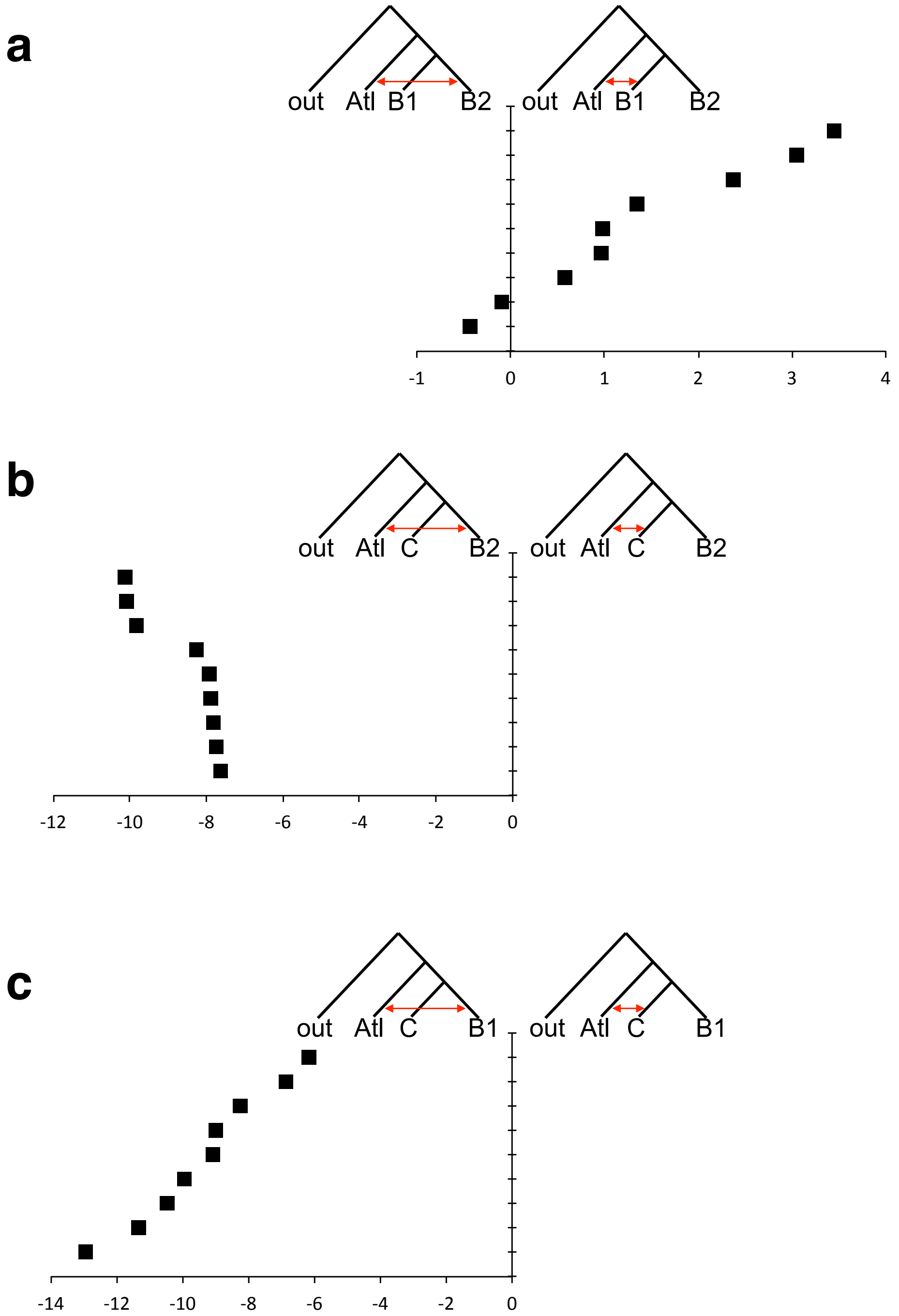

Supplementary Figure 7. Plots of the Z-scores from D-statistic tests performed on nine comparisons of combinations of **a,** three *type B1*, three *type B2* and the Atlantic sample; **b,** three *type C*, three *type B2* and the Atlantic sample; **c,** three *type C*, three *type B1* and the Atlantic sample; with the bottlenose dolphin as an outgroup in each comparison. In figure **a,** Z-scores are almost all below the critical value of ±3 indicating that *type B1* and *type B2* each share a roughly equal amount of derived alleles with the Atlantic population, i.e. that there was no subsequent admixture between the Atlantic population and either *type B1* or *type B2* after *types B1* and *B2* diverged. In figures **b,** and **c,** Z-scores are strongly negative and indicate an excess of BABA patterns over ABBA patterns and that *types B1* and *B2* terms shared more derived alleles with the Atlantic population than *type C* does. This implies ancient admixture between the Atlantic population and the ancestral population of *types B1* and *B2* after they diverged from *type C*.

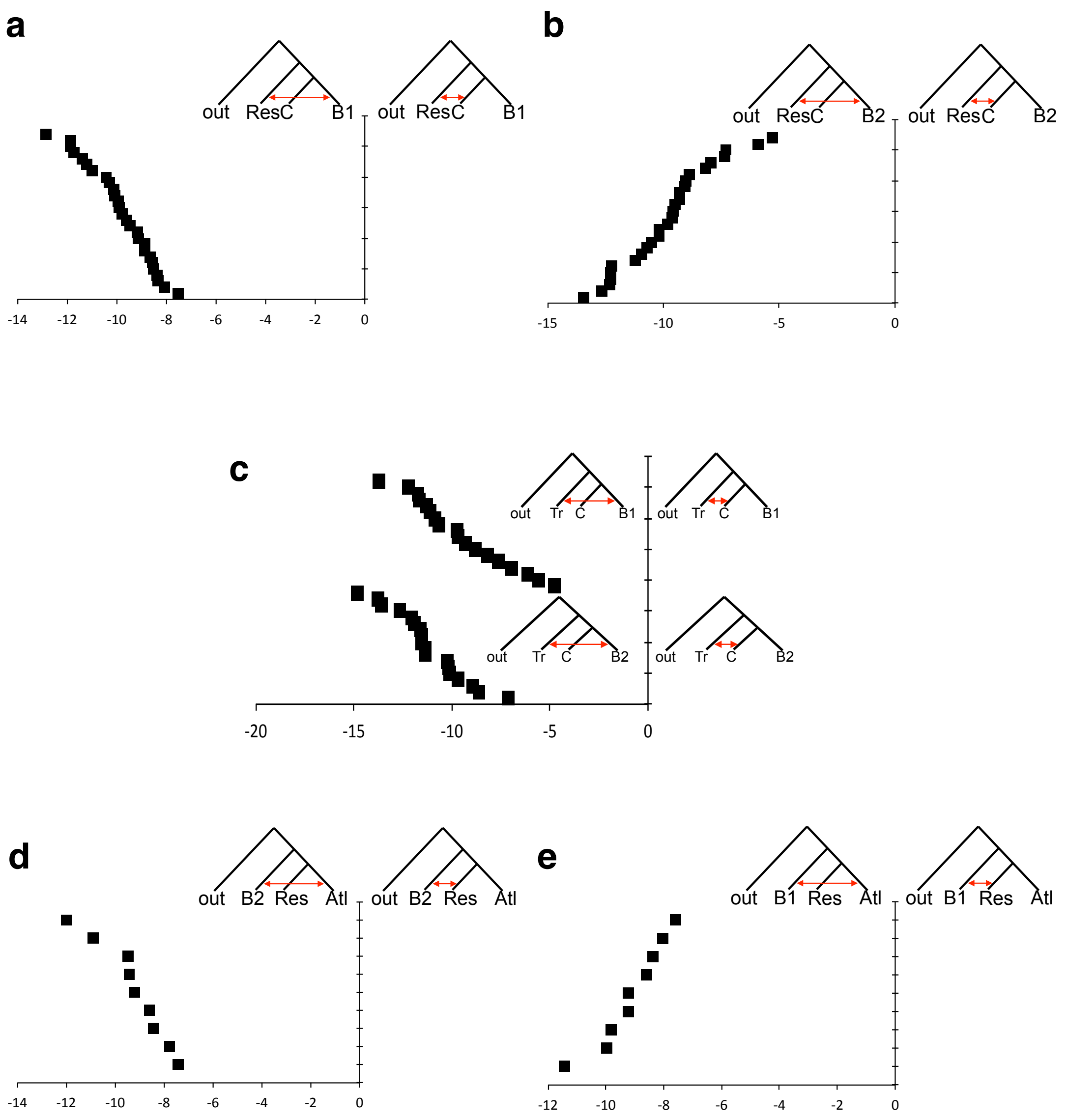

Supplementary Figure 8. Plots of the Z-scores from D-statistic tests. **a-c,** indicate that the Antarctic *types B1* and *B2* also share an excess of alleles with the other Northern hemisphere ecotypes relative to *type C*. This suggests that the ancient admixture event was likely between the ancestral *type B* population and a population closely related to the Atlantic, *resident* and *transient* populations, rather than multiple admixture events with each of those populations. **d,** and **e,** indicate that this admixture occurred after the *resident* and Atlantic populations split. Further results, D-statistic values and standard error estimates from all comparisons of all combinations can be found in Supplementary Data 1.

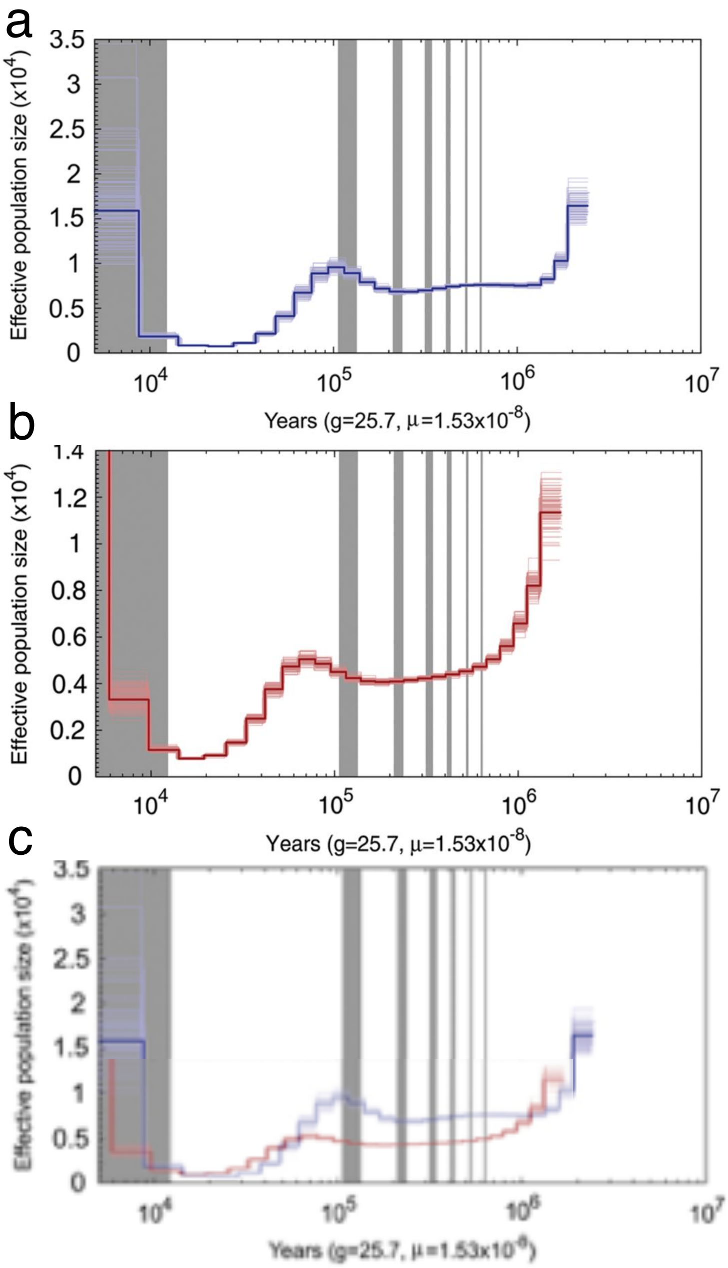

Supplementary Figure 9. Reassessment of a published PSMC demographic reconstruction. Previously published plots of demographic history inferred by pairwise sequential Markovian coalescent analysis (PSMC) of **(a)** a North Pacific resident killer whale and **(b)** a North Atlantic killer whale from figure 1 of Moura *et al*.[17] reproduced with permission from the publisher Oxford University Press (Licence Number: 3786451154395). Overlaying the two plots, **(c)** we observe that the two genomes show no convergence in effective population size (N_e) prior to the date that they are estimated by the same authors to have shared a common ancestor[22] or even back as far as 1 MYA. This highlights the bias introduced into the analysis when comparing sequences of low and differing coverage, which results in different rates of false negative detection of heterozygote sites, producing the same effect as using a lower mutation rate for the sequence with lower coverage.

A recent study used PSMC to reconstruct ancestral changes in *N*e through time using ~13× and ~20× coverage diploid autosomal genome sequences of a North Atlantic

killer whale (accessed ahead of publication by the consortium that generated the data) and a North Pacific killer whale respectively[17]. The authors interpreted the plots as being indicative of a global decline driven primarily by climate change during the last glacial period of the Pleistocene[17]. This study dismissed changes in connectivity having a role in the observed demographic changes as being 'unlikely to generate the specific pattern observed (strong population decline) or the very similar profiles for each ocean[17]. However, PSMC heavily relies on the distribution of polymorphic sites across the genome, and in particular, the length of shared runs of homozygosity, and can be biased when heterozygous sites are wrongly called as being homozygous. PSMC plots of genomes with <20× coverage have been shown not to be directly comparable to higher coverage genomes without first applying a correction for an appropriate false negative rate of detecting heterozygotes[76]. Additionally, the effect of mapping short read data to a reference genome comprised of short contigs may also be problematic, especially when many contigs fall below ~50-kb (the typical size of shared fragment size from 1,000 generations ago in humans), although to the best of our knowledge, the effect of mapping to different quality reference assemblies on PSMC analysis has not been tested to date. Moura *et al.*[17] mapped short read data from two individual killer whales to a draft assembly of the bottlenose dolphin (assembly turTru1, Ensembl database release 69.1) made up of 0.24 million scaffolds, with a scaffold N50 of 116,287, in which 94% of the scaffolds, comprising approximately 25% of the genome, are less than 50-kb long. Moura *et al.*[17] thereby generated a 20× average coverage sequence and a 13× average coverage sequence, and did not apply a correction for differences in false negative rate of detection of heterozygotes due to the difference in coverage. The PSMC plots from the two genomes presented in Moura *et al.*[17] do not converge in effective population size even though the two individuals shared a relatively recent common ancestor (TMRCA estimated at ~150 KYA by the same authors[22]).

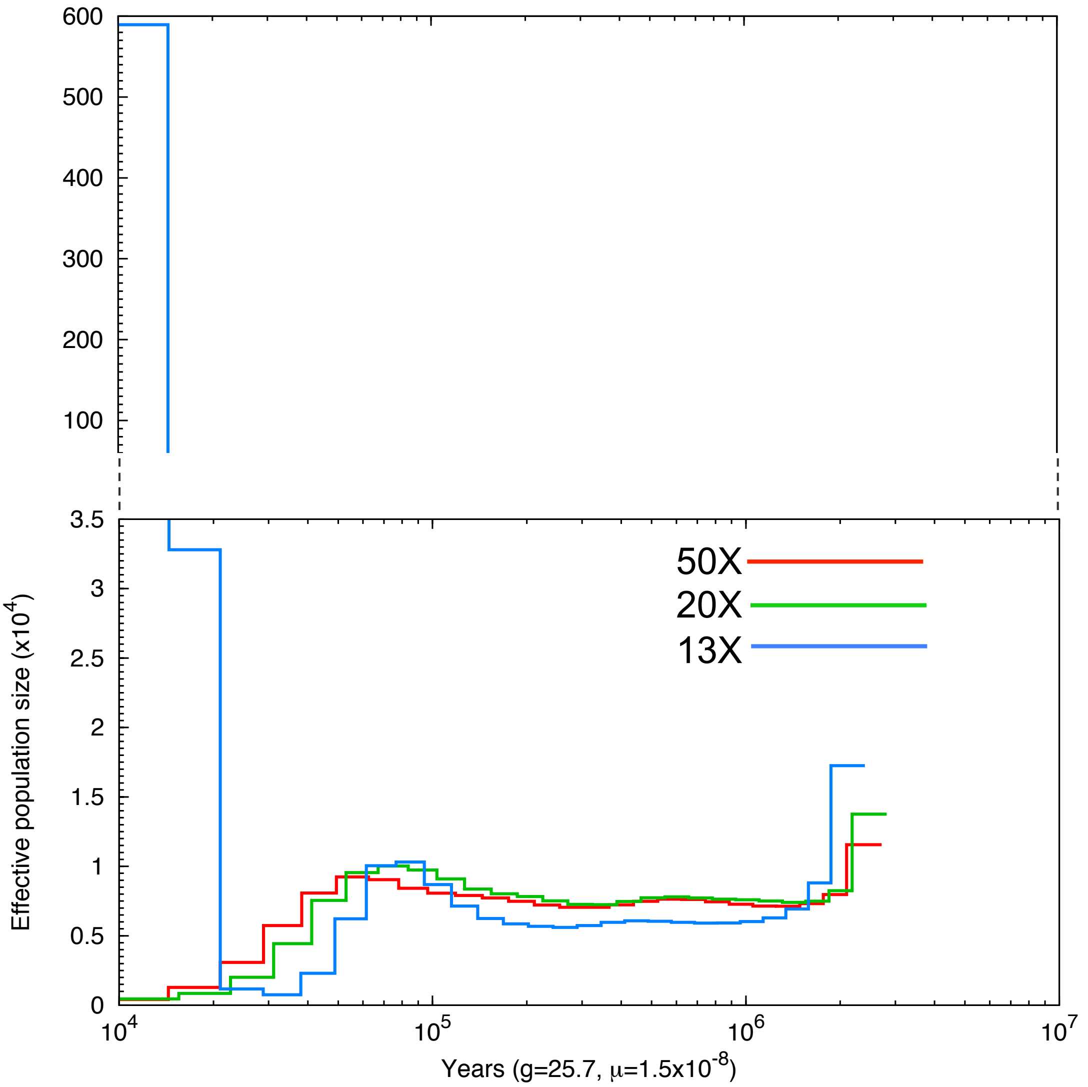

Supplementary Figure 10. PSMC plots of inferred demographic history of the same individual using 13×, 20× and 50× coverage of sequencing data. In order to better understand if these methodological issues (<20× coverage sequence data mapped to a highly fragmented reference) led to erroneous inference of the demographic histories and the underlying processes in this previous study[17], PSMC was used to analyse down-sampled versions of the high coverage North Atlantic killer whale genome. A 50× bam file was produced by mapping the short read data generated from the North Atlantic killer whale to scaffolds of the autosomal regions of the high quality killer whale reference genome that were greater than 10-Mb in length, totaling 1.5 Gb and which had all repeat regions masked as noted above. The 50× coverage bam file was then down-sampled to produce 13× and 20× coverage bam files. A consensus sequence of each of the three bam files was then generated in fastq format sequentially using: firstly, SAMtools mpileup command with the –C50 option to reduce the effect of reads with excessive mismatches; secondly, bcftools view –c to call variants; lastly, vcfutils.pl vcf2fq to convert the vcf file of called variants to fastq format with further filtering to remove sites with less than a third or more than double the average depth of coverage and Phred quality scores less than 30. The PSMC inference was then carried out using the recommended input parameters for human autosomal data[21], i.e. 25 iterations, with maximum TMRCA (*T*max) = 15, number of atomic time intervals (*n*) = 64 (following the pattern (1*4 + 25*2 + 1*4 + 1*6), and

initial theta ratio (r) =5. For the initial comparison between the 13×, 20× and 50× coverage North Atlantic genomes, a generation time of 25.7 years and a mutation rate of 1.53×10^{-8} substitutions/nucleotide/generation were applied, as per reference 17. Comparison of the PSMC inference plots based on the 13×, 20× and 50× coverage files, generated from the same individual, highlighted the impact of coverage on inference of both the magnitude of *N*e at any given time and the timing of the changes in *N*e, consistent with findings by a previous study[76]. In particular, estimates of *N*e in more recent times based on the 13× genome assembly differed markedly to inferred *N*e from the 20× and 50× genome assemblies. This is a consequence of a higher false negative detection rate of heterozygote sites in the 13× genome assembly, producing the same effect as a smaller mutation rate would have on the plot. The PSMC plots of the 20× and 50× coverage North Atlantic genome were almost identical both regarding the timing and the magnitude of demographic events.

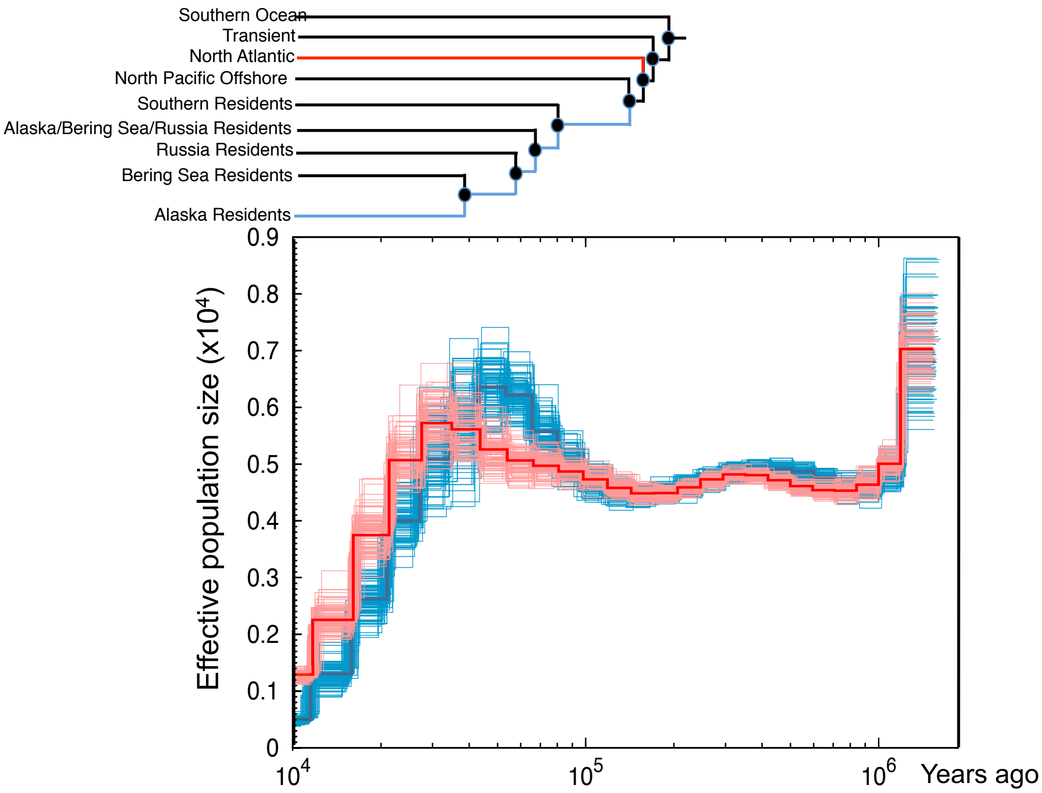

Supplementary Figure 11. Historical population sizes of a North Atlantic (red) and North Pacific resident killer whale (blue) inferred by pairwise sequential Markovian coalescent analysis (PSMC). All three plots of the North Atlantic killer whale genome (13×, 20× & 50×) in Supplementary Figure 10 are consistent in estimating a marked decline in *N*e between 100,000 years and 20,000 years ago. To better infer the process underlying this decline in *N*e, PSMC was used to compare equal coverage (20×) assemblies of the North Pacific and North Atlantic genomes. A 20× bam file was generated for the North Pacific killer whale using data from the short read archive (SRP035610)[17] and mapped as above. As with other inference methods based on coalescent theory, PSMC can only infer scaled times and population sizes. To convert these estimates into real time and size, all scaled results need to be divided by the mutation rate. To allow comparison of the relative timing of population splits with a published time-calibrated nuclear phylogeny based on RAD-seq data we scaled the PSMC plot using the same mutation rate as reference 22. Although the two papers by Moura *et al.*[17,22] were published almost concurrently, they use two different mutation rates for nuclear genomic data for each analysis: 1.53×10^{-8} substitutions/nucleotide /generation for PSMC[17] and an estimate almost double this rate for their time-calibrated phylogeny of 2.83×10^{-8} substitutions/nucleotide/generation, based on their given rate of 0.0011 substitutions per site per million years[22] and a generation time of 25.7 years as above. We therefore scaled the PSMC plots by a generation time of 25.7 years and a mutation rate of 2.83×10^{-8} substitutions/nucleotide/generation. A total number of 100 bootstraps were performed. The combined PSMC plot of both genomes is shown compared with population split times from a previously published time-calibrated phylogeny[22] that supports the inference that changes in inferred *N*e by PSMC are at least partially driven by changes in connectivity. The *x*-axis gives time measured by pairwise sequence divergence and the *y*-axis gives the effective population size measured by the scaled mutation rate of 2.83×10^{-8} substitutions

/nucleotide/generation and assuming a generation time of 25.7 years. Thin light lines of the same colour correspond to the 95% confidence intervals of PSMC inferences on 100 rounds of bootstrapped sequences. Insert shows a time-calibrated nuclear marker phylogeny adapted from reference 22, scaled using the same mutation rate and plotted on the same *x*-axis as the PSMC plots. The branches leading to the populations used in the PSMC plots are coloured accordingly and highlight that population splits are followed by changes in inferred N_e.

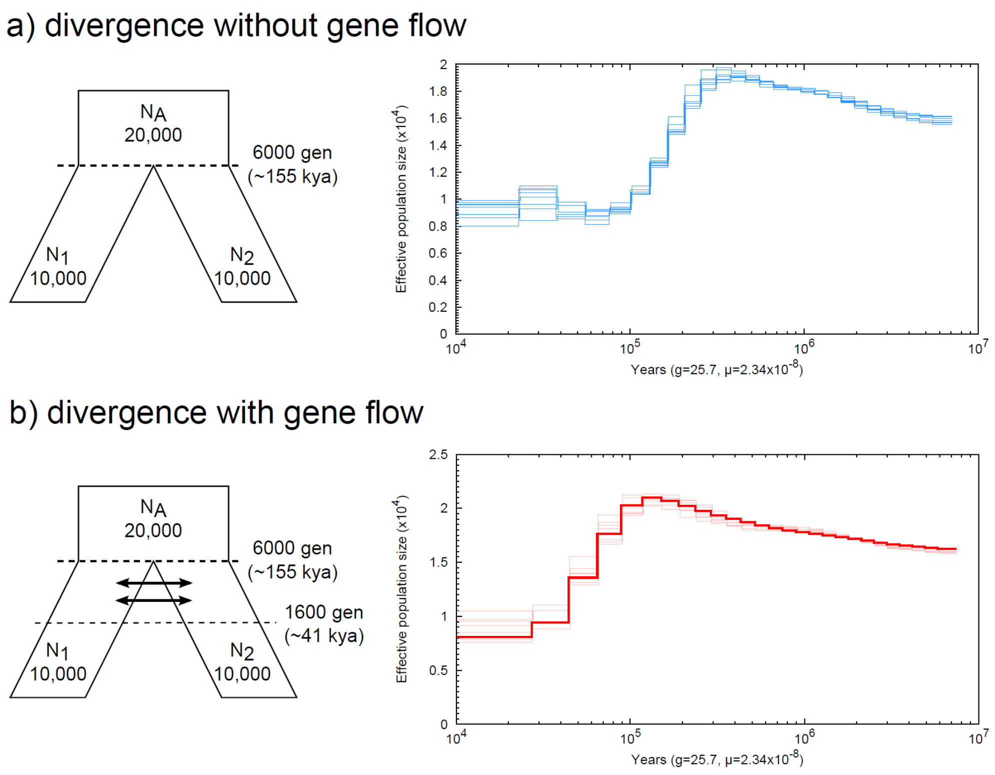

Supplementary Figure 12. Effect of population structure and divergence on PSMC. Finally, to further understand the relationship between connectivity and PSMC inference we simulated data consistent with a population split using scrm[77] and plotted the changes in effective population size inferred by PSMC. We considered two models of population divergence where an ancestral population of size N_A=20,000 splits 6,000 generations ago into two descending populations of size N_1=N_2=10,000. In **a)** populations become isolated after the split event and diverge without gene flow, representing a scenario of a sudden change in connectivity; whereas in **b)** there is a period of symmetric gene flow (2Nm=20) until 1600 generations ago, mimicking a gradual change in the connectivity of populations. We considered models with no changes in the size of populations, which in total remains 20,000, and hence these models could represent scenarios of divergence due to vicariance without founder events. As can be seen in the plots on the right, reductions in the connectivity of populations due to population divergence can lead to changes in the effective sizes inferred with PSMC. For both models, even though we did not simulate any demographic changes due to population bottlenecks or expansions, PSMC infers a gradual decline on the effective sizes after populations become more isolated, coinciding with the reduction in connectivity among populations. Note that these simulations are not intended to capture the recent demographic history of the killer whales, but rather illustrate that changes in population structure due to population divergence can lead to changes in the inferred PSMC effective sizes. We simulated and analysed with PSMC eight independent datasets for each model (corresponding to the different lines in the PSMC plots), assuming a mutation rate μ of 2.34×10^{-8} per site per generation, an arbitrary recombination rate set to 0.80*μ, and a generation time of 25.7 years. Each datasets consisted of 10 blocks of 100 Mb (total of 1,000 Mb) sampled from a single diploid individual from population 1, generated using *scrm* with the following command lines: a) ms 2 10 -t 93600 -r 74880 100000000 -I 2 2 0 0.0 -n 1 1 -n 2 1 -G 0.0 -ej 0.15 2 1 -en 0.15 1 2; b) ms 2 10 -t 93600 -r 74880 100000000 -I 2 2 0 0.0 -n 1 1 -n 2 1 -G 0.0 -m 1 2 0 -m 2 1 0 -em

0.04 1 2 80 -em 0.04 2 1 80 -ej 0.15 2 1 -em 0.15 1 2 0 -em 0.15 2 1 0 -en 0.15 1 2.

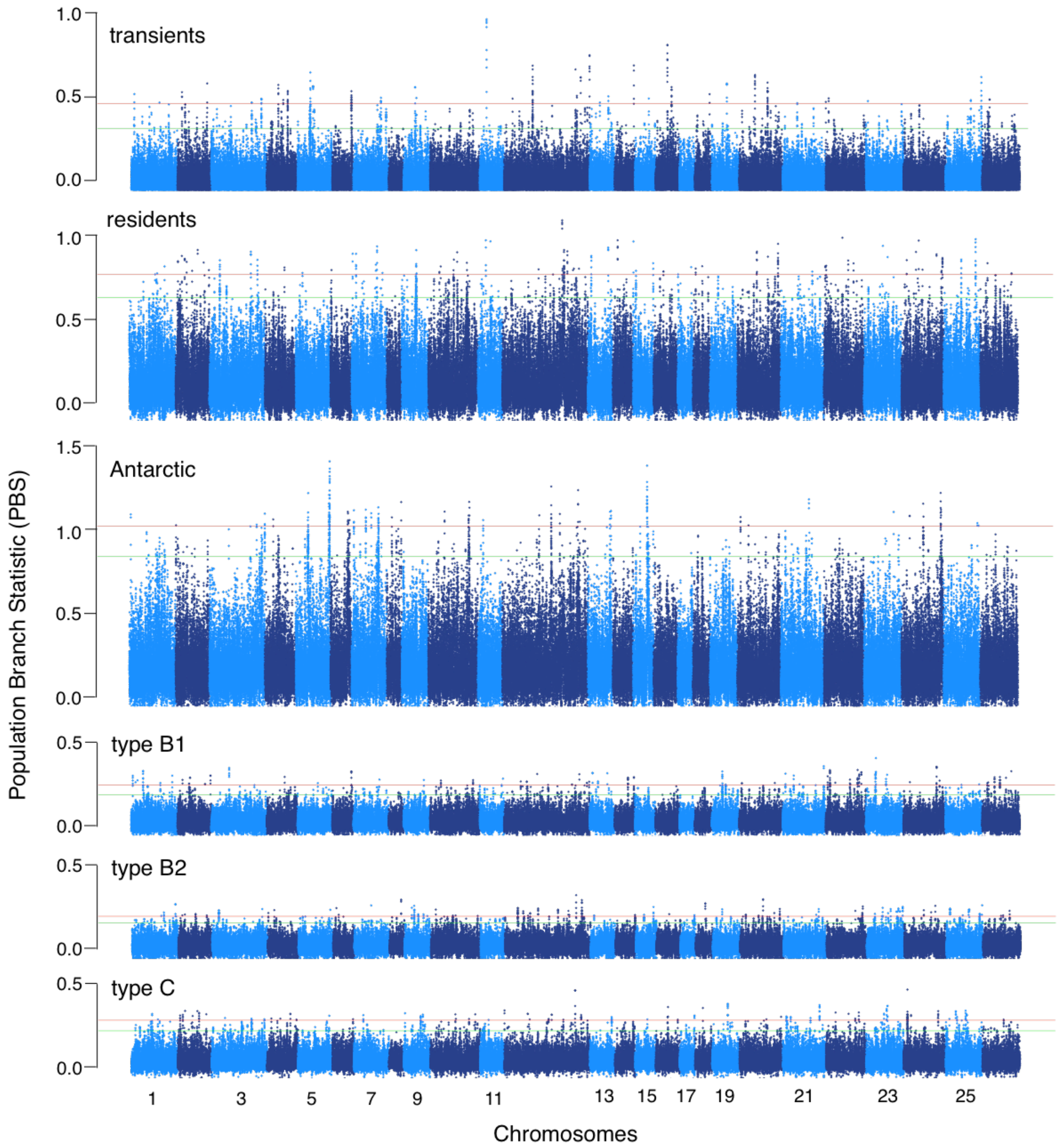

Supplementary Figure 13. Population-specific allele frequency changes estimated as F_{ST}-based branch lengths in 50-kb sliding windows. The mean population branch statistic (PBS) is highest along the two branches that were inferred by TreeMix to have undergone the highest amounts of drift: the branch to the common ancestor of the Antarctic types and the branch to the *resident* ecotype. The green and red lines indicate the 99.5 and 99.9 percentiles respectively. Several distinct peaks are seen in the Manhattan plots for each branch, these are further explored using PBS estimates at the exon level to identify candidate loci that have potentially evolved under selection.

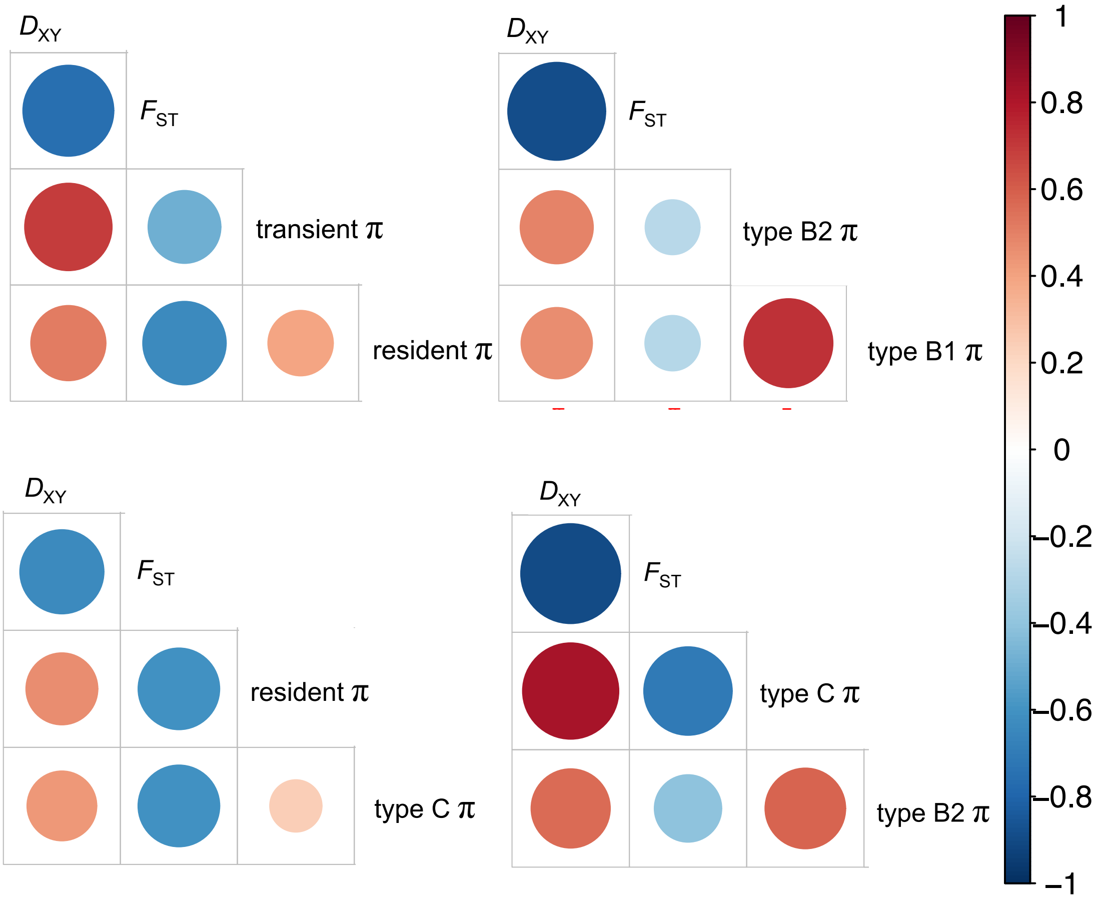

Supplementary Figure 14. Correlations between (50-kb) window-based estimates of genome-wide divergence (*D*xy), differentiation (F_{ST}) and nucleotide diversity (π) for the pairwise comparison between ecotypes shown in Fig. 4A. Red circles indicate a positive relationship, blue a negative one; colour intensity and circle size are proportional to Spearman's correlation coefficient. Shared regions of high differentiation, but low diversity and divergence are likely to have been regions on linked selection on the ancestral form, which would remove diversity and result in rapid lineage sorting of allele frequencies due to differential drift and selection in the derived forms.

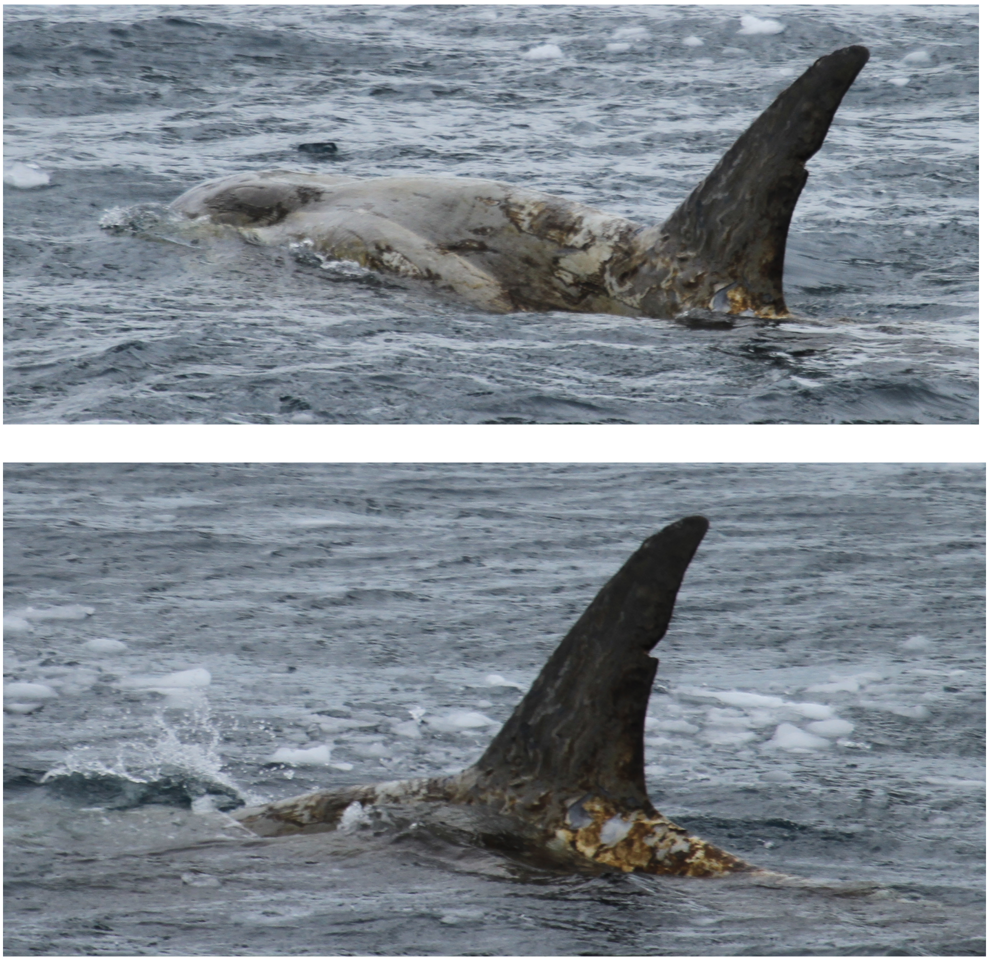

Supplementary Figure 15. Photograph of an Antarctic *type B1* killer whale photographed by Conor Ryan in the Gerlache Strait of the Antarctic Peninsula on the 4th December 2015. The individual was travelling alone and moving slowly. The skin was peeling off as evidenced in the photograph. Those of us that have conducted field studies over many years have not previously encountered any killer whales with a skin condition such as this. A previous study[42] has detailed that Antarctic killer whales make rapid round-trips of up to 9,400 km from Antarctica (less than 60° South) to subtropical waters (30-37° South) where sea surface temperature was approximately 20-25°C warmer using satellite tag data. Durban & Pitman[42] additionally documented that the same individuals were encountered with the different amounts of accumulation of diatoms on their skin at different times. They hypothesised that these rapid migrations to warm water may allow for epidermal tissue regeneration without the thermal cost that would be incurred if skin regeneration took place in Antarctic waters[42]. The *type B1* individual photographed by Conor Ryan in Antarctic waters highlights that skin regeneration may be a strong selective force. The results of this study highlight genomic adaptation of a gene (*FAM83H*) associated with epidermal regeneration along the branch to the ancestral Antarctic lineage that include four non-synonymous substitutions.

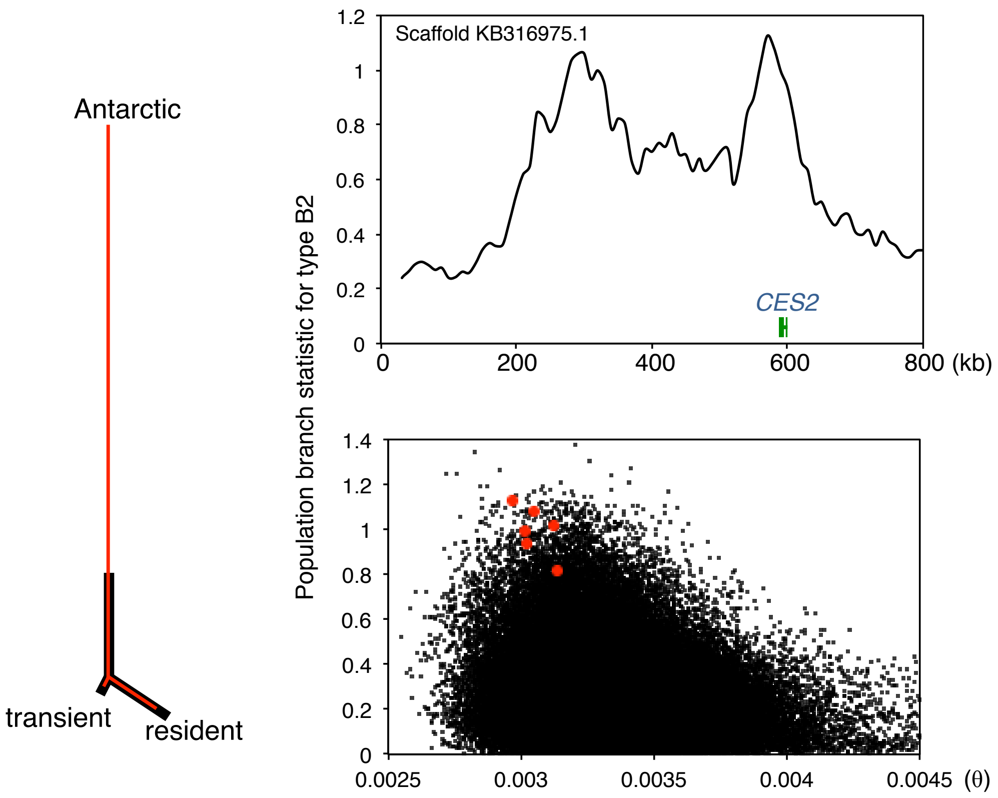

Supplementary Fig 16. Population-specific allele frequency changes are indicated by the F_{ST}-based branch lengths (red) for the *CES2* gene overlaid on the genome-wide average branch lengths (black), indicating substantial differentiation along the branch to the common ancestor of the Antarctic ecotypes; the distribution of population branch statistic (PBS) along scaffold KB317017 and as a function of mutation rate (θ) calculated in 50-kb sliding windows. Windows overlapping the CES2 gene are coloured red and are in the 99-99.9 percentile.

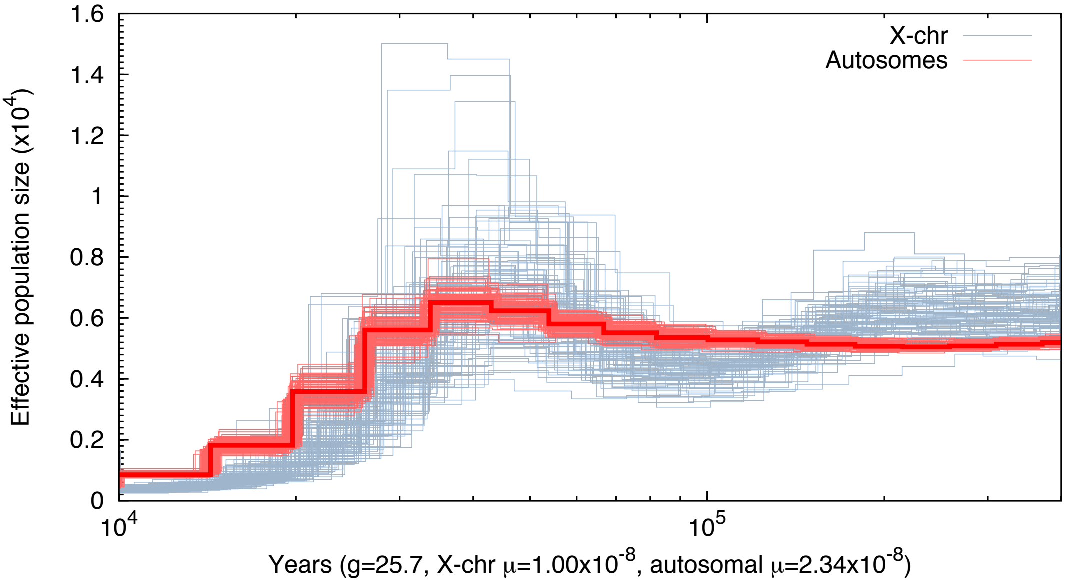

Supplementary Fig. 17, PSMC estimates of changes in N_e over time inferred from the autosomes (N_{eA}, red) and the X-chromosome (N_{eX}, grey) of the high coverage genome sequence of a North Atlantic female killer whale. Thick lines represent the median, thin light lines of the same colour correspond to 100 rounds of bootstrapping. To make the autosomal plot and X-chromosome synchronise in the inferred timing of demographic change, requires scaling the X-chromosome plot by a mutation rate of 1.0×10^{-8} substitutions/nucleotide/generation if the autosomal mutation rate is assumed to be 2.34×10^{-8} substitutions/nucleotide/generation[20]. This requires a male-to-female mutation rate ratio to be >100, making it seemingly biologically unrealistic.

Supplementary Tables

Supplementary Table 1. Overview of sample information and sequencing statistics and accession numbers in the European Nucleotide Archive (study accession number: PRJEB7375). The number of sites covered at ≥1 and ≥2× in the repeat-masked genomes are given. Sample ID's are from the SWFSC Marine Mammal and Sea Turtle Research Collection database.

Ecotype	Sample ID	No. sites covered 1X	No. sites covered ≥2X	Accession Number
transient	Z79751	912,838,213	581,568,661	ERS554458
	Z40246	450,961,532	117,156,041	ERS554453
	Z94446	1,078,915,238	926,346,260	ERS554461
	Z57910	922,002,453	620,949,972	ERS554454
	Z57919	896,799,085	569,918,216	ERS554455
	Z67975	848,029,101	499,244,722	ERS554457
	Z62471	816,837,489	453,988,372	ERS554456
	Z93820	785,932,259	437,725,622	ERS554460
	Z79759	1,065,456,348	919,518,022	ERS554459
	Z28549	324,432,898	64,246,642	ERS554452
resident	Z62250	520,118,250	209,645,749	ERS554451
	Z126161	910,563,765	592,486,217	ERS554443
	Z35322	936,077,728	637,183,391	ERS554450
	Z126163	1,018,566,267	786,136,456	ERS554444
	Z31868	922,308,161	609,051,768	ERS554449
	Z126165	578,454,141	248,968,267	ERS554445
	Z126178	991,162,998	729,347,947	ERS554448
	Z126167	966,067,389	686,237,346	ERS554446
	Z126169	939,922,125	639,478,143	ERS554447
	Z126158	987,961,149	727,028,730	ERS554442
	Z157515	1,135,204,773	1,106,693,447	SRP035610
type C	Z88353	669,462,810	299,227,990	ERS554471
	Z26627	856,354,026	519,220,516	ERS554468
	Z26626	445,290,470	123,931,174	ERS554467
	Z45804	914,040,648	593,451,716	ERS554470
	Z26614	888,571,774	553,219,110	ERS554462
	Z26619	990,054,479	729,620,205	ERS554464
	Z45800	710,495,576	336,430,867	ERS554469
	Z26620	874,869,923	546,880,892	ERS554465
	Z26617	681,130,270	315,413,786	ERS554463
	Z26623	949,838,075	639,223,700	ERS554466

Supplementary Table 1. Continued.

type B1	Z78580	730,740,473	372,942,192	ERS554426
	Z78581	509,607,459	171,867,125	ERS554427
	Z124038	885,646,983	563,137,421	ERS554428
	Z32005	657,449,966	267,338,920	ERS554424
	Z124047	183,708,091	26,914,304	ERS554429
	Z88344	809,373,312	436,925,282	ERS554430
	Z73077	1,150,930,500	1,096,203,119	ERS554425
type B2	Z124036	651,122,155	332,981,742	ERS554431
	Z124043	762,936,262	389,551,088	ERS554432
	Z31884	1,028,784,218	804,933,446	ERS554433
	Z32009	1,032,340,577	816,648,000	ERS554434
	Z40882	952,005,121	673,561,894	ERS554435
	Z78595	944,613,699	672,705,181	ERS554436
	Z92354	574,697,414	210,386,441	ERS554437
	Z92363	870,395,218	526,946,499	ERS554439
	Z92362	135,880,034	18,881,497	ERS554438
	Z92365	845,849,210	524,230,712	ERS554440
	Z92367	596,339,488	249,249,018	ERS554441
Atlantic		1,199,341,331	1,191,738,985	PRJNA167475

Supplementary Table 2. A comparison of mean F_{ST} values estimated from low coverage whole genome sequencing (WGS) data generated for this study and high coverage published SNP-typing[11] and RAD-seq data[19]. F_{ST} estimates from the RAD-seq data are from Table 1 of ref 19 and are based on those inferred by the authors to be putatively neutral loci and exclude 347 outliers out of a total of 3,281 SNPs. These estimates derived from the RAD-seq data are therefore expected to be marginally downwardly biased compared to the WGS and SNP-typing estimates. F_{ST} values based on multi-allelic microsatellite data are biased by high heterozygosity, thereby reducing $F_{ST,}$ so that values between microsatellite and SNP analyses are therefore not directly comparable.

	**WGS**	**SNP-typing**	**RAD-seq**
Between Antarctic v Pacific ecotypes			
type B1 v resident	0.56	0.68	-
type B2 v resident	0.57	0.64	-
type C v resident	0.57	0.61	-
type B1 v transient	0.37	0.34	-
type B2 v transient	0.4	0.34	-
type C v transient	0.39	0.30	-
Between Pacific ecotypes			
transient v resident	0.32	0.28	(SR v AT) 0.30
			(SR v CT) 0.29
			(AR v AT) 0.27
			(AR v CT) 0.26
			(BS v AT) 0.27
			(BS v CT) 0.26
Between Antarctic ecotypes			
type B1 v type B2	0.09	0.141	-
type B2 v type C	0.13	0.103	-
type B1 v type C	0.13	0.103	-

Supplementary Table 3. Counts of inferred transitions and transversions from the ancestral state based on comparison with the bottlenose dolphin genome sequence at third codon positions found in type B1, and counts of sites in the two individuals of each of the other four ecotypes in which only the ancestral rather than the derived state were found. This provides an account of the accumulation of derived alleles along the branch to type B1 allowing the estimation of an approximate time to most recent common ancestor.

Transitions from ancestral state	Number of transitions from ancestral state in type B1	Number of sites with ancestral rather than derived state							
		type B2	type B2	resident	resident	transient	transient	type C	type C
T->C	2639	28	25	42	40	35	38	23	22
C->T	3546	89	78	198	206	200	189	87	88
A->G	2477	26	25	53	52	47	47	29	29
G->A	3131	61	72	169	167	168	162	77	80
Total	11793	204	200	462	465	450	436	216	219

Transversions from ancestral state	Number of tranversions from ancestral state in type B1	Number of sites with ancestral rather than derived state							
		type B2	type B2	resident	resident	transient	transient	type C	type C
GA->C	1979	11	7	36	37	33	36	12	13
GA->T	1498	9	10	22	23	22	21	8	9
CT->A	1825	16	14	35	34	33	32	21	12
CT->G	1995	14	15	30	30	28	31	13	13
Total	7297	50	46	123	124	116	120	54	47

Supplementary Table 4. Summary results for the three-population test of the form *f*3(A;B,C), where a significantly negative value of the *f*3 statistic implies that population A is admixed[26]. The three-population tests were re-estimated with the Atlantic genome added, which resulted in additional inferred admixture events among ecotypes. These results are reported in Supplementary Table 5.

Target population	Source population 1	Source population 2	F3 statistic	SE (*F*-statistic)	*Z*-score
type B1	type B2	resident	-0.0017	9.0×10^{-6}	-186.4
type B1	type B2	transient	-0.0015	7.6×10^{-6}	-193.4
type B1	type B2	type C	-0.0007	5.7×10^{-6}	-125.9
type B1	resident	transient	0.0168	3.2×10^{-5}	532.8
type B1	resident	type C	-0.0006	1.1×10^{-5}	-59.4
type B1	transient	type C	-0.0005	8.8×10^{-6}	-53.9
type B2	type B1	resident	0.0011	9.8×10^{-6}	109.5
type B2	type B1	transient	0.0009	8.3×10^{-6}	104.7
type B2	type B1	type C	0.0001	5.3×10^{-6}	21.4
type B2	resident	transient	0.0194	3.4×10^{-5}	572.4
type B2	resident	type C	0.0012	1.0×10^{-5}	114.6
type B2	transient	type C	0.0011	8.5×10^{-6}	130.1
resident	type B1	type B2	0.0316	5.3×10^{-5}	598.9
resident	type B1	transient	0.013	2.8×10^{-5}	460.3
resident	type B1	type C	0.0305	5.3×10^{-5}	573.4
resident	type B2	transient	0.0132	2.9×10^{-5}	461
resident	type B2	type C	0.0315	5.5×10^{-5}	576.5
resident	transient	type C	0.0132	2.9×10^{-5}	452.1
transient	type B1	type B2	0.0181	3.1×10^{-5}	583.3
transient	type B1	resident	-0.0002	1.3×10^{-5}	-13.7
transient	type B1	type C	0.0171	3.1×10^{-5}	552.6
transient	type B2	resident	-0.0004	1.3×10^{-5}	-29.5
transient	type B2	type C	0.0179	3.2×10^{-5}	557
transient	resident	type C	-0.0003	1.3×10^{-5}	-25.3
type C	type B1	type B2	0.0013	6.7×10^{-6}	193.7
type C	type B1	resident	0.0012	1.2×10^{-5}	104.3
type C	type B1	transient	0.0011	9.5×10^{-6}	111
type C	type B2	resident	0.0003	1.0×10^{-5}	24.2
type C	type B2	transient	0.0003	8.6×10^{-6}	35.8
type C	resident	transient	0.0185	3.6×10^{-5}	517.6

Supplementary Table 5. Summary results for the three-population test of the form *f*3(A;B,C) which include comparison with the high coverage Atlantic genome. A significantly negative value of the *f*3 statistic implies that population A is admixed[26].

Target population	Source population 1	Source population 2	f3 statistic	SE (f - statistic)	*Z*-score
Atlantic	resident	type B1	0.074	0.0011	67.0
Atlantic	resident	type B2	0.074	0.0011	67.1
Atlantic	resident	type C	0.074	0.0011	67.1
Atlantic	resident	transient	0.069	0.0010	64.9
Atlantic	type B1	type B2	0.084	0.0012	71.3
Atlantic	type B1	type C	0.085	0.0012	71.2
Atlantic	type B1	transient	0.074	0.0011	67.5
Atlantic	type B2	type C	0.084	0.0012	71.2
Atlantic	type B2	transient	0.074	0.0011	67.5
Atlantic	type C	transient	0.074	0.0011	67.5
resident	Atlantic	type B1	-0.0022	$9.4x10^{-5}$	-23.3
resident	Atlantic	type B2	-0.0022	$9.2x10^{-5}$	-24.0
resident	Atlantic	type C	-0.0021	$9.2x10^{-5}$	-22.5
resident	Atlantic	transient	-0.0031	$8.7x10^{-5}$	34.8
type B1	Atlantic	resident	0.0079	0.0001	54.4
type B1	Atlantic	type B2	-0.0027	$4.34x10^{-5}$	-63.21
type B1	Atlantic	type C	-0.0024	$4.78x10^{-5}$	-50.75
type B1	Atlantic	transient	0.0075	0.0001	57.1
type B2	Atlantic	type B1	-0.0016	$4.11x10^{-5}$	-38.6
type B2	Atlantic	type C	-0.0013	$4.10x10^{-5}$	-30.7
type B2	Atlantic	transient	0.0086	0.0001	70.4
type B2	Atlantic	resident	0.0091	0.0001	66.0
type C	Atlantic	type B1	-0.0015	$4.58x10^{-5}$	-33.5
type C	Atlantic	type B2	-0.0014	$2.20x10^{-5}$	-63.4
type C	Atlantic	transient	0.0085	0.0001	68.0
type C	Atlantic	resident	0.0089	0.0001	64.3
transient	Atlantic	type B1	-0.0075	$7.42x10^{-5}$	-101.6
transient	Atlantic	type B2	-0.0075	$7.26x10^{-5}$	-103.9
transient	Atlantic	type C	-0.0074	$7.38x10^{-5}$	-100.4
transient	Atlantic	resident	-0.0019	$6.94x10^{-5}$	-26.9

Supplementary Table 6. Top 20 significantly enriched GO: terms based on the top 1% Fst outliers in pairwise comparisons of Antarctic ecotypes with Pacific ecotypes.

	GO.ID	Term	Annotated	Significant	Expected	P-value (classic Fisher)
1	GO:0060612	adipose tissue development	6	2	0.06	0.0015
2	GO:0030001	metal ion transport	499	13	5	0.0015
3	GO:0006811	ion transport	931	19	9.33	0.0023
4	GO:0060134	prepulse inhibition	8	2	0.08	0.0027
5	GO:0006812	cation transport	613	14	6.14	0.0033
6	GO:0032026	response to magnesium ion	10	2	0.1	0.0043
7	GO:0006817	phosphate ion transport	11	2	0.11	0.0052
8	GO:0001824	blastocyst development	37	3	0.37	0.006
9	GO:0055085	transmembrane transport	658	14	6.59	0.0061
10	GO:0031424	keratinization	12	2	0.12	0.0062
11	GO:0045444	fat cell differentiation	76	4	0.76	0.0071
12	GO:0001964	startle response	14	2	0.14	0.0084
13	GO:0032413	negative regulation of ion transmembrane transporter activity	14	2	0.14	0.0084
14	GO:0006814	sodium ion transport	126	5	1.26	0.0087
15	GO:0016925	protein sumoylation	15	2	0.15	0.0096
16	GO:0001835	blastocyst hatching	1	1	0.01	0.01
17	GO:0003215	cardiac right ventricle morphogenesis	1	1	0.01	0.01
18	GO:0003284	septum primum development	1	1	0.01	0.01
19	GO:0003289	atrial septum primum morphogenesis	1	1	0.01	0.01
20	GO:0007161	calcium-independent cell-matrix adhesion	1	1	0.01	0.01

Supplementary Table 7. Top 20 GO-terms of biological processes enriched in the top 99.9 (resident, transient and Antarctic branches) and top 99.99 percentile (type B1, type B2 and type C).

	GO ID	Term	Annotated	Significant	Expected	P-value (classic Fisher)
**type B1**						
1	GO:0051571	positive regulation of histone H3-K4 methylation	2	1	0	0.0013
2	GO:0010216	maintenance of DNA methylation	3	1	0	0.0019
3	GO:0051573	negative regulation of histone H3-K9 methylation	3	1	0	0.0019
4	GO:0001880	Mullerian duct regression	4	1	0	0.0025
5	GO:0031062	positive regulation of histone methylation	4	1	0	0.0025
6	GO:0050913	sensory perception of bitter taste	4	1	0	0.0025
7	GO:0050916	sensory perception of sweet taste	4	1	0	0.0025
8	GO:0051569	regulation of histone H3-K4 methylation	4	1	0	0.0025
9	GO:0051570	regulation of histone H3-K9 methylation	4	1	0	0.0025
10	GO:0006730	one-carbon metabolic process	127	2	0.08	0.0027
11	GO:0031061	negative regulation of histone methylation	5	1	0	0.0032
12	GO:0050917	sensory perception of umami taste	5	1	0	0.0032
13	GO:0051567	histone H3-K9 methylation	6	1	0	0.0038
14	GO:0060033	anatomical structure regression	6	1	0	0.0038
15	GO:0061647	histone H3-K9 modification	6	1	0	0.0038
16	GO:0031060	regulation of histone methylation	7	1	0	0.0044
17	GO:0031057	negative regulation of histone modification	10	1	0.01	0.0063
18	GO:0045123	cellular extravasation	10	1	0.01	0.0063
19	GO:0051568	histone H3-K4 methylation	11	1	0.01	0.0070
20	GO:0031058	positive regulation of histone modification	13	1	0.01	0.0082
**type B2**						
1	GO:0002115	store-operated calcium entry	2	1	0	0.0013
2	GO:0032237	activation of store-operated calcium channel activity	4	1	0	0.0025
3	GO:1901339	regulation of store-operated calcium channel activity	4	1	0	0.0025
4	GO:1901341	positive regulation of store-operated calcium channel activity	4	1	0	0.0025
5	GO:0007185	transmembrane receptor protein tyrosine phosphatase signaling pathway	7	1	0	0.0044
6	GO:1901021	positive regulation of calcium ion transmembrane transporter activity	7	1	0	0.0044
7	GO:2001259	positive regulation of cation channel activity	7	1	0	0.0044
8	GO:0035023	regulation of Rho protein signal transduction	189	2	0.12	0.0059
9	GO:0046578	regulation of Ras protein signal transduction	222	2	0.14	0.0081
10	GO:0032414	positive regulation of ion transmembrane transporter activity	13	1	0.01	0.0082
11	GO:0007266	Rho protein signal transduction	227	2	0.14	0.0085
12	GO:1901019	regulation of calcium ion transmembrane transporter activity	14	1	0.01	0.0089
13	GO:1903169	regulation of calcium ion transmembrane transport	14	1	0.01	0.0089
14	GO:2001257	regulation of cation channel activity	14	1	0.01	0.0089
15	GO:0032411	positive regulation of transporter activity	15	1	0.01	0.0095
16	GO:0034767	positive regulation of ion transmembrane transport	15	1	0.01	0.0095
17	GO:0051056	regulation of small GTPase mediated signal transduction	257	2	2	0.0107
18	GO:0070588	calcium ion transmembrane transport	17	1	0.01	0.0108
19	GO:0046928	regulation of neurotransmitter secretion	19	1	0.01	0.0120
20	GO:0030574	collagen catabolic process	20	1	0.01	0.0127
**type C**						
1	GO:0034124	regulation of MyD88-dependent toll-like receptor signaling pathway	1	1	0	0.00056
2	GO:0034126	positive regulation of MyD88-dependent toll-like receptor signaling pathway	1	1	0	0.00056
3	GO:0002253	activation of immune response	111	2	0.06	0.00158
4	GO:0010842	retina layer formation	3	1	0	0.00167
5	GO:0002755	MyD88-dependent toll-like receptor signaling pathway	6	1	0	0.00334
6	GO:0050778	positive regulation of immune response	177	2	0.1	0.00395
7	GO:0034123	positive regulation of toll-like receptor signaling pathway	8	1	0	0.00445
8	GO:0045087	innate immune response	195	2	0.11	0.00477
9	GO:0003407	neural retina development	10	1	0.01	0.00556
10	GO:0045123	cellular extravasation	10	1	0.01	0.00556
11	GO:0050776	regulation of immune response	214	2	0.12	0.00572
12	GO:0034121	regulation of toll-like receptor signaling pathway	11	1	0.01	0.00611
13	GO:0006957	complement activation, alternative pathway	12	1	0.01	0.00666
14	GO:0001937	negative regulation of endothelial cell proliferation	15	1	0.01	0.00832
15	GO:0031290	retinal ganglion cell axon guidance	15	1	0.01	0.00832
16	GO:0006935	chemotaxis	266	2	0.15	0.00873
17	GO:0042330	taxis	266	2	0.15	0.00873
18	GO:0050798	activated T cell proliferation	16	1	0.01	0.00888
19	GO:0010596	negative regulation of endothelial cell migration	17	1	0.01	0.00943
20	GO:0007155	cell adhesion	875	3	0.49	0.00951

**Branch to shared ancestor of Antarctic types**						
1	GO:0060211	regulation of nuclear-transcribed mRNA poly(A) tail shortening	1	1	0.01	0.0052
2	GO:0060213	positive regulation of nuclear-transcribed mRNA poly(A) tail shortening	1	1	0.01	0.0052
3	GO:0061013	regulation of mRNA catabolic process	1	1	0.01	0.0052
4	GO:0061014	positive regulation of mRNA catabolic process	1	1	0.01	0.0052
5	GO:1900151	regulation of nuclear-transcribed mRNA catabolic process, deadenylation-dependent decay	1	1	0.01	0.0052
6	GO:1900153	positive regulation of nuclear-transcribed mRNA catabolic process, deadenylation-dependent decay	1	1	0.01	0.0052
7	GO:0000289	nuclear-transcribed mRNA poly(A) tail shortening	2	1	0.01	0.0105
8	GO:0006196	AMP catabolic process	2	1	0.01	0.0105
9	GO:0015682	ferric iron transport	2	1	0.01	0.0105
10	GO:0030327	prenylated protein catabolic process	2	1	0.01	0.0105
11	GO:0030328	prenylcysteine catabolic process	2	1	0.01	0.0105
12	GO:0030329	prenylcysteine metabolic process	2	1	0.01	0.0105
13	GO:0033572	transferrin transport	2	1	0.01	0.0105
14	GO:0050779	RNA destabilization	2	1	0.01	0.0105
15	GO:0072512	trivalent inorganic cation transport	2	1	0.01	0.0105
16	GO:0006091	generation of precursor metabolites and energy	276	5	1.45	0.0148
17	GO:0009128	purine nucleoside monophosphate catabolic process	3	1	0.02	0.0157
18	GO:0009158	ribonucleoside monophosphate catabolic process	3	1	0.02	0.0157
19	GO:0009169	purine ribonucleoside monophosphate catabolic process	3	1	0.02	0.0157
20	GO:0030033	purine ribonucleoside monophosphate catabolic process	3	1	0.02	0.0157

**resident**						
1	GO:0060562	epithelial tube morphogenesis	166	4	0.5	0.0015
2	GO:0035239	tube morphogenesis	180	4	0.54	0.0021
3	GO:0048546	digestive tract morphogenesis	23	2	0.07	0.0022
4	GO:0001702	gastrulation with mouth forming second	24	2	0.07	0.0024
5	GO:0003215	cardiac right ventricle morphogenesis	1	1	0	0.0030
6	GO:0003284	septum primum development	1	1	0	0.0030
7	GO:0003289	atrial septum primum morphogenesis	1	1	0	0.0030
8	GO:0007443	Malpighian tubule morphogenesis	1	1	0	0.0030
9	GO:0032793	positive regulation of CREB transcription factor activity	1	1	0	0.0030
10	GO:0071372	cellular response to follicle-stimulating hormone stimulus	1	1	0	0.0030
11	GO:0072002	Malpighian tubule development	1	1	0	0.0030
12	GO:2000055	positive regulation of Wnt signaling pathway involved in dorsal/ventral axis specification	1	1	0	0.0030
13	GO:0001947	heart looping	33	2	0.1	0.0044
14	GO:0003143	embryonic heart tube morphogenesis	33	2	0.1	0.0044
15	GO:0061371	determination of heart left/right asymmetry	33	2	0.1	0.0044
16	GO:0048863	stem cell differentiation	118	3	0.36	0.0054
17	GO:0003283	atrial septum development	2	1	0.01	0.006
18	GO:0003344	pericardium morphogenesis	2	1	0.01	0.006
19	GO:0035021	negative regulation of Rac protein signal transduction	2	1	0.01	0.006
20	GO:0036315	cellular response to sterol	2	1	0.01	0.006

**transient**						
1	GO:0060056	mammary gland involution	7	2	0.04	0.0007
2	GO:0044248	cellular catabolic process	1039	15	6.11	0.00091
3	GO:0044712	single-organism catabolic process	629	11	3.7	0.00106
4	GO:0040036	regulation of fibroblast growth factor receptor signaling pathway	13	2	0.08	0.00256
5	GO:0045648	positive regulation of erythrocyte differentiation	13	2	0.08	0.00256
6	GO:0009056	catabolic process	1199	15	7.06	0.00375
7	GO:1901575	organic substance catabolic process	1107	14	6.51	0.0047
8	GO:0030218	erythrocyte differentiation	62	3	0.36	0.00577
9	GO:0000379	tRNA-type intron splice site recognition and cleavage	1	1	0.01	0.00589
10	GO:0006535	cysteine biosynthetic process from serine	1	1	0.01	0.00589
11	GO:0007079	mitotic chromosome movement towards spindle pole	1	1	0.01	0.00589
12	GO:0009757	hexose mediated signaling	1	1	0.01	0.00589
13	GO:0010182	sugar mediated signaling pathway	1	1	0.01	0.00589
14	GO:0010255	glucose mediated signaling pathway	1	1	0.01	0.00589
15	GO:0019343	cysteine biosynthetic process via cystathionine	1	1	0.01	0.00589
16	GO:0033690	positive regulation of osteoblast proliferation	1	1	0.01	0.00589
17	GO:0042636	negative regulation of hair cycle	1	1	0.01	0.00589
18	GO:0043418	homocysteine catabolic process	1	1	0.01	0.00589
19	GO:0045978	negative regulation of nucleoside metabolic process	1	1	0.01	0.00589
20	GO:0051799	negative regulation of hair follicle development	1	1	0.01	0.00589

Supplementary Table 8. Top 20 GO-terms of biological processes enriched in the top 99.9 (resident, transient and Antarctic branches) and top 99.99 percentile (type B1, type B2 and type C).

	GO ID	Term	Annotated	Significant	Expected	P-value (Classic Fisher)
**type B1**						
1	GO:0000822	inositol hexakisphosphate binding	1	1	0	0.00059
2	GO:0070679	inositol 1,4,5 trisphosphate binding	2	1	0	0.00118
3	GO:0005220	inositol 1,4,5-trisphosphate-sensitive calcium-release channel activity	3	1	0	0.00177
4	GO:0043533	inositol 1,3,4,5 tetrakisphosphate binding	3	1	0	0.00177
5	GO:0005057	receptor signaling protein activity	124	2	0.07	0.00224
6	GO:0003886	DNA (cytosine-5-)-methyltransferase activity	4	1	0	0.00236
7	GO:0008157	protein phosphatase 1 binding	4	1	0	0.00236
8	GO:0009008	DNA-methyltransferase activity	5	1	0	0.00295
9	GO:0030618	transforming growth factor beta receptor, pathway-specific cytoplasmic mediator activity	5	1	0	0.00295
10	GO:0008168	methyltransferase activity	164	2	0.1	0.00389
11	GO:0016741	transferase activity, transferring one-carbon groups	171	2	0.1	0.00422
12	GO:0015278	calcium-release channel activity	8	1	0	0.00472
13	GO:0032395	MHC class II receptor activity	9	1	0.01	0.0053
14	GO:0005072	transforming growth factor beta receptor, cytoplasmic mediator activity	10	1	0.01	0.00589
15	GO:0005217	intracellular ligand-gated ion channel activity	14	1	0.01	0.00824
16	GO:0004089	carbonate dehydratase activity	15	1	0.01	0.00883
17	GO:0004871	signal transducer activity	1726	4	1.02	0.01201
18	GO:0060089	molecular transducer activity	1726	4	1.02	0.01201
19	GO:0004864	protein phosphatase inhibitor activity	22	1	0.01	0.01
20	GO:0019212	phosphatase inhibitor activity	24	1	0.01	0.01
**type B2**						
1	GO:0005089	Rho guanyl-nucleotide exchange factor activity	69	2	0.05	0.009
2	GO:0005088	Ras guanyl-nucleotide exchange factor activity	83	2	0.06	0.0013
3	GO:0017089	glycolipid transporter activity	4	1	0	0.0027
4	GO:0005085	guanyl-nucleotide exchange factor activity	144	2	0.1	0.0038
5	GO:0050998	nitric-oxide synthase binding	6	1	0	0.0040
6	GO:0019894	kinesin binding	12	1	0.01	0.0079
7	GO:0051861	glycolipid binding	15	1	0.01	0.0099
8	GO:0019905	syntaxin binding	25	1	0.02	0.0165
9	GO:1901505	carbohydrate derivative transporter activity	27	1	0.02	0.0178
10	GO:0000149	SNARE binding	31	1	0.02	0.0204
11	GO:0030695	GTPase regulator activity	393	2	0.26	0.0264
12	GO:0060589	nucleoside-triphosphatase regulator activity	401	2	0.27	0.0274
13	GO:0008092	cytoskeletal protein binding	495	2	0.33	0.0405
14	GO:0005200	structural constituent of cytoskeleton	65	1	0.04	0.0424
15	GO:0005319	lipid transporter activity	70	1	0.05	0.0456
16	GO:0005509	calcium ion binding	562	2	0.37	0.051
17	GO:0004222	metalloendopeptidase activity	97	1	0.06	0.0626
18	GO:0030234	enzyme regulator activity	792	2	0.53	0.0935
19	GO:0098772	molecular function regulator	855	2	0.57	0.1066
20	GO:0019901	protein kinase binding	169	1	0.11	0.1069
**type C**						
1	GO:0001846	opsonin binding	7	1	0	0.0031
2	GO:0019841	retinol binding	10	1	0	0.0044
3	GO:0043395	heparan sulfate proteoglycan binding	11	1	0	0.0049
4	GO:0043014	alpha-tubulin binding	12	1	0.01	0.0053
5	GO:0043394	proteoglycan binding	15	1	0.01	0.0066
6	GO:0005001	transmembrane receptor protein tyrosine	16	1	0.01	0.0071
7	GO:0019198	transmembrane receptor protein phosphatase activity	16	1	0.01	0.0071
8	GO:0045296	cadherin binding	20	1	0.01	0.0088
9	GO:0005501	retinoid binding	24	1	0.01	0.0106
10	GO:0019840	isoprenoid binding	26	1	0.01	0.0115
11	GO:0017137	Rab GTPase binding	33	1	0.01	0.0145
12	GO:0001948	glycoprotein binding	43	1	0.02	0.0189
13	GO:0043178	alcohol binding	65	1	0.03	0.0284
14	GO:0017016	Ras GTPase binding	84	1	0.04	0.0366
15	GO:0031267	small GTPase binding	93	1	0.04	0.0405
16	GO:0050839	cell adhesion molecule binding	94	1	0.04	0.0409
17	GO:0004725	protein tyrosine phosphatase activity	95	1	0.04	0.0413
18	GO:0004222	metalloendopeptidase activity	97	1	0.04	0.0422
19	GO:0015631	tubulin binding	98	1	0.04	0.0426
20	GO:0051020	GTPase binding	106	1	0.05	0.0460

	GO ID	Term	Annotated	Significant	Expected	P-value (Classic Fisher)
**Antartic**						
1	GO:0001735	prenylcysteine oxidase activity	1	1	0.01	0.0057
2	GO:0008555	chloride-transporting ATPase activity	1	1	0.01	0.0057
3	GO:0030215	semaphorin receptor binding	1	1	0.01	0.0057
4	GO:0051990	(R)-2-hydroxyglutarate dehydrogenase activity	1	1	0.01	0.0057
5	GO:0004605	phosphatidate cytidylyltransferase activity	2	1	0.01	0.0113
6	GO:0003876	AMP deaminase activity	3	1	0.02	0.0170
7	GO:0004332	fructose-bisphosphate aldolase activity	3	1	0.02	0.0170
8	GO:0004339	glucan "1,4-alpha-glucosidase" activity	3	1	0.02	0.0170
9	GO:0005250	A-type (transient outward) potassium channel activity	3	1	0.02	0.0170
10	GO:0008384	IkappaB kinase activity	3	1	0.02	0.0170
11	GO:0016149	translation release factor activity, codon specific	3	1	0.02	0.0170
12	GO:0047623	adenosine-phosphate deaminase activity	3	1	0.02	0.0170
13	GO:0003696	satellite DNA binding	4	1	0.02	0.0225
14	GO:0004689	phosphorylase kinase activity	4	1	0.02	0.0225
15	GO:0008853	exodeoxyribonuclease III activity	4	1	0.02	0.0225
16	GO:0019237	centromeric DNA binding	4	1	0.02	0.0225
17	GO:0004396	hexokinase activity	5	1	0.03	0.0281
18	GO:0003747	translation release factor activity	6	1	0.03	0.0336
19	GO:0004704	NF-kappaB-inducing kinase activity	6	1	0.03	0.0336
20	GO:0008079	translation termination factor activity	6	1	0.03	0.0336
**resident**						
1	GO:0052689	carboxylic ester hydrolase activity	65	3	0.2	0.00099
2	GO:0004623	phospholipase A2 activity	24	2	0.07	0.00236
3	GO:0016298	lipase activity	89	3	0.27	0.00244
4	GO:0004757	sepiapterin reductase activity	1	1	0	0.00303
5	GO:0015633	zinc transporting ATPase activity	1	1	0	0.00303
6	GO:0031492	nucleosomal DNA binding	1	1	0	0.00303
7	GO:0030984	kininogen binding	2	1	0.01	0.00604
8	GO:0003774	motor activity	131	3	0.04	0.0072
9	GO:0038024	cargo receptor activity	56	2	0.17	0.01241
10	GO:0005087	Ran guanyl-nucleotide exchange factor activity	5	1	0.02	0.01504
11	GO:0015321	sodium-dependent phosphate transmembrane transporter activity	5	1	0.02	0.01504
12	GO:0004197	cysteine-type endopeptidase activity	68	2	0.21	0.01795
13	GO:0031491	nucleosome binding	6	1	0.02	0.01802
14	GO:0004033	aldo-keto reductase (NADP) activity	7	1	0.02	0.02100
15	GO:0047498	calcium-dependent phospholipase A2 activity	7	1	0.02	0.02100
16	GO:0004620	phospholipase activity	76	2	0.23	0.02211
17	GO:0003810	protein-glutamine gamma-glutamyltransferase activity	8	1	0.02	0.02396
18	GO:0005088	Ras guanyl-nucleotide exchange factor activity	83	2	0.25	0.02606
19	GO:0032395	MHC class II receptor activity	9	1	0.03	0.02692
20	GO:0033613	activating transcription factor binding	9	1	0.03	0.02692
**transient**						
1	GO:0003905	alkylbase DNA N-glycosylase activity	1	1	0.01	0.0062
2	GO:0004122	cystathionine beta-synthase activity	1	1	0.01	0.0062
3	GO:0004349	glutamate 5-kinase activity	1	1	0.01	0.0062
4	GO:0004350	glutamate-5-semialdehyde dehydrogenase activity	1	1	0.01	0.0062
5	GO:0008434	calcitriol receptor activity	1	1	0.01	0.0062
6	GO:0008470	isovaleryl-CoA dehydrogenase activity	1	1	0.01	0.0062
7	GO:0015019	heparan-alpha-glucosaminide N-acetyltransferase activity	1	1	0.01	0.0062
8	GO:0019202	amino acid kinase activity	1	1	0.01	0.0062
9	GO:0035538	carbohydrate response element binding	1	1	0.01	0.0062
10	GO:0042602	riboflavin reductase (NADPH) activity	1	1	0.01	0.0062
11	GO:0046316	gluconokinase activity	1	1	0.01	0.0062
12	GO:0050197	phytanate-CoA ligase activity	1	1	0.01	0.0062
13	GO:0070251	pristanate-CoA ligase activity	1	1	0.01	0.0062
14	GO:0070404	NADH binding	1	1	0.01	0.0062
15	GO:0071164	RNA trimethylguanosine synthase activity	1	1	0.01	0.0062
16	GO:0004872	receptor activity	1341	16	8.31	0.0076
17	GO:0001608	G-protein coupled nucleotide receptor activity	25	2	0.16	0.0104
18	GO:0045028	G-protein coupled purinergic nucleotide	25	2	0.16	0.0104
19	GO:0000213	tRNA-intron endonuclease activity	2	1	0.01	0.0124
20	GO:0002046	opsin binding	2	1	0.01	0.0124

Supplementary Table 9. Estimates of mean nucleotide diversity (π).

	Sites covered in at least 5 individuals			Genome-wide means using scaffolds with at least 1,000 sites with sequencing coverage for all individuals
	50 Kb windows with 10 Kb slides	100 Kb windows with 10 Kb slides	200 Kb windows with 50 Kb slides	
transient	0.0021	0.0021	0.0021	0.0029
Resident	0.0014	0.0014	0.0014	0.0015
type B1	0.0025	0.0025	0.0025	0.0028
type B2	0.0028	0.0028	0.0028	0.0027
type C	0.0011	0.0011	0.0011	0.0013

Supplementary Table 10. Lists of genes with associated with the top 0.1% (branches to the resident, transient and most recent common ancestor of the Antarctic types) and top 0.01% (branches leading to type B1, type B2 and type C) population branch statistic (PBS) values. *Note that some genes are listed twice due to different exons of the same gene being outliers*

type B1	**Gene**		**PBS**
	refGene.NM_001130823.1.inc	*DNMT1*	0.439
	refGene.NM_005905.1.inc	*SMAD9*	0.420
	ensGene.ENST00000339092.1.inc	*AAK1*	0.401
	refGene.NM_002714.1	*PPP1R10*	0.390
	refGene.NM_019111.5.inc	*HLA-DRA*	0.389
	refGene.NM_001134665.1.inc	*TRMT10A*	0.377
	refGene.NM_001739.1.inc	*CA5A*	0.375
	knownGene.uc003oey.2.1	*ITPR3*	0.364
	refGene.NM_024600.1	*TMEM204*	0.353
	knownGene.uc003jow.2.1	*NNT*	0.350

type B2	**Gene**		**PBS**
	refGene.NM_001014985.1	*GLTPD2*	0.374
	vegaGene.OTTHUMT00000359877.1.inc	*WDR1*	0.340
	refGene.NM_178468.1.inc	*FAM83C*	0.339
	refGene.NM_002421.1.inc	*MMP1*	0.334
	refGene.NM_014786.1.inc	*ARHGEF17*	0.326
	refGene.NM_181654.1	*CPLX4*	0.315
	ensGene.ENST00000425660.1.inc	*ACTB*	0.313
	knownGene.uc003xjt.1.1.inc	*UNC5D*	0.312
	refGene.NM_032680.1.inc	*CRACR2A*	0.306
	ensGene.ENST00000344135.1.inc	*TRIO*	0.298

type C	**Gene**		**PBS**
	knownGene.uc002ebs.1.1.inc	*ITGAM*	0.552
	ensGene.ENST00000340273.1.inc	*MMP13*	0.505
	ensGene.ENST00000340273.1.inc	*MMP13*	0.481
	ensGene.ENST00000379127.1.inc	*C9orf24*	0.479
	refGene.NM_001163334.1.inc	*SYTL5*	0.467
	refGene.NM_000606.1	*C8G*	0.448
	refGene.NM_152421.1.inc	*FAM69B*	0.432
	refGene.NM_206810.2.inc	*MOG*	0.429
	knownGene.uc002pdl.2.1	*RSPH6A*	0.420
	knownGene.uc010wzl.1.1.inc	*PTPRM*	0.394

Antarctic	Gene		PBS
	knownGene.uc004cjy.2.1	*FBXW5*	2.173
	refGene.NM_001167670.1.inc	*TMEM239*	2.166
	refGene.NM_021059.2	*HIST2H3C*	2.164
	knownGene.uc010zrp.1.1	*RRBP1*	2.152
	knownGene.uc003zbq.2.1	*HEATR7A / Mroh1*	2.126
	refGene.NM_001528.1.inc	*HGFAC*	2.112
	knownGene.uc010zrp.1.1	*RRBP1*	2.092
	refGene.NM_030801.2	*MAGED4*	2.077
	ensGene.ENST00000319338.1	*IGSF22*	2.063
	ensGene.ENST00000319338.1	*IGSF22*	2.055
	refGene.NM_019046.1.inc	*ANKRD16*	2.038
	ensGene.ENST00000325577.1.inc	*RAD1*	2.034
	knownGene.uc001bbg.2.1	*EMC1*	2.033
	knownGene.uc009xbu.1.1	*IL20*	2.025
	refGene.NM_004257.1	*TGFBRAP1*	2.024
	refGene.NM_145691.1.inc	*ATPAF2*	2.020
	knownGene.uc002eqs.2.1	*CES2*	2.016
	ensGene.ENST00000454048.1.inc	*D2HGDH*	1.999
	refGene.NM_015658.1.inc	*NOC2L*	1.997
	knownGene.uc009xcp.1.1.inc	*LAMB3*	1.994
	knownGene.uc002clz.2.1	*IFT140*	1.987
	refGene.NM_175614.1	*NDUFA11*	1.985
	refGene.NM_001008708.1.inc	*CHAC2*	1.965
	refGene.NM_001388.1	*DRG2*	1.962
	knownGene.uc002wji.1.1	*C20orf27*	1.961
	knownGene.uc001qxr.2.1.inc	*TMEM52B*	1.959
	refGene.NM_015164.1	*PLEKHM2*	1.955
	refGene.NM_020982.1	*CLDN9*	1.951
	refGene.NM_152468.1.inc	*TMC8*	1.939
	refGene.NM_015164.1	*PLEKHM2*	1.938
	refGene.NM_031433.1.inc	*MFRP*	1.918
	refGene.NM_032325.1	*EIF1AD*	1.917
	refGene.NM_173506.1	*LYPD4*	1.912
	refGene.NM_001172431.1	*AMPD3*	1.904
	knownGene.uc002eqs.2.1	*CES2*	1.903
	ensGene.ENST00000401649.1.inc	*NOTCH2*	1.899
	knownGene.uc009vsu.1.1.inc	*SYTL1*	1.896
	refGene.NM_007096.1	*CLTA*	1.881
	knownGene.uc002viw.2.1.inc	*USP37*	1.880
	knownGene.uc002eqs.2.1	*CES2*	1.878
	knownGene.uc003zcn.2.1	*SLC39A4*	1.875
	knownGene.uc002wlv.2.1	*CDS2*	1.875
	knownGene.uc010vgi.1.1.inc	*PHKB*	1.873
	refGene.NM_014012.1	*REM1*	1.868
	knownGene.uc011lky.1.1.inc	*WDR97*	1.867

knownGene.uc010rpw.1.1	*NDUFV1*	1.860
knownGene.uc010nip.2.1.inc	*CACNA1F*	1.858
refGene.NM_207348.1	*SLC25A34*	1.856
refGene.NM_018163.1.inc	*DNAJC17*	1.844
refGene.NM_015417.1	*SPEF1*	1.841
refGene.NM_198317.1.inc	*KLHL17*	1.835
refGene.NM_004661.1	*CDC23*	1.833
knownGene.uc002clz.2.1	*IFT140*	1.832
knownGene.uc010vjn.1.1.inc	*ZN423*	1.831
refGene.NM_005830.1	*MRPS31*	1.829
refGene.NM_020650.1	*RCN3*	1.829
refGene.NM_018028.1.inc	*SAMD4B*	1.828
refGene.NM_014757.1.inc	*RG214790*	1.827
vegaGene.OTTHUMT00000373208.1.inc	*GRK6*	1.826
knownGene.uc001ebu.1.1	*KCND3*	1.823
knownGene.uc002enz.1.1.inc	*CNOT1*	1.820
knownGene.uc002gmc.3.1.inc	*USP43*	1.816
refGene.NM_001980.1	*STX2*	1.814
knownGene.uc002esp.3.1.inc	*PLEKHG4*	1.813
refGene.NM_198488.1	*FAM83H*	1.809
refGene.NM_001810.1.inc	*CENPB*	1.807
refGene.NM_172229.1.inc	*KREMEN2*	1.805
knownGene.uc002ixr.1.1.inc	*CLTC*	1.803
knownGene.uc010vyx.1.1.inc	*RNF112*	1.802
knownGene.uc002esp.3.1.inc	*PLEKHG4*	1.797
refGene.NM_014731.1	*LZTS3*	1.797
knownGene.uc001gwc.2.1.inc	*IGFN1*	1.792
refGene.NM_030759.1.inc	*NRBF2*	1.790
refGene.NM_014717.1	*ZNF536*	1.789
refGene.NM_001160184.1	*PLEKHN1*	1.789
refGene.NM_005165.1	*ALDOC*	1.787
ensGene.ENST00000263046.1	*TFAP2B*	1.784
refGene.NM_001100915.1	*KCTD19*	1.784
ensGene.ENST00000448774.1.inc	*PLXNB1*	1.783
knownGene.uc002esy.2.1.inc	*TMEM208*	1.783
refGene.NM_015164.1	*PLEKHM2*	1.781
knownGene.uc003etm.2.1	*CLSTN2*	1.778
knownGene.uc010fdo.2.1	*PCYOX1*	1.777
knownGene.uc001bfv.1.1.inc	*ZBTB40*	1.775
refGene.NM_004214.1	*FIBP*	1.772
refGene.NM_015457.1	*ZDHHC5*	1.771
ensGene.ENST00000319338.1	*IGSF22*	1.767
refGene.NM_021044.1	*DHH*	1.765
refGene.NM_198317.1.inc	*KLHL17*	1.759
refGene.NM_001008910.1	*STK16*	1.749
ensGene.ENST00000438091.1.inc	*IL17RC*	1.748

refGene.NM_024784.1.inc	*ZBTB3*	1.747
refGene.NM_003407.1	*ZFP36*	1.746
refGene.NM_015168.1	*ZC3H4*	1.744
knownGene.uc002eqs.2.1	*CES2*	1.739
knownGene.uc002eqs.2.1	*CES2*	1.739
ensGene.ENST00000420190.1.inc	*SAMD11*	1.729
knownGene.uc009wqr.1.1.inc	*ASH1L*	1.726
refGene.NM_014831.1.inc	*TRANK1*	1.724
ensGene.ENST00000448221.1.inc	*NFKBIL2 / TONSL*	1.723
knownGene.uc003mez.2.1.inc	*HK3*	1.722
refGene.NM_001164766.1.inc	*ZFHX3*	1.719
refGene.NM_001114184.1.inc	*MTRF1L*	1.716

transient	Gene		PBS
	vegaGene.OTTHUMT00000365743.1.inc		1.521
	refGene.NM_018181.1.inc	*ZNF532*	1.164
	knownGene.uc010qoh.1.1	*ALDH18A1*	1.114
	refGene.NM_002049.1.inc	*GATA1*	1.114
	refGene.NM_014587.1	*SOX8*	1.112
	ensGene.ENST00000453997.1.inc	*MB*	1.031
	refGene.NM_004312.1.inc	*ARR3*	1.006
	knownGene.uc011mmb.1.1.inc	*TBC1D25*	1.006
	refGene.NM_000185.1.inc	*SERPIND1*	0.996
	ensGene.ENST00000395426.1.inc	*SLC38A4*	0.986
	knownGene.uc004czb.2.1.inc	*ADGRG2*	0.963
	refGene.NM_005448.1	*BMP15*	0.961
	refGene.NM_014058.2.inc	*TMPRSS11E*	0.920
	refGene.NM_014008.1.inc	*CCDC22*	0.896
	refGene.NM_015685.1	*SDCBP2*	0.852
	refGene.NM_000532.1.inc	*PCCB*	0.849
	refGene.NM_000713.1	*BLVRB*	0.844
	ensGene.ENST00000437780.1	*PASK*	0.826
	refGene.NM_001164436.1.inc	*TMEM212*	0.822
	refGene.NM_002507.1.inc	*NGFR*	0.820
	refGene.NM_024966.1	*SEMA6D*	0.807
	knownGene.uc001ncf.2.1.inc	*SPI1*	0.802
	ensGene.ENST00000401672.1.inc	*PPP6R2*	0.795
	refGene.NM_006612.1	*KIF1C*	0.779
	ensGene.ENST00000398168.1.inc	*CBS*	0.768
	refGene.NM_018182.1	*FAM222B*	0.766
	refGene.NM_022826.1.inc	*MARCH7*	0.766
	refGene.NM_003645.1	*SLC27A2*	0.759
	knownGene.uc002hsl.2.1	*ERBB2*	0.758
	refGene.NM_001161416.1	*GPR17*	0.755
	refGene.NM_022475.1	*HHIP*	0.754
	refGene.NM_003189.1	*TAL1*	0.753
	refGene.NM_031459.1.inc	*SESN2*	0.749
	refGene.NM_015140.1.inc	*TTLL12*	0.747
	refGene.NM_014467.1.inc	*SRPX2*	0.743
	refGene.NM_145051.1	*RNF183*	0.738
	refGene.NM_002225.1.inc	*IVD*	0.733
	refGene.NM_002125.1.inc	*HLA*	0.706
	refGene.NM_001616.1.inc	*ACVR2A*	0.701
	refGene.NM_006466.1	*POLR3F*	0.696
	knownGene.uc010rgx.1.1	*C11orf49*	0.693
	refGene.NM_032370.1	*ZNF414*	0.688
	refGene.NM_003041.1	*SLC5A2*	0.683

knownGene.uc004dyf.1.1.inc	*KIF4A*	0.673
knownGene.uc004ayt.2.1	*ANKS6*	0.671
refGene.NM_032737.1.inc	*LMNB2*	0.670
refGene.NM_021209.1.inc	*NLRC4*	0.663
refGene.NM_015140.1.inc	*TTLL12*	0.661
refGene.NM_001172557.1.inc	*GOLGA3*	0.644
refGene.NM_001409.1.inc	*MEGF6*	0.639
ensGene.ENST00000370100.1	*SRPK3*	0.638
refGene.NM_018190.1.inc	*BBS7*	0.638
ensGene.ENST00000453275.1.inc	*BHLHD14*	0.637
refGene.NM_001077446.1	*TSEN34*	0.631
refGene.NM_001004439.1	*ITGA11*	0.631
knownGene.uc003dek.1.1.inc	*STAB1*	0.627
knownGene.uc010zkq.1.1.inc	*CCDC108*	0.625
ensGene.ENST00000438774.1.inc	*TMEM151B*	0.621
refGene.NM_001409.1.inc	*MEGF6*	0.617
refGene.NM_001943.1.inc	*DSG2*	0.616
knownGene.uc002ilw.1.1.inc	*OSBPL7*	0.609
knownGene.uc010wzv.1.1	*SLMO1*	0.602
refGene.NM_022119.1.inc	*PRSS22*	0.599
refGene.NM_024109.1.inc	*METTL22*	0.598
knownGene.uc009zxh.2.1.inc	*TMEM120B*	0.594
refGene.NM_014750.1	*DLGAP5*	0.590
knownGene.uc011mog.1.1	*RIBC1*	0.589
refGene.NM_001015052.1.inc	*MPG*	0.587
knownGene.uc002gbz.2.1	*DHX33*	0.587
knownGene.uc001kqm.3.1	*SFTPA2*	0.574
knownGene.uc002fxk.1.1.inc	*ZZEF1*	0.574
refGene.NM_022150.1.inc	*NPVF*	0.571
knownGene.uc003qwz.1.1.inc	*WDR27*	0.567
refGene.NM_003667.1	*LGR5*	0.566
refGene.NM_153836.1.inc	*CREG2*	0.556
knownGene.uc001poi.2.1	*USP28*	0.552
refGene.NM_001081003.1.inc	*COMMD5*	0.548
knownGene.uc010nzu.1.1.inc	*DNAJC11*	0.547
refGene.NM_001013.1.inc	*RPS9*	0.542
knownGene.uc010lyh.2.1.inc	*TGS1*	0.540
refGene.NM_145267.1	*SDHAF4*	0.540
refGene.NM_001114632.1	*JMJD7*	0.536
refGene.NM_005073.1.inc	*SLC15A1*	0.531
knownGene.uc010dsl.2.1	*ADAMTS*	0.530
refGene.NM_001098202.1	*HIC1*	0.528
refGene.NM_002081.1.inc	*GPC1*	0.524
refGene.NM_001130043.1	*CRYZ*	0.519
refGene.NM_023915.1	*GPR87*	0.515
refGene.NM_000376.1	*VDR*	0.512

refGene.NM_003212.1.inc	*TDGF1*	0.511
knownGene.uc002mje.2.1	*FBN3*	0.511
knownGene.uc010ptv.1.1	*RPS6KC1*	0.509
knownGene.uc003jow.2.1	*ITGA1*	0.508
refGene.NM_152419.1.inc	*HGSNAT*	0.507
knownGene.uc003gfa.1.1.inc	*BC010180*	0.506
refGene.NM_018964.1	*SLC37A1*	0.504
knownGene.uc011ljc.1.1.inc	*TG*	0.504
refGene.NM_002862.1.inc	*PYGB*	0.504
knownGene.uc004amu.1.1.inc	*GKAP1*	0.503
ensGene.ENST00000254271.1.inc	*LRRC9*	0.502
ensGene.ENST00000436299.1.inc	*EPHB6*	0.500
knownGene.uc003zoh.1.1	*FOCAD*	0.500
refGene.NM_001029863.1.inc	*C6orf120*	0.496

resident	**Gene**		**PBS**
	ensGene.ENST00000437387.1.inc	*MYO7B*	2.089
	refGene.NM_174944.1.inc	*TSSK4*	1.926
	ensGene.ENST00000436581.1	*RUNX1T1*	1.918
	refGene.NM_153646.1	*SLC24A4*	1.874
	refGene.NM_007073.1.inc	*BVES*	1.868
	refGene.NM_006693.1.inc	*CPSF4*	1.846
	ensGene.ENST00000437387.1.inc	*MYO7B*	1.741
	ensGene.ENST00000379274.1.inc	*DGKH*	1.740
	ensGene.ENST00000373702.1	*DOCK10*	1.737
	refGene.NM_015335.1	*MED13L*	1.730
	knownGene.uc003qhy.2.1.inc	*IL20RA*	1.727
	refGene.NM_147780.1.inc	*CTSB*	1.687
	refGene.NM_002125.1.inc	*HLA-DRB5*	1.679
	knownGene.uc010tog.1.1.inc	*TGM1*	1.616
	refGene.NM_018838.1	*NDUFA12*	1.603
	refGene.NM_213720.1.inc	*CHCHD10*	1.559
	knownGene.uc003lll.2.1.inc	*ARAP3*	1.542
	knownGene.uc010wii.1.1	*ETV4*	1.514
	refGene.NM_181643.1.inc	*PIFO*	1.493
	knownGene.uc010nip.2.1.inc	*CACNA1F*	1.491
	ensGene.ENST00000433625.1.inc	*EFHC1*	1.475
	refGene.NM_018842.1.inc	*BAIAP2L1*	1.464
	ensGene.ENST00000370864.1.inc	*TINAG*	1.462
	ensGene.ENST00000379274.1.inc	*DGKH*	1.462
	knownGene.uc003qrb.2.1	*SERAC1*	1.461
	refGene.NM_138441.1.inc	*MB21D1*	1.453
	knownGene.uc010tjh.1.1.inc	*TEP1*	1.453
	refGene.NM_015715.1.inc	*PLA2G3*	1.452
	refGene.NM_001048199.1	*RCC1*	1.451
	ensGene.ENST00000288709.1.inc	*MMEL1*	1.447
	knownGene.uc010voj.1.1	*KIAA0513*	1.446
	refGene.NM_080821.1.inc	*FAM210B*	1.394
	refGene.NM_003459.1.inc	*SLC30A3*	1.365
	refGene.NM_003052.1	*SLC34A1*	1.363
	refGene.NM_001001795.1.inc	*LRRC24*	1.355
	knownGene.uc003wub.1.1	*GATA4*	1.351
	ensGene.ENST00000395536.1.inc	*AKAP10*	1.340
	refGene.NM_002336.1.inc	*LRP6*	1.326
	knownGene.uc010ztr.1.1.inc	*TM9SF4*	1.320
	refGene.NM_174905.1.inc	*FAM98C*	1.314
	knownGene.uc002bwq.1.1.inc	*LRRK1*	1.304
	knownGene.uc002lmk.1.1.inc	*ZNF236*	1.301
	ensGene.ENST00000476379.1	*CCDC39*	1.298

refGene.NM_171982.1.inc	*TRIM35*	1.291
knownGene.uc010udj.1.1	*STARD9*	1.290
refGene.NM_139179.1	*DAGLB*	1.285
refGene.NM_003447.1	*OR1F12*	1.271
refGene.NM_024658.1	*IPO4*	1.255
refGene.NM_002353.1	*TACSTD2*	1.247
refGene.NM_139179.1	*DAGLB*	1.242
refGene.NM_178034.1	*PLA2G4D*	1.230
knownGene.uc001oke.1.1	*ANKRD13D*	1.223
refGene.NM_014976.1	*PDCD11*	1.219
knownGene.uc001qfl.2.1.inc	*PRDM10*	1.216
refGene.NM_003124.1.inc	*SPR*	1.211
ensGene.ENST00000254271.1.inc	*LRRC9*	1.207

Supplementary Notes

A brief natural history of the study species

The killer whale is emerging as a useful organism for studying adaptation and speciation, as the phenotype, biogeography and ecology underlying evolutionary divergence and the genetic outcome in terms of neutral genetic differentiation are well described[13,78]. Dietary differences have been studied through: direct observation of naturally marked, site-faithful individuals over many years; multi-chemical markers such as stable isotope and fatty acids; and molecular and visual identification of prey remains from predation events, faecal samples and stomach contents[7-10,79-89]. Morphology has been described qualitatively and quantitatively. For example, body length has been measured directly from stranded and captive specimens or those taken by whaling operations, or from free-ranging live specimens using laser-metrics and aerial photogrammetry[10,90-94]; and pigmentation features have been qualitatively and quantitatively compared among populations from photographic data[9,95-98].

Four decades of dedicated research in the North Pacific have characterized three ecotypes to date: a mammal-eating specialist commonly referred to the '*transient*' ecotype as (more recent studies have referred to this ecotype as Bigg's killer whale, named after the biologist Michael Bigg, who pioneered modern killer whale research and made studies like this one possible[99]); a fish-eating specialist commonly referred to as the '*resident*' ecotype; and a third Pacific ecotype is most frequently encountered in waters further offshore but on the continental shelf slope and is known to have a diet that includes sharks and other fish, and is commonly referred to as the so-called '*offshore*' ecotype[88,100]. Observations of social interactions between different ecotypes are extremely rare and the observations of encounters between the *resident* and *transient* ecotypes indicate that the *transient* ecotype will typically change travel patterns to avoid the *resident* ecotype and that occasionally *residents* display antagonistic behaviour towards *transients*[101-103]. There are morphological differences between these three North Pacific ecotypes including overall body size and the shape of the dorsal fin and saddle patch[95,104].

Long-term studies of naturally marked individuals have detected no dispersal between North Pacific ecotypes and no dispersal from the natal matrilineal social in the

resident ecotype[99,104]. There is some dispersal of *transients* from the natal group[102,105]. The lack of dispersal between ecotypes appears to have been maintained over longer timescales than the field studies based on lineage sorting of mitochondrial genomes and significant differentiation between ecotypes based on microsatellite allele frequencies[11,106-109]. Better resolution on the timing and extent of any gene flow between North Pacific ecotypes is needed. Phylogeographic analyses based on mitogenome sequences indicate that the *resident* and *offshore* ecotypes share a more recent common ancestor with lineages of Northeast Atlantic killer whales than with the *transient* ecotype and are consistent with sympatry between the North Pacific ecotypes arising from secondary contact following an allopatric phase[110]. However, phylogenies based on nuclear loci are to some extent discordant with the mitochondrial phylogeny[22], possibly due to low levels of gene flow between ecotypes within the same ocean basin, which may have occurred upon primary or secondary contact, making it difficult to discern between these two scenarios[111]. There are several genetically differentiated populations of the *resident* and *transient* ecotype in the North Pacific[106-108], but to date only one population of the *offshore* ecotype has been identified[106,107].

Killer whales in the waters around the Antarctic continent have diversified into several distinct morphotypes partially overlapping in their ranges[9,10,81,82]. Killer whales in Antarctic waters with the pigmentation patterns that most closely resemble the common killer whale colouration are morphologically classified as *type A*[9], but this classification does not infer genetic or ecologically cohesiveness. The Antarctic morphotypes included in this study (*types B1*, *B2* & *C*) differ from *type A* as they have a discernable dorsal cape[9,10]. *Types B1* and *B2* have a large eye patch[9,10], whereas *type C* has a smaller forward-slanted eye patch[9]. Body size also varies, with photogrammetry measurements indicating that *type B2* is smaller than *type B1*[10], and that *type C* grow up to just 5.6 meters in length, making this the smallest form of killer whale measured to date[94].

Field observations and stable isotope measurements indicate that there are differences in the preferred habitat and prey of each of the Antarctic types[9,10,81,82,85]. *Type B1* is commonly observed in the pack-ice hunting Weddell seals (*Leptonychotes weddellii*)[10,82], whilst *type B2* forages in more open water, observed killing and eating

penguins[10,83]; *type C* is most commonly observed in the dense pack-ice and its diet is known from observations to include Antarctic toothfish (*Dissostichus mawsoni*) and based on stomach contents from Soviet whaling data is thought to be primarily pisciverous[9]. Most observations of these Antarctic types have been made during the Austral summer, however, there are some observations of *type B* and *type C* in the Antarctic pack-ice during the Austral winter[9]. There have also been occasional sightings of these Antarctic types at higher latitudes[9] and satellite-tagging data indicates that they make rapid round-trip movements from Antarctic waters to subtropical waters and back, hypothesized to be for skin generation in warmer waters[42].

Genetic differentiation based on microsatellite allele frequencies is relatively low between *type B* and *type C* ($G'_{ST} = 0.11$) compared with differentiation between the North Pacific *resident* and *transient* ecotypes ($G'_{ST} = 0.28$)[112]. Mitogenome phylogenetic analyses indicate that *type B* (both *B1* and *B2*) and type C are reciprocally monophyletic, suggesting that there is little or no permanent dispersal between types[11,112], with the exception of a single sampled type B1individual[11]. Comparison of amino acid substitutions across the mitogenome identified two non-synonymous changes resulting in localized changes in polarity that putatively occurred under natural selection within the *cytochrome b* gene[113]. One change had reached fixation in *type B* killer whales (both *B1* and *B2*) and the other was close to fixation in *type C*[113]. The changes were at different sites and in the opposite direction in each type, suggesting divergent evolution since *types B1*, *B2* and *C* diverged from their most recent common ancestor. All other substitutions across the mitogenome in a global killer whale dataset appeared to have evolved under neutrality[113].

The published data cited here indicate that these populations of killer whales are ecologically, morphologically and genetically divergent, and thus generally meet the criteria for most of the many definitions of the term ‘ecotype’ that have been proposed over the years[114]. The term ecotype is therefore adopted here. The behavioural adaptations that each ecotype uses to exploit an ecological niche are thought to be passed on from one generation to the next by social learning within matrilineal groups[12,13]. These behavioural adaptations include: coordinated ‘wave-washing’ behaviour by *type B1* killer whales in Antarctica to dislodge seals from ice

floes[82]; 'carousel-feeding', whereby killer whales in some North Atlantic populations are reported to co-ordinately herd herring schools into a tight ball by encircling them, flashing their white undersides, emitting large bubbles and producing a low frequency pulsed call, prior to tail-slapping the herded herring to stun them[115,116]; and intentional stranding on to the beach to catch seals performed by some killer whale social groups at the Crozet Archipelago in the Southern Ocean[117,118]. Perhaps due to the complexity and cumulative nature of human culture and due to it being the focus of study in a range of fields from anthropology to zoology, there is no clear definitional consensus of the term 'culture'[1]. For the purposes of investigating how cultural phenomena interact with genes, a recent review suggested that "culture is information that is capable of affecting individuals' behaviour, which they acquire from other individuals through teaching, imitation and other forms of social learning"[1]. Under this definition, several studies have argued that socially learned foraging behaviours within killer whale ecotypes should be considered as examples of culture in this broader sense of the term[13,14,119].

Supplementary References

70. Grabherr, M. G. *et al*. Genome-wide synteny through highly sensitive sequence alignment: Satsuma. *Bioinformatics* **26**, 1145–1151 (2010).
71. Foote A. D. *et al*. Tracking niche variation over millennial timescales in sympatric killer whale lineages *Proc. R. Soc. B* **280,** 20131481 (2013).
72. Vieira, F. G., Lassalle, F., Korneliussen, T. S. & Fumagalli, M. Improving the estimation of genetic distances from Next-Generation Sequencing data. *Biol. J. Linnean Soc.* **117,** 139-149 (2015).
73. Lefort, V., Desper, R. & Gascuel, O. FastME 2.0: a comprehensive, accurate, and fast distance-based phylogeny inference program. *Mol. Biol. Evol.* **32,** 2798-2800 (2015).
74. Korneliussen, T. S. & Moltke, I. NgsRelate: a software tool for estimating pairwise relatedness from next-generation sequencing data. *Bioinformatics* **31,** 4009-4011.
75. Durand, E. Y., Patterson, N., Reich, D. & Slatkin, M. Testing for ancient admixture between closely related populations. *Mol. Biol. Evol.* **28**, 2239–2252 (2011).
76. Orlando, L. *et al*. Recalibrating *Equus* evolution using the genome sequence of an early Middle Pleistocene horse. *Nature* **499,** 74–78 (2013).
77. Staab, P. R., Zhu, S., Metzler, D. & Lunter, G. scrm: efficiently simulating long sequences using the approximated coalescent with recombination. *Bioinformatics* **31**, 1680–1682 (2015).
78. Foote A. D. Investigating ecological speciation in non-model organisms: a case study on killer whale ecotypes. *Evol. Ecol. Res*. **14**, 447–465 (2012).
79. Matkin C. O. *et al*. Ecotypic variation and predatory behavior among killer whales (*Orcinus orca*) off the eastern Aleutian Islands, Alaska. *Fish. Bull.* **105**, 74–87 (2007).
80. Filatova O. A. *et al*. Reproductively isolated ecotypes of killer whales *Orcinus orca* in the seas of the Russian Far East. *Biology Bulletin* **42**, 674–681 (2015).
81. Pitman R. L. & Durban, J. W. Killer whale predation on penguins in Antarctica. *Polar Biol.* **33**, 1589–1594 (2010).
82. Pitman R. L. & Durban, J. W. Cooperative hunting behavior, prey selectivity and prey handling by pack ice killer whales (*Orcinus orca*), type B, in Antarctic Peninsula waters. *Mar. Mamm. Sci.* **28**, 16–36 (2012).
83. Foote A. D. *et al*. Ecological, morphological and genetic divergence of sympatric North Atlantic killer whale populations. *Mol. Ecol.* **18**, 5207–5217 (2009).
84. Herman D. P. *et al*. Feeding ecology of eastern North Pacific killer whales *Orcinus orca* from fatty acid, stable isotope, and organochlorine analyses of blubber biopsies. *Mar. Ecol. Prog. Ser.* **302**, 275–291(2005).
85. Krahn M. M. *et al*. Use of chemical tracers in assessing the diet and foraging regions of eastern North Pacific killer whales. *Mar. Environ. Res.* **63**, 91–114 (2007).
86. Rice, D. W. Stomach contents and feeding behaviour of killer whales in the eastern North Pacific. *Norsk Hval-fangst Tidskr* **57**, 35–38 (1968).
87. Matthews, C. J. D. & Ferguson, S. H. Spatial segregation and similar trophic level diet among eastern Canadian Arctic/ north-west Atlantic killer whales inferred from bulk and compound specific isotopic analysis. *J. Mar. Biol. Assoc. UK*. **94,** 1343–1355 (2014).
88. Ford J. K. B. *et al*. Shark predation and tooth wear in a population of

northeastern Pacific killer whales. *Aquat. Biol.* **11**, 213–224 (2011).
89. Krahn M. M. *et al*. Use of chemical tracers to assess diet and persistent organic pollutants in Antarctic Type C killer whales. *Mar. Mam. Sci.* **24**, 643–663 (2008).
90. Christensen, I. Growth and reproduction of killer whales, *Orcinus orca*, in Norwegian coastal waters. *Rep. Int. Whaling Comm.* Special Issue **6**, 253–258 (1984).
91. Duffield, D. A. & Miller, K. W. Demographic features of killer whales in oceanaria in the United States and Canada, 1965–1987. *Rit Fisk.* **11**, 297–306 (1988).
92. Durban, J. W. & Parsons, K. M. Laser-metrics of free-ranging killer whales. *Mar. Mamm. Sci.* **22**, 735–743 (2006).
93. Pitman R. L. *et al*. A dwarf form of killer whale in Antarctica. *J. Mammal.* **88**, 43–48 (2007).
94. Fearnbach H. *et al*. Size and long-term growth trends of Endangered fish-eating killer whales. *Endang. Species Res.* **13**, 173–180 (2011).
95. Baird, R. W. & Stacey, P. J. Variation in saddle patch pigmentation in populations of killer whales (*Orcinus orca*) from British Columbia, Alaska, and Washington State. *Can. J. Zool.* **66**, 2582–2585 (1988).
96. Visser, I. N. & Mäkeläinen, P. Variation in eye-patch shape of killer whales (*Orcinus orca*) in New Zealand waters. *Mar. Mamm. Sci.* **16**, 459–469 (2000).
97. Pitman R. L. *et al*. Observations of a distinctive morphotype of killer whale (*Orcinus orca*), type D, from subantarctic waters. *Polar Biol.* **34**, 303–306 (2011).
98. Mäkeläinen P. *et al*. A comparison of pigmentation features among North Atlantic killer whale (*Orcinus orca*) populations. *J. Mar. Biol. Assoc. UK.* **94**, 1335–1341 (2014).
99. Bigg, M. A., Olesiuk, P. F., Ellis, G. M., Ford, J. K. B. & Balcomb, K. C. Social organization and genealogy of resident killer whales (*Orcinus orca*) in the coastal waters of British Columbia and Washington State. *Rep. Int. Whal. Comm. (special issue)* **12,** 383-405 (1990).
100. Dahlheim, M. E., Schulman-Janiger, A., Black, N., Ternullo, R. Ellifrit, D. & Balcomb III, K. C. Eastern temperate North Pacific offshore killer whales (*Orcinus orca*): occurrence, movements, and insights into feeding ecology. *Mar. Mamm. Sci.* **24,** 719-729 (2008).
101. Morton, A. B. A quantitative comparison of the behaviour of resident and transient forms of killer whales off the central British Columbia coast. *Rep. Int. Whaling Commn.* **12**, 245–248 (1990).
102. Ford, J. K. B. & Ellis, G. M. *Transients: mammal-hunting killer whales of British Columbia, Washington, and southeastern Alaska.* (UBC Press, 1999).
103. Baird, R. W. & Dill, L. M. Occurrence and behavior of transient killer whales: seasonal and pod-specific variability, foraging behavior and prey handling. *Can. J. Zool.* **73,** 1300–1311 (1995).
104. Ford, J. K. B. Ellis, G. M. & Balcomb, K. C. *Killer whales: the natural history and genealogy of Orcinus orca in British Columbia and Washington.* (UBC Press, 2000).
105. Baird, R. W. & Whitehead, H. Social organization of mammal-eating killer whales: group stability and dispersal patterns. *Can. J. Zool.* **78**, 2096–2105 (2000).
106. Barrett-Lennard, L. G. Population structure and mating patterns of killer whales (*Orcinus orca*) as revealed by DNA analysis. PhD thesis (2000).

107. Hoelzel, A. R. Evolution of population structure in a highly social top predator, the killer whale. *Mol. Biol. Evol.* **76**, 1407-1415 (2007).
108. Parsons, K. M. *et al.* Geographic patterns of genetic differentiation among killer whales in the northern North Pacific. *J. Hered.* **104**, 737–754 (2013).
109. Ford M. J. *et al.* Inferred paternity and male reproductive success in a killer whale (*Orcinus orca*) population. *J. Hered.* **102**, 537–553 (2011).
110. Foote A. D. *et al.* Out of the Pacific and back again: the matrilineal history of Pacific killer whale ecotypes. *PLoS ONE* **6**, e24980 (2011).
111. Foote, A. D. & Morin, P. A. Sympatric speciation in killer whales? *Heredity* **114**, 537–538 (2015).
112. Morin, P. A. *et al.* Complete mitochondrial genome phylogeographic analysis of killer whales (*Orcinus orca*) indicates multiple species. *Genome Res.* **20**, 908–916 (2010).
113. Foote *et al.* A. D. Positive selection on the killer whale mitogenome. *Biol. Lett.* **7**, 116–118 (2011).
114. Lowry, D. B. Ecotypes and the controversy over stages in the formation of new species. *Biol. J. Linn. Soc.* **106**, 241–257 (2012).
115. Similä, T. & Ugarte, F. Surface and underwater observations of cooperatively feeding killer whales in northern Norway. *Can. J. Zool.* **71**, 1494-1499 (1993).
116. Simon M. *et al.* Icelandic killer whales *Orcinus orca* use a pulsed call suitable for manipulating the schooling behaviour of herring *Clupea harengus*. *Bioacoustics* **16**, 57–74 (2006).
117. Guinet, C. Intentional stranding apprenticeship and social play in killer whales (*Orcinus orca*). *Can. J. Zool.* **69**, 2712–2716 (1991).
118. Guinet, C. & Bouvier, J. Development of intentional stranding hunting techniques in killer whale (*Orcinus orca*) calves at Crozet Archipelago. *Can. J. Zool.* **73**, 27–33 (1995).
119. Whitehead, H. Cultural selection and genetic diversity in matrilineal whales. *Science* **282,** 1708–1711 (1998).
